# Canonical and non-canonical miRNA degradation shapes state transitions and stemness in breast cancer

**DOI:** 10.1038/s44318-026-00889-8

**Published:** 2026-08-17

**Authors:** Carmela Rubolino, Manfredi D’Onghia, Chiara Tordonato, Arianna Sabò, Alessandro D’Agnelli, Sara Bisi, Roberto Giambruno, Bianca Giuliani, Matteo Jacopo Marzi, Francesco Nicassio

**Affiliations:** 1 Center for Genomic Science of IIT@SEMM, Istituto Italiano di Tecnologia (IIT) - Via Adamello 16, 20139 Milan, Italy; 2 Department of Experimental Oncology, European Institute of Oncology (IEO) - IRCCS, Milan, Italy; 3 Department of Oncology and Haemato-Oncology, Università degli Studi di Milano, Milan, Italy; 4 Institute for Biomedical Research and Innovation, National Research Council, Palermo, Italy

**Keywords:** Cancer, Post-translational Modifications & Proteolysis, RNA Biology

## Abstract

Target-directed microRNA degradation (TDMD) is an emerging post-transcriptional mechanism that controls miRNA turnover, yet its role in human cancers remains largely unexplored. Here, we combine CRISPRi-mediated ZSWIM8 depletion, miRNA-seq, and AGO2-eCLIP to define the TDMD landscape across breast cancer subtypes. We identify 19 high-confidence TDMD substrates, including miR-29b-3p and miR-33a/b-5p, and show that TDMD shapes miRNA target occupancy and target repression. Integration with single-cell transcriptomics reveals that TDMD of miR-29b-3p triggered by NREP transcript is associated with transcriptional plasticity along the epithelial–mesenchymal axis and marks a stem-like subpopulation of malignat cells with tumor-initiating potential in triple-negative breast cancer. Unexpectedly, we also uncover a non-canonical TDMD mechanism, independent of ubiquitin ligase ZSWIM8 and the proteasome, which includes SERPINE1-triggered miR-30c-5p degradation, conferring paclitaxel resistance and enhancing sphere-forming potential. These findings establish target-directed microRNA degradation as a functional layer of miRNA regulation in cancer, linking miRNA decay to cellular state transitions and therapeutic response. Our results broaden the mechanistic spectrum of TDMD and provide a framework to investigate how regulated miRNA decay contributes to aggressive breast cancer phenotypes.

## Synopsis

Target-directed microRNA degradation (TDMD) is an emerging post-transcriptional mechanism that controls miRNA turnover, yet its role in cancer remains largely unexplored. This study maps endogenous TDMD substrates across human breast cancer cell models and highlights the impact of TDMD on miRNA function in malignant cell plasticity via canonical and non-canonical mechanisms.

- A high confidence TDMD miRNA substrate signature is defined in human breast cancer.
- Depletion of TDMD effector ubiquitin ligase ZSWIM8 remodels miRNA target occupancy in cancer cells.
- TDMD of miR-29b-3p is triggered by *NREP* transcript in mesenchymal breast cancer cells in TWIST1-dependent manner.
- The *NREP*:miR-29b-3p axis contributes to breast cancer cell transcriptional plasticity.
- A ZSWIM8-independent, SERPINE1-mediated non-canonical TDMD mechanism controls miR-30c-5p stability and paclitaxel resistance.

## Abstract (web summary)

A novel catalogue of decay-regulated microRNAs in breast cancer highlights direct roles for target directed miRNA degradation malignant cell plasticity.

## Graphical abstract

**Figure:**
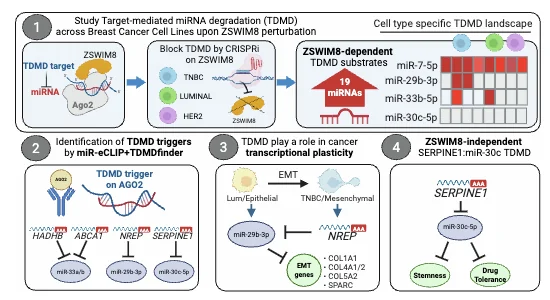

## Introduction

MicroRNAs (miRNAs) are an evolutionarily conserved class of ~22-nucleotide (nt) non-coding RNAs that function in post-transcriptional repression of mRNA targets (Bartel, 2018). MiRNA:target interaction involves usually the seed region (nucleotides 2–8 at the 5’ end of the miRNA) and a complementary region in the target known as the miRNA responsive element (MRE), typically located in the 3’ untranslated region (UTR) of messenger RNAs (mRNAs). MiRNA activity occurs while they are loaded on Argonaute (AGO) proteins, forming the miRNA-induced silencing complex (miRISC), which destabilizes target mRNAs and represses their translation (Jonas and Izaurralde, 2015). In physiology and disease, miRNAs regulate hundreds of target genes, playing a crucial role in controlling many biological processes, cell growth, differentiation, cellular fitness and other (Bartel, 2018; Vidigal and Ventura, 2015). As miRNA activity is primarily dictated by miRNA abundance, levels of miRNAs are finely regulated by a multitude of mechanisms, including both transcriptional and post-transcriptional events (Gebert and MacRae, 2019; Treiber et al, 2019). Recently, it was shown that miRNA levels in cells are controlled by decay (Duffy et al, 2015; Kingston and Bartel, 2019; Marzi et al, 2016; Ruegger and Grosshans, 2012) and a post-transcriptional mechanism named target-directed miRNA degradation (TDMD) has been described (Ameres et al, 2010; de la Mata et al, 2015). TDMD requires a specific pairing of the target mRNA (trigger) with the cognate miRNA (substrate), called the miRNA-degradation element (MDE), involving the seed match at the 5’ end, a central unpaired region (bulge) and an additional complementary sequence match at the 3’ end of the miRNA) (Ameres et al, 2010; Sheu-Gruttadauria et al, 2019). Initially discovered as a mechanism promoted by exogenous transcripts, such as viral RNAs (Cazalla et al, 2010; Marcinowski et al, 2012), TDMD was later found to occur with endogenous coding and non-coding transcripts, from *Drosophila* to human cells, leading to profound biological consequences (Bitetti et al, 2018; Ghini et al, 2018; Kingston et al, 2022; Kleaveland et al, 2018; Li et al, 2021; Sheng et al, 2023). Mechanistically, TDMD occurs since an atypical target:miRNA structure is formed, and it is followed by a conformational change in the AGO:miRNA complex (Sheu-Gruttadauria et al, 2019). This structure is recognized by the protein adaptor ZSWIM8 and the cullin-RING E3 ubiquitin ligase complex, promoting polyubiquitination of AGO, its proteasomal degradation and the decay of the miRNA by cytoplasmic nucleases (Han et al, 2020; Shi et al, 2020). The role of ZSWIM8 as a critical mediator of TDMD provided a valuable tool for the dissection of TDMD in different biological contexts and for the identification of potential TDMD substrates (i.e., those miRNAs which are stabilized upon ZSWIM8 perturbation). So far, 34 miRNAs have been identified in mammalian cells upon knock-out of ZSWIM8 (Han et al, 2020; Li et al, 2021; Shi et al, 2020); 21 in *Drosophila* upon loss of Dorado (Dora) (Kingston et al, 2022; Shi et al, 2020), and 10 in *C. elegans* using a null mutation of *ebax-1*(Shi et al, 2020). Loss of ZSWIM8 is detrimental for embryonal development in mouse, with ~50 miRNAs found as potential TDMD substrates across multiple tissues in two independent studies (Jones et al, 2023; Shi et al, 2023). Taken together, these findings support the idea that TDMD is a key mechanism in vertebrates with an impact on developmental regulation and a broad landscape of regulated miRNAs. Cancer cells frequently hijack cellular mechanisms to promote aberrant tumor cell behavior (Hanahan and Weinberg, 2011). In tumors, miRNAs have been described as either oncogenes or tumor suppressors and, therefore, are considered promising therapeutic targets (Lujambio and Lowe, 2012). We previously conducted an in silico analysis using a computational pipeline, named TDMD*finder*, that identifies potential TDMD interactions in the human and mouse transcriptome (Simeone et al, 2022). Multiomics analysis with The Cancer Genome Atlas (TCGA) datasets suggested the involvement of several TDMD pairs in human cancer, proposing TDMD as a potential oncogenic mechanism (Simeone et al, 2022). Based on these premises, a possible involvement of TDMD in human cancer is plausible. In this study, we exploited the genetic perturbation of ZSWIM8 as a tool to systematically map endogenous TDMD substrates across a panel of human breast cancer cell models. Combining ZSWIM8 perturbation with transcriptome-wide isolation of AGO-bound miRNA targets, we could highlight the impact of TDMD on the expression levels and the activity of miRNAs in cancer cells. Additionally, we explored the functional consequences of TDMD and observed that the regulation of miR-29b-3p by NREP contributes to transcriptional mechanisms characterizing cancer subpopulations and tumor heterogeneity. Finally, we obtained evidence of a noncanonical TDMD mechanism that is ZSWIM8- and proteasome-independent, which is active in cancer cells and regulates the levels of the tumor suppressor miR-30c-5p. Our findings provide evidence for a direct role of TDMD in cancer biology and underscore the need to further investigate regulated miRNA decay as a critical layer of post-transcriptional control in tumor development and progression.

## Results

### Identification of TDMD miRNA substrates in breast cancer by ZSWIM8 depletion

**Figure 1:**
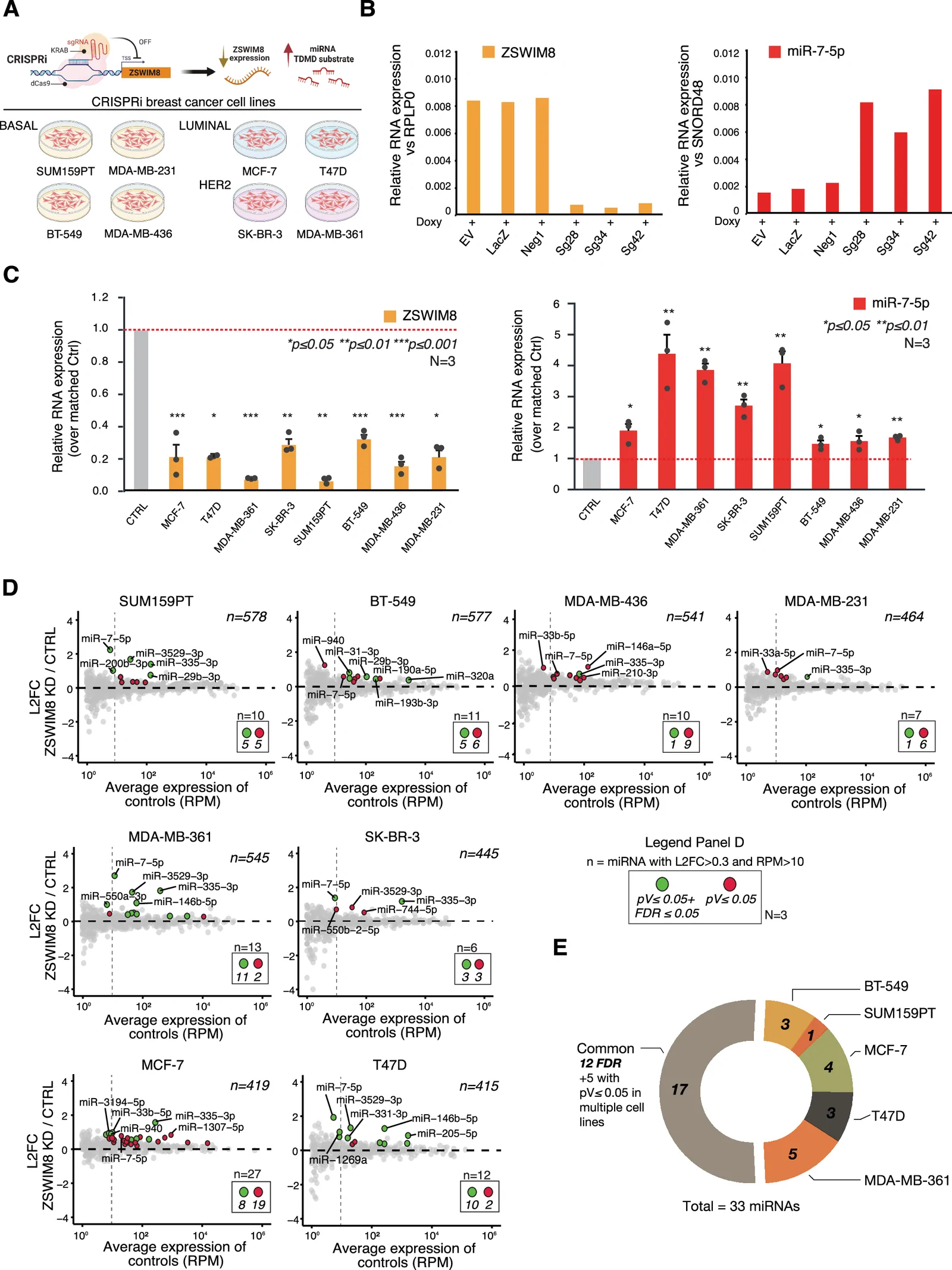
Identification of TDMD miRNA substrates in human breast cancer cells. (**A**) Scheme summarizing the dCas9-KRAB system used to engineer the CRISPRi breast cancer (BC) cell lines used in this manuscript. The enzymatically dead Cas9 conjugated with a KRAB repressive domain is directed to the promoter of the targeted gene through the expression of single guide RNAs (sgRNAs). The BC cell lines used belong to the luminal, basal, and HER2 amplified (HER2+) subtype. (**B**) *ZSWIM8* and miR-7-5p expression was measured upon inducible ZSWIM8 KD in SUM159PT. Shown results of RT-qPCR performed upon 0.5 μg/ml Doxycycline (+) for seven cell doublings. Relative expression of *ZSWIM8* and miR-7-5p (three different control sgRNAs and three sgRNAs targeting ZSWIM8) are normalized over *RPLP0* and *SNORD48*, respectively. (**C**) RT-qPCR of *ZSWIM8* and miR-7-5p expression measured upon ZSWIM8 KD in all the CRISPRi BC cell lines, as in panel (**B**). Shown is the average of the results with the three different sgRNAs (*N* = 3; ± SEM), normalized over *RPLP0* (ZSWIM8) and *SNORD48* (miR-7-5p) and over the corresponding control sgRNAs (ctrl, shown as reference with a gray bar). Statistical analysis was performed using Student’s *t*-test; *p ≤ 0.05, **p ≤ 0.01, ***p ≤ 0.001. (**D**) Small-RNA-Seq results of the 8 BC cell lines calculated over three independent biological replicates (*N* = 3). For each cell line, the graphs show the fold change of miRNA levels assessed upon ZSWIM8 KD over the average expression of the controls. The x-axis reports the average miRNA expression (RPM) across control samples. Significantly upregulated miRNAs were identified using an L2FC >0.3 and a *p* < 0.05 (Student’s *t*-test); miRNA candidates were further classified according to the FDR calculated using EdgeR and DESeq2 (<0.05). The criteria are summarized in the figure, along with the color code. The number of total detected miRNAs (*n*) is reported in each graph. (**E**) Donut chart summarizing the 33 miRNAs significantly upregulated upon ZSWIM8 KD, distinguished according to the cell line or significant in multiple BC cell lines (“Common”). Five miRNAs in the “Common” set (miR-33a-5p, miR-106-3p, miR-26a-2-3p, miR-26b-5p, and miR-1180-3p) were considered for further evaluation, even if not reaching the FDR threshold. Source data are available online for this figure.

To reveal TDMD-regulated miRNAs (“substrates” hereafter) in breast cancer, we sought to deplete ZSWIM8 expression in a panel of cell lines, comprising Luminal- (MCF-7, T47D), HER2-positive (SK-BR-3, MDA-MB-361) and Basal- subtypes (SUM159PT, MDA-MB-231, BT-549, MDA-MB-436) (Fig. 1A). Specifically, we exploited the CRISPR interference (CRISPRi) technology, where the catalytically dead Cas9 nuclease (dCas9) is fused to the Krüppel-associated-box transcriptional repressor protein (KRAB) (Gilbert et al, 2013). The fusion protein was expressed in a doxycycline-inducible fashion and directed to the transcription start site (TSS) of the target gene through selective single guide RNAs (sgRNAs). We verified dCas9-KRAB expression in all the breast cancer cell lines upon doxycycline treatment (Appendix Fig. S1A) and designed sgRNAs targeting the ZSWIM8 promoter (sgRNA28, sgRNA34, sgRNA42) and negative controls (Empty Vector, sgRNA LacZ and a non-targeting sgRNA, Neg1). Each of the ZSWIM8 targeting sgRNAs produced a robust knockdown of the target (>80% reduction of ZSWIM8 expression, Fig. 1B) and caused the accumulation of miR-7-5p—a previously reported TDMD substrate, although the extent of accumulation varied among the different cell lines (Han and Mendell, 2023; Kleaveland et al, 2018; Shi et al, 2020) (Fig. 1B,C). We measured miRNA levels in each cell line by small RNA-Seq, aiming at unveiling the landscape of potential TDMD substrates. (Fig. 1D). Across the eight cell lines, we defined a set of 63 candidate TDMD substrates, defined as those miRNAs found significantly upregulated upon ZSWIM8 KD (L2FC >0.3 and *p* value ≤0.05) and expressed above a threshold (≥10 RPM) in ZSWIM8-depleted cells (Fig. 1D,E; Dataset EV1). Among the 63 miRNAs, 28 displayed an FDR <0.05 by EdgeR and DESeq2 analysis, while five were supported in multiple cell lines, though not reaching statistical support by FDR (Fig. 1D,E; Dataset EV1). MiR-7-5p and miR-335-3p were significantly upregulated in most of the cell lines, a result which is in line with the multi-tissue regulation observed in *Zswim8*^−/−^mice (Jones et al, 2023; Shi et al, 2023). Conversely, most of the significantly upregulated miRNAs were cell-type specific: we typically observed ~10 candidate substrates per cell type, with 17 of the 33 miRNAs (27%) being upregulated in multiple cell lines (Fig. 1E), reflecting differences in miRNA expression and/or trigger availability across cell types (Appendix Fig. S1B). Among the 33 candidate substrates, the average level of accumulation upon TDMD impairment was relatively mild (average L2FC 0.79), similar to what was previously found (Han et al, 2020; Shi et al, 2020). Notably, we also found miRNAs significantly downregulated upon ZSWIM8 KD, which cannot be explained as a direct effect of TDMD impairment (Appendix Fig. S1C,D), but it is rather suggesting that secondary events are produced by ZSWIM8 perturbation. Therefore, we set up a strategy to provide additional evidence on the post-transcriptional regulation of the candidate miRNA substrates, which is described in the following paragraph.

### A set of 19 high-confidence TDMD substrates are found in breast cancer cells

**Figure 2:**
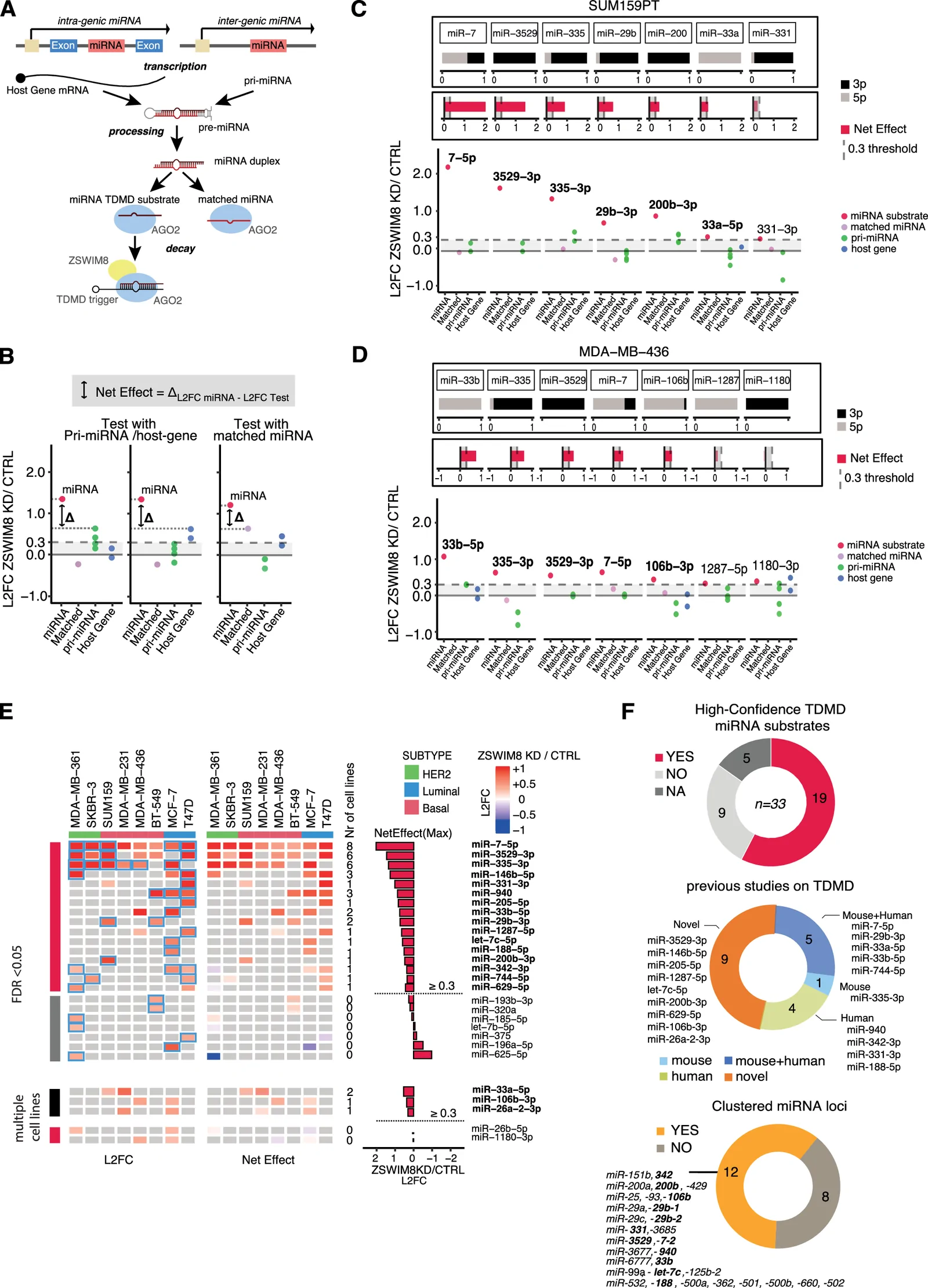
Identification of high-confidence TDMD substrates in human breast cancer cells. (**A**) Schematic representation of the steps of miRNA biogenesis. Primary miRNA transcripts (pri-miRNA) can originate either from intergenic miRNAs or from host genes. For a given miRNA substrate, the “matched strand” miRNA refers to the opposite strand from the same miRNA duplex. (**B**) Schematic representation of the strategy used to calculate the TDMD Net Effect, which represents the regulation of miRNAs upon ZSWIM8 KD after excluding indirect transcriptional effects measured by a Test assay (the primary transcript, the host–gene or the “matched strand” miRNA). Normalization was performed using the Test assay with the highest positive L2FC, as shown in the examples. (**C**,** D**) Regulations of the candidate miRNA substrates in SUM159PT (**C**) and MDA-MB-436 (**D**). For each miRNA is reported the distribution of reads from the 5p/3p strands, the TDMD net effect (red bar) and the values (L2FC values upon ZSWIM8 KD) of miRNA substrate (red dots) and matched miRNA (pink) obtained by sRNA-Seq analysis (*N* = 3, same data as in Fig. 1D), together with their pri-miRNAs (green), and host genes (blue dots) obtained by RT-qPCR (*N* = 3; analysis performed on the matched samples used in sRNA-seq), normalized with either *RPLP0* or *GADPH* (two or more dots are shown, considering the two normalizations and different pair of primers used). A dashed bar indicates the threshold for the “TDMD net effect” (≥0.3 L2FC). Missing dots for matched miRNAs or pri-miRNAs correspond to species that were not detectable under the experimental conditions. (**E**) Cellplot displaying for each miRNA the regulation as L2FC (ZSWIM8 KD/CTRL, on the left; FDR support is indicated by blue boxes) and the TDMD net effects (on the right). A red bar shows the maximum of the net effects across the eight BC cell lines. Dashed gray lines mark thresholds for net effects equal to or greater than 0.3 L2FC. (**F**) Upper, donut chart showing the high confidence (HC) set of TDMD substrates defined by having a TDMD net effect greater than 0.3 L2FC in at least one cell line (19 miRNAs). “NA” refers to miRNAs that could not be tested for the net effect due to an undetectable matched strand and pri-miRNA. Mid, donut chart showing the comparison with previous studies in human and mouse, using a similar strategy (i.e., ZSWIM8 loss of function). Lower, pie chart showing the organization into clusters of the set of 19 HC miRNA substrates (in bold). Note that miR-29b-3p is transcribed from two clusters (i.e., the 19 HC miRNA substrates are organized in 20 loci). Source data are available online for this figure.

**Figure EV1:**
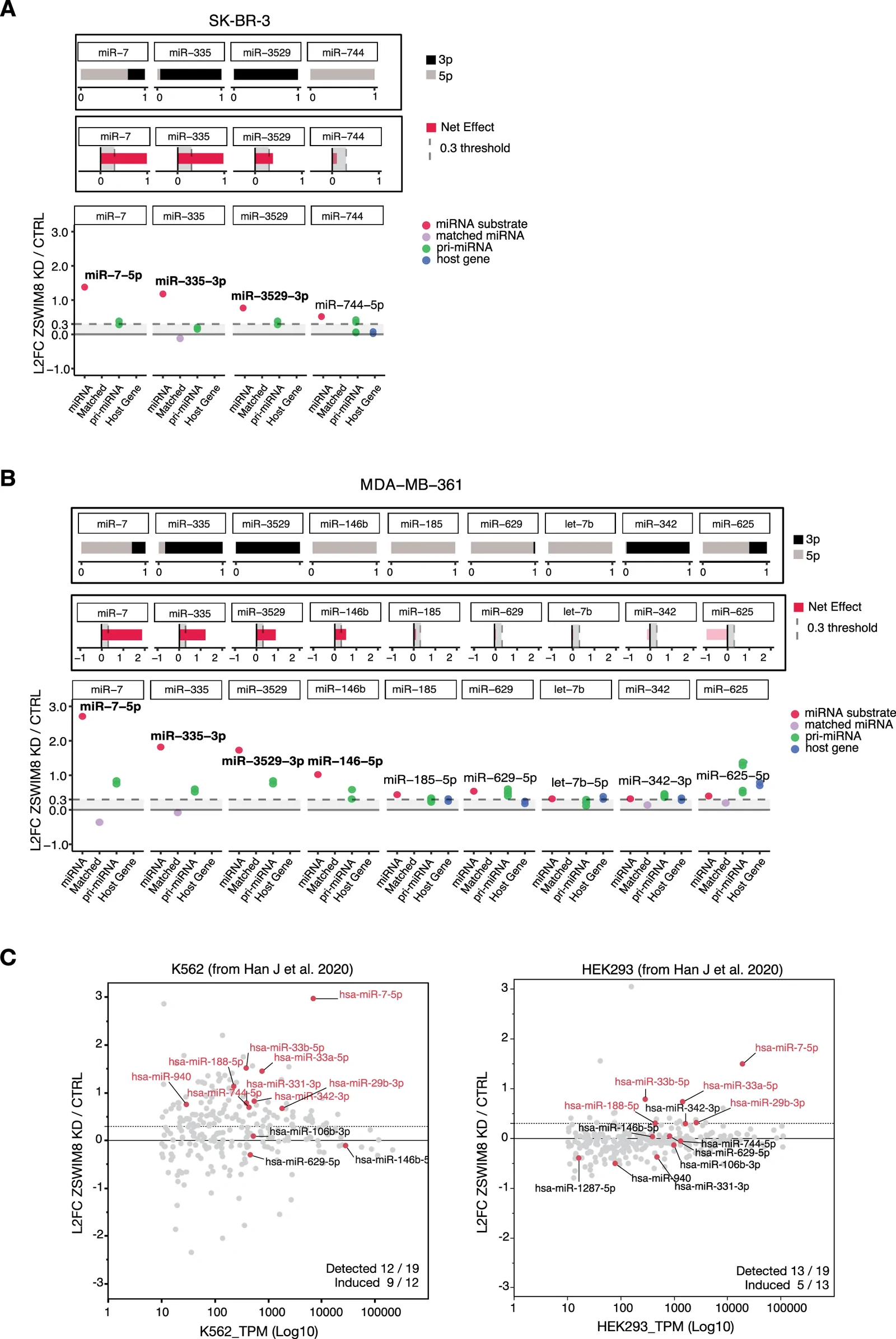
Identification of high-confidence TDMD miRNA substrates. (**A**, **B**) For each miRNA found as upregulated upon ZSWIM8 KD (see Fig. 2E) is reported the distribution of reads from the 5p/3p strands, the TDMD net effect (red bar) and the values (L2FC values upon ZSWIM8-KD) of miRNA substrate (red dots) and matched miRNA (pink) obtained by sRNA-Seq analysis, together with their pri-miRNAs (green), and host genes (blue dots) obtained by RT-qPCR, normalized with either RPLP0 or GADPH (two or more dots are shown, considering the two normalizations and different pair of primers used). A dashed bar indicates the threshold for the “TDMD net effect” (≥0.3 L2FC). *N* = 3. (**C**) Regulation of TDMD substrates identified in this study in other human cell lines (left K562; right HEK293; data from (Han et al, 2020)). The graphs show the fold change of miRNA levels assessed upon ZSWIM8 KD over the average expression of the controls. The red dots represent the breast cancer substrates that were detected (>10 TPM) in the corresponding cell line; miRNAs showing an L2FC >0.3 were considered as “induced” and are bolded.

TDMD is a post-transcriptional mechanism that acts on mature miRNAs in a sequence-specific manner. Therefore, it is supposed to affect neither the transcription nor the processing of miRNAs, which can be checked by measuring the abundance of miRNA precursors (pri-miRNA) and the expression of the cognate miRNAs on the complementary strand that is produced by processing of the same precursor molecule (hereafter defined as “matched strand”, Fig. 2A). We focused on the 33 candidate miRNA substrates (i.e., 28 with FDR support and five found in multiple cell lines) and evaluated the levels of pri-miRNAs by RT-qPCR and the expression of matched strand miRNAs by small RNA-seq upon ZSWIM8 perturbation (Figs. 2B–D an EV1A,B; Appendix Fig. S2). For intronic miRNAs, we monitored the levels of the host genes by RT-qPCR. We calculated a “TDMD net effect”, which is obtained by the normalization of the regulation observed upon ZSWIM8 perturbation (fold change of the candidate substrate) over the regulation of a test assay —fold change of the precursor, host gene or matched strand—using the test assay with the highest positive regulation (Fig. 2B). This strategy serves to minimize the number of false positive hits and increase the stringency of substrate identification by excluding transcriptionally driven effects. In total, we obtained 19 High-Confidence TDMD substrates (HC set) that showed a TDMD net effect ≥0.3 L2FC in at least one cell model (Figs. 2E and EV1A,B; Appendix Fig. S2). For example, in the case of SUM159PT and MDA-MB-436, six substrates passed the filtering (Fig. 2C,D). The HC set contains two miRNAs previously characterized as endogenous TDMD substrates —miR-7-5p and miR-29b-3p—listed among the top ten when ranking for TDMD net effect (Fig. 2E). Interestingly, half of the HC substrates (11 out of 20, 55%) are members of the so-called miRNA clusters (Fig. 2F), which contain multiple co-transcribed miRNAs. Notably, the co-transcribed miRNAs did not show a similar upregulation upon ZSWIM8 KD. For instance, in SUM159PT, miR-29b-3p resulted as upregulated, but miR-29a-3p and miR-29c-3p, that are co-transcribed from the same genomic locus, did not, suggesting that TDMD is providing a mechanism capable of uncoupling the expression of clustered miRNAs, as previously hypothesized (Jones et al, 2023; Shi et al, 2023; Simeone et al, 2022). In conclusion, we identified 19 HC substrates from eight breast cancer cell lines. Most of these miRNAs are found in a single cell model, with only eight (miR-7-5p, miR-3529-3p, miR-335-3p, miR-146b-5p, miR-940, miR-33b-5p, miR-33a-5p, and miR-29b-3p) being regulated in multiple breast cancer cell lines. The HC set comprises both previously reported and new TDMD-regulated miRNAs (Fig. 2F): six miRNAs were reported as ZSWIM8 sensitive during mouse development (miR-7-5p, miR-33a-5p, miR-33b-5p, miR-29b-3p, miR-744-5p and miR-335-3p) (Jones et al, 2023; Shi et al, 2023), while nine miRNAs were previously observed as potential substrates in cancer cell lines from different tissues, namely K562 and HEK293T (Fig. EV1C) (Han et al, 2020), with miR-7-5p, miR-29b-3p, miR-33a-5p, miR-33b-5p, and miR-744-5p being found in all contexts (mouse and human). In addition, our analysis captures nine novel substrates. These miRNAs are likely specific TDMD substrates of the breast context, as a consequence of a tissue-specific expression of either the trigger or the miRNA (e.g., miR-205-5p, which is expressed only in basal cells, as previously observed (Gregory et al, 2008).

### TDMD affects miRNA target occupancy in cancer cells

**Figure 3:**
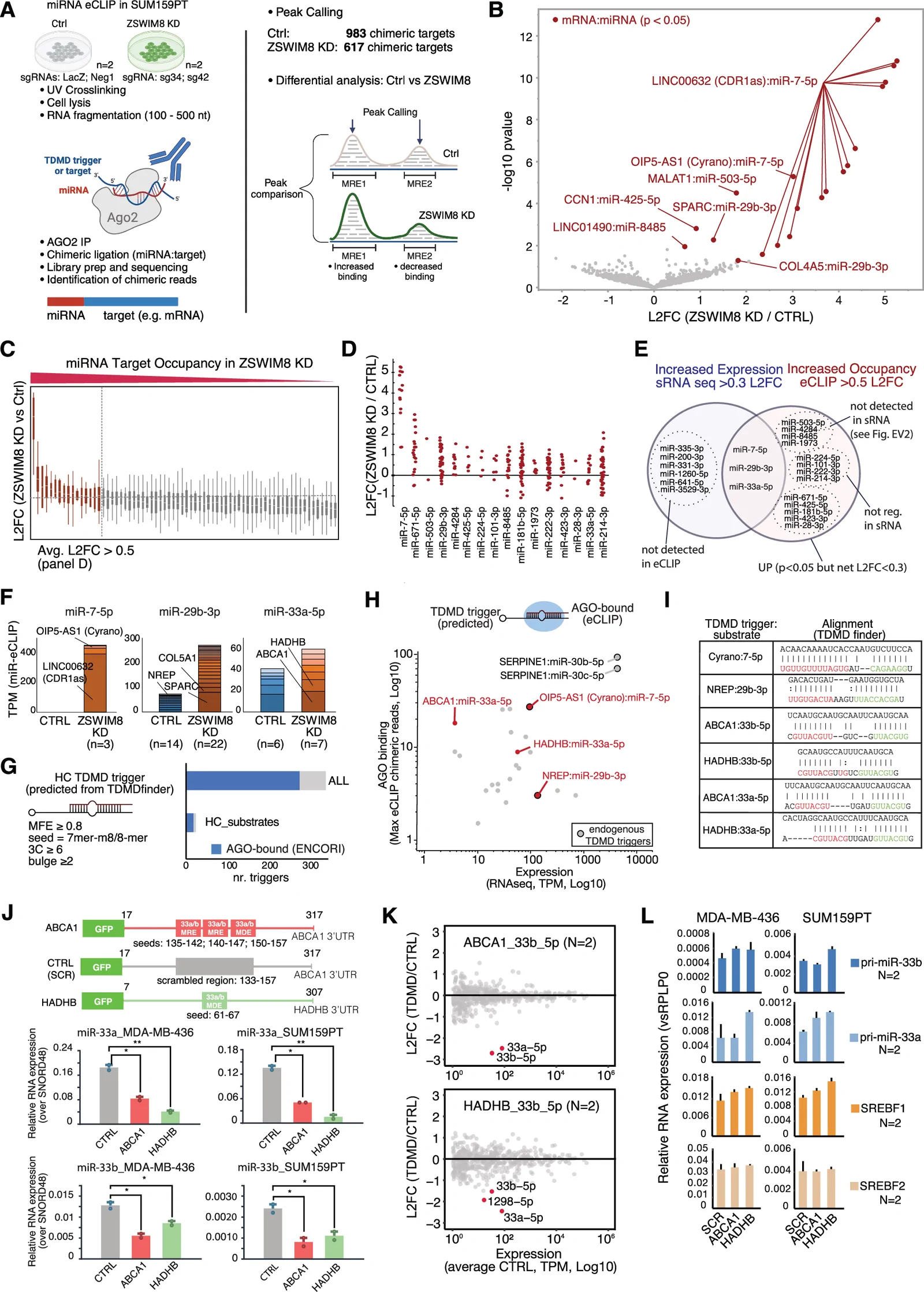
Identification and validation of TDMD triggers in BC cells. (**A**) Schematics of the miR-eCLIP experiment in SUM159PT cells. Cells were infected with lentivirus expressing two control sgRNAs (LacZ and Neg1 as in Fig. 1B) and two sgRNAs targeting ZSWIM8 (sg34 and sg42, as in Fig. 1B) and then treated with 1 μg/mL doxycycline, to activate the dCas9-KRAB transgene, for 10 days before performing miR-eCLIP (see Methods). Plots (**B**–**H**) show results from two biological replicates. (**B**) The volcano plot shows the L2FC (ZSWIM8 KD/CTRL) versus the significance (by Wald Test) of all chimeric peaks, with genes enriched in perturbed cells colored in red. Multiple entries for the same target:miRNA pair imply the presence of different miRNA responsive elements. (**C**) Box plot reporting the differential miRNA target occupancy (ZSWIM8 KD/CTRL) from miR-eCLIP in perturbed and control cells. The miRNAs with >0.5 L2FC are shown in red. (**D**) Differential binding of individual chimeric peaks are reported for the 16 miRNAs showing increased (>0.5 L2FC) occupancy upon ZSWIM8 depletion. (**E**) Venn diagram of the miRNAs showing increased expression (by sRNA-seq) or increased occupancy (by miR-eCLIP) after ZSWIM8 depletion. Thresholds (log2FC >0.3 for expression; >0.5 for occupancy) were selected based on prior TDMD studies and tailored to reflect the typically modest, but biologically meaningful, impact of TDMD in non-neuronal cells. (**F**) Stacked bar chart showing the normalized counts of chimeric reads for miR-7-5p, miR-33a/b, and miR-29b-3p. Putative TDMD triggers (OIP5-AS1/Cyrano, NREP, ABCA1, HADHB) and top binders that do not fulfill TDMD structural criteria (LINC00632/CDR1as, COL5A1; SPARC) are labeled. (**G**) Pairs of TDMD triggers and miRNA substrates were predicted using the *TDMDfinder* tool (see Methods). A schematic representation illustrates the canonical binding features required for MDE selection. The stacked bar chart reports the number of predicted triggers for all expressed miRNAs or for TDMD substrates. The miRNA:target pairs supported by AGO-CLIP experiments (ENCORI database) are in blue. (**H**) Plots reporting the expression level by RNA-seq (TPM, Transcripts per Million) and AGO-binding by miR-eCLIP (normalized peak reads) of candidate triggers in SUM159PT. Highlighted in red are the putative triggers for miR-7-5p, miR-33a/b and miR-29b-3p. Known endogenous TDMD triggers are annotated. (**I**) Alignment of TDMD triggers and miRNA substrates, as predicted by *TDMDfinder* tool. (**J**) Expression of miR-33b-5p and miR-33a-5p was measured by RT-qPCR in MDA-MB-436 and SUM159PT cells upon overexpression of the putative degradation elements of candidate TDMD triggers (7 days post infection). Top: scheme of the constructs used for the TDMD assay. Bottom: bar charts with miR-33a-5p and miR-33b-5p relative expression (*N* = 2; average ± SEM) normalized over *SNORD48*. (**K**) sRNA-Seq analysis showing miRNAs expression upon overexpression of ABCA1 (top) and HADHB (bottom) in MDA-MB-436 cells. The graph shows the average L2FC of miRNA levels calculated over control samples. The red dots highlight miRNAs with an L2FC ≤−1.5 and an expression of ≥10 RPM; *N* = 2 biological replicates. (**L**) Pri-miRNAs (pri-miR-33b and pri-miR-33a) and host genes (*SREBF2* and *SREBF1*) were measured by RT-qPCR upon overexpression of the 3’UTR of ABCA1 and HADHB in MDA-MB-436 (left) and SUM159PT (right) cell lines. The bar chart shows the relative RNA expression (*N* = 2; average ± SEM) using *RPLP0* as reference. Source data are available online for this figure.

**Figure EV2:**
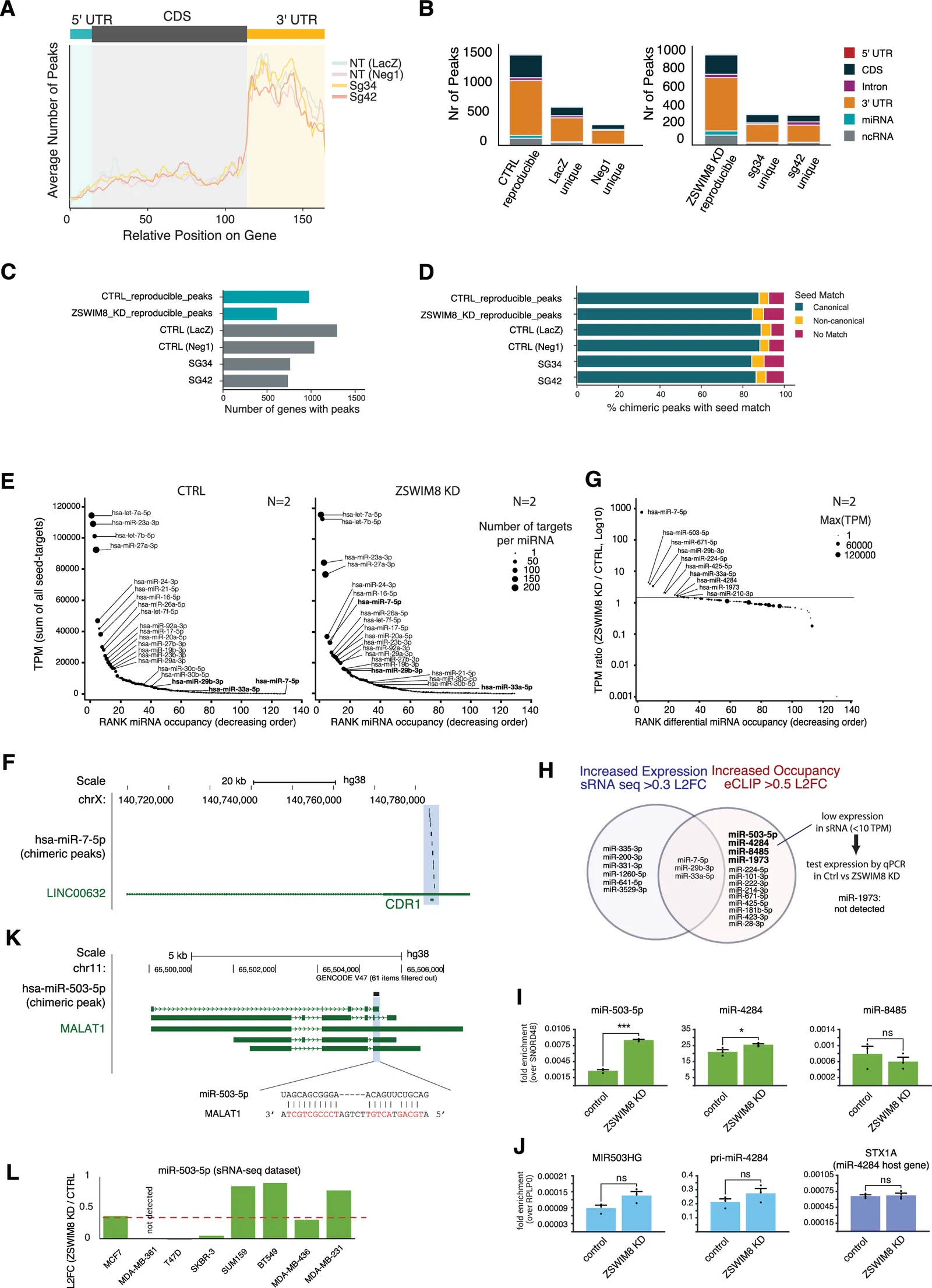
miR-eCLIP identifies endogenous miRNA:target interactions. (**A**) Metagene plot showing the average number of AGO2 peaks along the 5’UTR, CDS, and 3’UTR for peaks called in each sample (control sgRNA118 and 119; ZSWIM8 KD sgRNA34 and 42). In order to create the metagene, peaks were downsampled to match the peak set with the smallest number to account for differences in the number of significant peaks and gene lengths were normalized. (**B**) The AGO2 peak upset plot shows the feature distribution for reproducible peaks, as well as unique peaks for each replicate, in control (left) or ZSWIM KD cells (right). (**C**) Number of genes with chimeric peaks in each sample or reproducible across replicates (see Methods for the identification of reproducible peaks). (**D**) Proportion of seed match among chimeric peaks. Canonical sites are 6mer, 7mer-1A, 7mer-m8, and 8mer; noncanonical sites are seed-less: 3’ compensatory or centered. (**E**) Bubble plot showing miRNA target occupancy in control (left) or ZSWIM8 KD (right) cells, as estimated from the cumulative count of all targets found in chimeric reads (expressed in transcript per million, TPM). MiRNAs are ranked from high to low occupancy. The dimension of the bubbles is proportional to the number of targets. (**F**) Chimeric peaks for hsa-miR-7-5p and LINC00632. All the peaks localize on the CDR1as region. (**G**) Bubble plot reporting the differential miRNA activity (ZSWIM8 KD vs Ctrl), calculated by a ratio of the values reported in panel E. The dimension of the bubble is proportional to the max miRNA target occupancy observed either in control or ZSWIM8 KD cells. (**H**–**J**) MiRNAs with increased target occupancy by eCLIP and low expression by sRNA-seq were re-analyzed by RT-qPCR in control and ZSWIM8 KD cells. MiR-1973 was not detected even by RT-qPCR. (**I**) Bar charts reporting the expression levels by RT-qPCR of mature miRNAs measured upon 0.5 μg/ml doxycycline (+) for seven cell doublings on cells infected with controls and ZSWIM8 targeting sgRNAs (average and SEM; *N* = 3). Data were normalized over *SNORD48*. Statistical analysis with the Student’s *t*-test; *p ≤ 0.05, ***p ≤ 0.001. (**J**) Bar charts showing the expression levels of pri-miRNAs and host genes by RT-qPCR, as in (**I**). Data were normalized over RPLP0 (average and SEM; *N* = 3). Statistical analysis with the Student’s *t*-test. (**K**) Chimeric peak for hsa-miR-503-5p and MALAT1. The peak contains a region of MALAT1 displaying an extended complementarity to miR-503-5p, as shown. (**L**) sRNA-seq data for miR-503-5p were re-analyzed in all BC samples (as in Fig. 1), including those with miR-503-5p expression <10 TPMs, and are reported as average L2FC between control and ZSWIM8 KD cells (see also Dataset EV1).

The magnitude of miRNA regulation upon ZSWIM8 KD was generally modest for most HC miRNAs (average L2FC = 0.97, net-effect L2FC = 0.79, Dataset EV1), which is typical of TDMD-mediated effects. Previous studies have shown that TDMD can affect miRNA activity and lead to measurable phenotypic outcomes, even in cases where fold changes are small—especially when the substrate is near its functional threshold (Ghini et al, 2018; Li et al, 2021). To better capture this effect, we directly assessed the binding of individual miRNAs to their targets within the miRISC complex. Unlike alternative techniques that provide indirect methods for mapping miRNA targets, miR-eCLIP detects direct miRNA targets using AGO2 immunoprecipitation, RNA–RNA ligation, and high-throughput sequencing (Manakov et al, 2022; Van Nostrand et al, 2016), producing a transcriptome-wide map of the endogenous miRNA:target interactions (Fig. 3A) that can be used to evaluate miRNA activity and, at the same time, to identify possible TDMD triggers. In the absence of ZSWIM8, triggers are predicted to bind the miRNAs without producing degradation (Li et al, 2021). We generated miR-eCLIP datasets from wild-type and ZSWIM8 KD SUM159PT cells. We selected the SUM159PT cell line for miR-eCLIP for two main reasons: (i) it was the only one showing TDMD on both miR-29b-3p and miR-33a/b, tumor-suppressor miRNAs previously found to be ZSWIM8 sensitive and predicted as TDMD-regulated in multiple cancer types (Simeone et al, 2022); (ii) SUM159PT is a well-characterized TNBC model that harbors a subpopulation of cells primed to tumor initiation, as shown in our recent work (Nadalin et al, 2024), thus allowing the investigation of TDMD in a highly plastic cancer model. We obtained an eCLIP dataset characterized by: (i) an enrichment of peaks in the 3’UTR (Fig. EV2A,B), (ii) reproducible peaks across replicates (Fig. EV2B–D), (iii) a high fraction (>85%) of chimeric peaks containing a canonical seed match (Fig. EV2D). In total, we identified 983 and 617 reproducible chimeric genes in control and ZSWIM8 perturbed cells, respectively, related to 129 distinct miRNAs (Fig. EV2E; Dataset EV2). Differential analysis of chimeric peaks comparing ZSWIM8 KD vs. CTRL cells highlighted enrichment for specific targets of miR-7 (OIP5-AS1/Cyrano and LINC00632/CDR1as, Fig. 3B), in line with the stabilization of miR-7-5p levels that we observed by RT-qPCR and small RNA-seq (see Figs. 1C and 2C). The increased interaction with the non-coding RNA Cyrano confirms its role as the endogenous TDMD trigger for miR-7 also in breast cancer cells. LINC00632 is the precursor of CDR1as, a circular RNA displaying a massive number of binding sites and acting as a critical miR-7 target (Piwecka et al, 2017). Of note, all miR-7 chimeric peaks were localized to the CDR1as exon (Fig. EV2F). This finding suggests that miR-7 target occupancy is highly restricted in breast cancer cells by different mechanisms involving two ncRNAs, Cyrano and CDR1as, with very limited interactions with other targets, in line with the model previously described in the brain (Kleaveland et al, 2018). We extended the analysis on all AGO2-bound miRNAs and calculated a differential miRNA target occupancy, as the average change in the binding to each target between perturbed and control cells (Fig. 3A,C). We identified 16 miRNAs with significantly increased target occupancy in ZSWIM8 KD cells (average L2FC >0.5, Fig. 3C,D). Similar results were obtained using a different strategy to calculate miRNA target occupancy (Fig. EV2G; see Methods). Three miRNAs (miR-7-5p, miR-29b-3p, and miR-33a-5p) were found in common with the substrates found previously (Fig. 3E) and were prioritized as high-confidence TDMD substrates based on the concordance between increased miRNA expression (sRNA-seq) and increased target occupancy (miR-eCLIP) upon ZSWIM8 depletion. Differences in sensitivity and thresholds between sRNA-seq and miR-eCLIP may account for the limited overlap in identified TDMD substrates (Fig. 3E). To support this contention, we validated by qPCR a subset of miRNAs classified as low-expressed in sRNA-seq (Fig. EV2H,I) and found that miR-503-5p and miR-4284 were upregulated upon ZSWIM8 KD in a post-transcriptional manner (Fig. EV2I,J). Interestingly, we also observed increased miR-503-5p binding to MALAT1 in ZSWIM8 KD cells, as revealed by miR-eCLIP (Fig. 3B), with chimeric reads mapping to a region of MALAT1 previously shown to bind miR-503-5p and suppress its expression and activity (Yan et al, 2017) (Fig. EV2K). A re-analysis of sRNA-seq data further confirmed the induction of miR-503-5p in multiple cell lines upon ZSWIM8 KD (Fig. EV2L), although future studies will be needed to fully establish the role of TDMD in regulating this miRNA (see Discussion).

We focused on the target repertoire mapped by miR-eCLIP, looking for miRNA targets and putative TDMD triggers for the three shared miRNAs (Fig. 3F). We took advantage of the TDMD*finder* pipeline that predicts putative TDMD interactions by in silico analysis (Simeone et al, 2022) and identified candidate triggers for the miRNA substrates (Fig. 3G; Dataset EV2). Besides Cyrano:miR-7-5p, we identified *NREP* as a potential trigger for miR-29b-3p, and *ABCA1* and *HADHB* for miR-33a-5p (Fig. 3H). Notably, highly bound targets, such as COL5A1 and SPARC, lacked the structural hallmarks of MDE (summarized in Fig. 3G and detailed in Dataset EV2) and therefore were not considered as potential triggers. *NREP* was previously described as an endogenous trigger, inducing TDMD of miR-29b-3p in mouse and zebrafish (Bitetti et al, 2018). To date, no triggers are reported for miR-33a-5p or its cognate miR-33b-5p (which we found as a substrate in other breast cancer cells); therefore, we sought to verify the candidate novel triggers for the two miRNAs. Both *ABCA1* and *HADHB* can pair with miR-33a-5p and miR-33b-5p, forming a structure compatible with TDMD, according to TDMD*finder* (Fig. 3I). We used a “TDMD assay” that we and others have previously developed to verify the ability of a transcript to promote the decay of the substrate upon exogenous expression of a GFP reporter containing in its 3’UTR the MDE of the candidate trigger (Simeone et al, 2022). In the “TDMD assay”, *ABCA1* and *HADHB* MDE triggered the degradation of either miR-33a-5p or miR-33b-5p in SUM159PT and MDA-MB-436 cells (Fig. 3J,K), as measured by RT-qPCR and sRNA-Seq, with specificity in the repression of the predicted miRNAs (Fig. 3K). We verified that the reduction in miRNA levels was not due to a transcriptional mechanism, as primary precursors (pri-miR-33) and host genes (*SREBF1* and *SREBF2*) were not affected (Fig. 3L). Taken together, these results show that TDMD can significantly affect both the levels and the target occupancy of miRNAs in cancer cells. In addition, we could identify by miR-eCLIP TDMD triggers directly bound to their substrate miRNAs. Two of them— *ABCA1* and *HADHB*—are novel triggers for miR-33 family: while we formally proved the direct interaction, the perturbation experiments presented here are based on overexpression. A conclusive demonstration of their role in TDMD would require manipulation of the endogenous MDE, which in this case is hindered by the feedback loops between miR-33 and these genes, all involved in cholesterol metabolism (see Discussion).

### *NREP* is the trigger of miR-29b-3p in mesenchymal breast cancer cells

**Figure 4:**
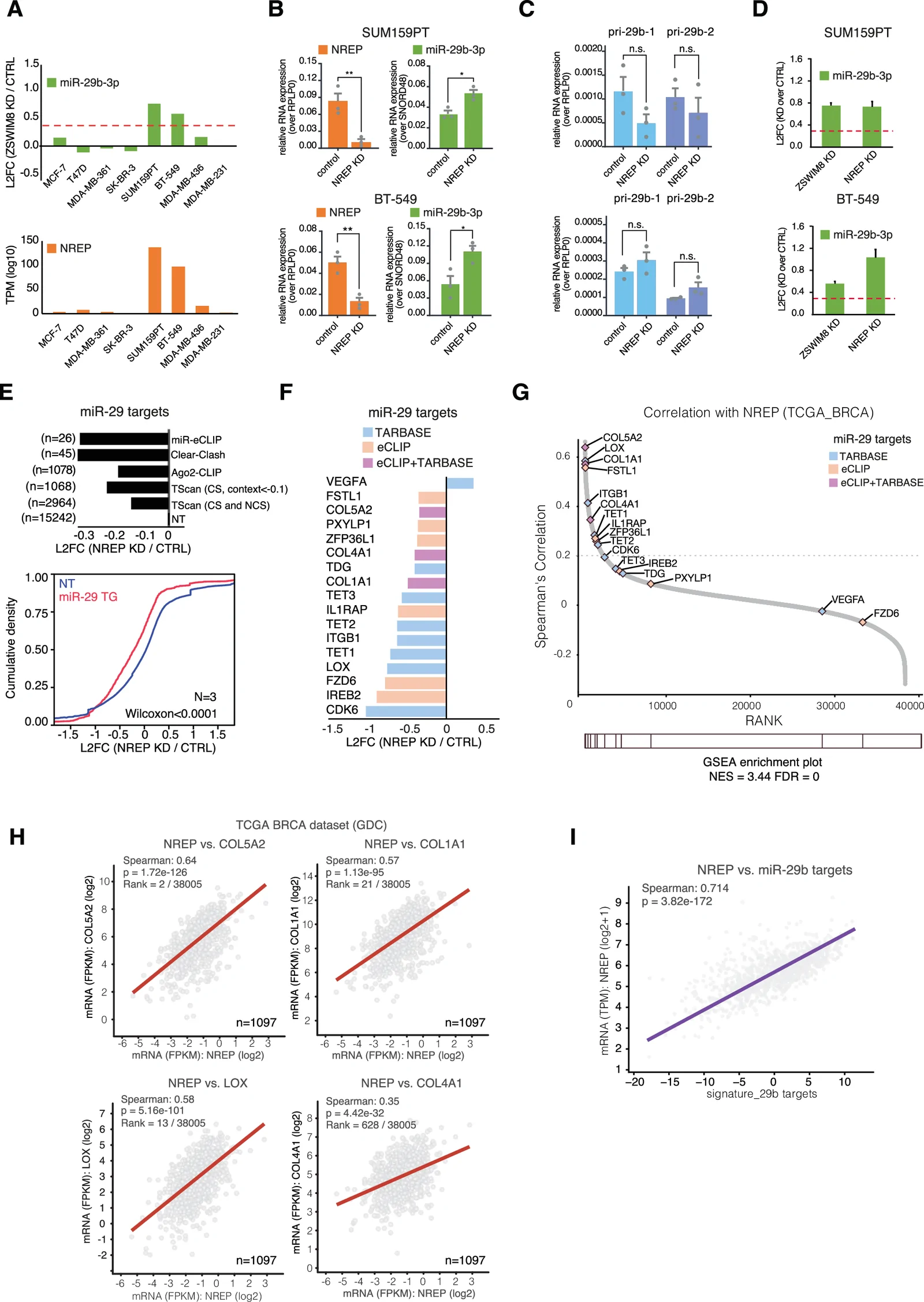
The NREP-miR-29b-3p axis in breast cancer cells and primary tumors. (**A**) Top: differential expression of miR-29b-3p in the BC cell lines, measured upon ZSWIM8 KD (data from sRNA-seq experiments, Dataset EV1). Bottom: expression of *NREP* in the same BC cell lines (TPM, by RNA sequencing). (**B**,** C**) CRISPRi was used to silence *NREP* using independent sgRNAs (*N* = 3) in SUM159PT (top) and BT-549 (bottom) cell lines. Shown are the expression levels by RT-qPCR of the targeted gene (*NREP*), the mature miRNAs (miR-29b-3p) and the pri-miRNAs (pri-29b1, pri-29b2), measured upon 0.5 μg/ml doxycycline (+) for seven cell doublings. The bar charts show the results of independent controls and NREP targeting sgRNAs (*N* = 3, average ± SEM). Data were normalized over *RPLP0* (for *NREP* and pri-miRNAs) and *SNORD48* (for miR-29b-3p). Statistical analysis with the Student’s *t*-test; *p ≤ 0.05, **p ≤ 0.01. (**D**) MiR-29b-3p expression in SUM159PT (top) and BT-549 (bottom) cells upon ZSWIM8 or *NREP* knock-down upon administration of 0.5 μg/ml Doxycycline (+) for seven doublings. The bar chart shows the L2FC (average ± SEM, *N* = 3) of the knock-down over the average of CTRLs. (**E**) RNA expression analysis of controls and *NREP* KD cells. Above, a bar chart reports the activity of miR-29-3p targets measured as median of the L2FC (NREP KD vs CTRL, *N* = 3), using different subsets of targets from: miR-eCLIP, CLEAR-CLASH, Ago2-CLIP (ENCORI dataset), and TargetScan, using high confidence conserved targets (CS with context score <−0.1) or all the conserved and non-conserved targets (CS + NCS). Below, a cumulative distribution function (CDF) of the L2FC of genes (NREP KD vs CTRL) is shown, comparing miR-29-3p predicted targets (TargetScan high-confidence) vs non-targets (NT). NT are expressed genes that in TargetScan are not annotated as miR-29-3p targets. Significance by the Wilcoxon test. (**F**) Differential gene expression of miR-29b-3p targets that are significantly regulated upon NREP KD. Targets include those from miR-eCLIP data (obtained in SUM159PT) or from TARBASE (v8.0, “experimentally supported”). (**G**) Spearman pair-wise correlation analysis between the expression level of NREP and all other genes (*n* = 38,005) was calculated in the TCGA BRCA dataset. Genes are ordered from the highest positive correlation with NREP. MiR-29b-3p targets, from panel (**F**), are highlighted and used to run a gene-set enrichment analysis (GSEA), which is reported as an enrichment plot (black lines correspond to targets; the NES >2 indicates strong positive correlation). (**H**) Spearman pair-wise correlation between the expression of NREP and four representative miR-29b-3p targets, as in (**G**). Shown in red is the regression line. (**I**) Spearman pair-wise correlation between NREP and the mean expression of all miR-29b-3p targets. Shown in violet is the regression line. Data analysis performed with GEPIA3 (Kang et al, 2025). Source data are available online for this figure.

**Figure EV3:**
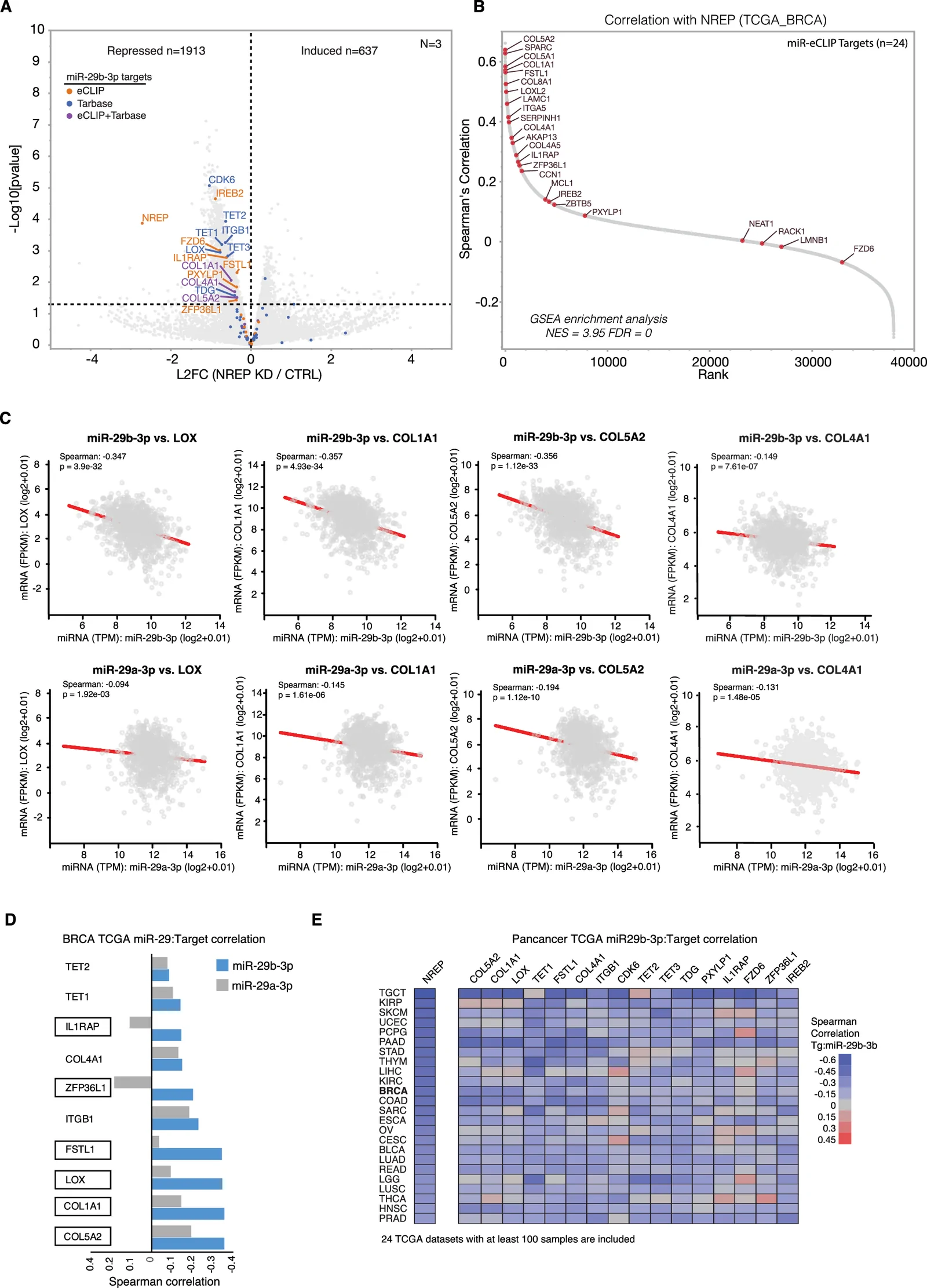
*NREP* perturbation increases miR-29b-3p activity. (**A**) Volcano plot showing differentially regulated genes (*p* value <0.05; NREP KD vs CTRL, *N* = 3; Deseq2) upon NREP perturbation in SUM159PT cells. Highlighted are miR-29b-3p targets identified by miR-eCLIP or from Tarbase. (**B**) Spearman pair-wise correlation analysis between the expression level of NREP and all other genes (*n* = 38,005) was calculated in the TCGA BRCA dataset. Genes are ranked according to the correlation with NREP (high to low). MiR-29b-3p targets, defined from miR-eCLIP chimeric peaks, are highlighted in red. The targets were used as a gene set to run gene set enrichment analysis (GSEA). The observed normalized enrichment score (NES = 3.95) indicates a strong positive association. (**C**) Top: Spearman pair-wise correlation analysis between the expression level of miR-29b-3p (x-axis, data in TPM) and selected target genes (y-axis, data in FPKM) calculated using the TCGA BRCA dataset. Bottom: the same analysis was performed with miR-29a-3p. (**D**) Bar chart summarizing the Spearman coefficient between miR-29b-3p (blue) or miR-29a-3p (gray) and *NREP*-dependent miR-29b-3p targets (i.e., top 10 from Fig. 4G). A box indicates genes showing stronger correlation with miR-29b-3p as compared to miR-29a-3p. (**E**) Heatmap showing the Spearman correlation of miR-29a-3p with *NREP*-dependent miR-29b-3p targets across 24 TCGA datasets (Breast cancer, BRCA, highlighted in bold). *NREP* is shown as a reference.

The regulation of miR-29b-3p by TDMD in breast cancer cells appeared particularly interesting. The 29b-3p:NREP TDMD pair has been previously found as the most significant predicted interaction in a pan-cancer analysis performed using the TCGA multiomic dataset (Simeone et al, 2022) (Dataset EV2). Further, NREP is emerging as a possible oncogene in TNBC (Ruan et al, 2024), whereas in breast cancer, miR-29b-3p behaves as a tumor suppressor (Chou et al, 2013). Our miR-eCLIP experiments pointed to *NREP* as the endogenous trigger for TDMD. Of the TDMDfinder predicted miR-29b-3p targets (NREP, ROBO1, and CBX6; Dataset EV3), only NREP was bound to miR-29b-3p on AGO2 by miR-eCLIP. Notably, the TDMD regulation observed on miR-29b-3p correlates well with the expression of *NREP* transcript, with two cancer cell lines of basal/TNBC subtype, SUM159PT and BT-549 cells, showing a very high expression of *NREP* and a significant stabilization effect of the miRNA upon ZSWIM8 depletion (Fig. 4A,B). We exploited CRISPRi to perturb directly *NREP* expression in SUM159PT and BT-549 cells and observed a significant increase of miR-29b-3p levels by RT-qPCR (Fig. 4B). This effect was not due to a transcriptional regulation, as expression of miRNA precursors was unaltered (pri-miR-29b1 and pri-miR-29b2, Fig. 4C). The extent of regulation of miR-29b-3p by *NREP* KD was similar to ZSWIM8 KD, suggesting that *NREP* is responsible for most, if not all, the TDMD effects on miR-29b-3p in these cell lines (Fig. 4D). We investigated the impact of *NREP* perturbation on miRNA activity by measuring the expression of targets by RNA sequencing. Predicted targets by TargetScan were significantly repressed upon *NREP* depletion (Fig. 4E; Dataset EV3). The repression was stronger for high-affinity MREs (i.e., conserved targets with high context scores, according to TargetScan). Significant repression of target was also found using Ago2-CLIP supported targets (from ENCORI), targets defined according to CLEAR-CLIP or CLASH experiments (Helwak et al, 2013; Moore et al, 2015) and miR-eCLIP targets (Fig. 4E). Specifically, the miR-eCLIP dataset was obtained in SUM159PT, the very same model where RNA-seq was performed upon *NREP* KD. Among the set of genes significantly regulated upon *NREP* transcript depletion (Fig. EV3A; Dataset EV3), we found nine in common with the miR-eCLIP set (*N* = 26) and 11 with a set of targets annotated in Tarbase (*N* = 53; nine of which were validated specifically in breast tissue, Dataset EV3), with three shared targets of collagen family (*COL1A1*, *COL4A1*, *COL5A2*, Fig. 4F). Almost all these targets were significantly repressed (Fig. 4F). Hence, we refer to this TDMD interaction, whereby NREP triggers miR-29b-3p degradation leading to derepression of the miRNA targets, as the NREP:miR-29b-3p axis. We analyzed if the NREP:miR-29b-3p axis could be recapitulated also in breast cancer patients, using the TCGA BRCA dataset (Cancer Genome Atlas N 2012). We found moderate (>0.2) or strong (>0.4) positive correlation between the expression of NREP and most miR-29b-3p targets, defined from miR-eCLIP (Fig. EV3B) or as Tarbase/miR-eCLIP targets repressed upon NREP-depletion in SUM159PT (Fig. 4G,H). Coherently, a combined score (i.e., mean expression of all miR-29b-3p targets) showed strong correlation with NREP (Spearman 0.714, Fig. 4I). While NREP positively correlated with miR-29b-3p targets, the opposite trend emerged with mature miR-29b-3p (Fig. EV3C). Importantly, miR-29a-3p, albeit co-transcribed, showed reduced correlation with the same targets (Fig. EV3C,D). Similar findings were recapitulated in other cancer types, suggesting that the NREP-miR-29b-3p axis might be conserved across solid tumors (Fig. EV3E).

### *TWIST1* regulates *NREP* in mesenchymal breast cancer cells

**Figure 5:**
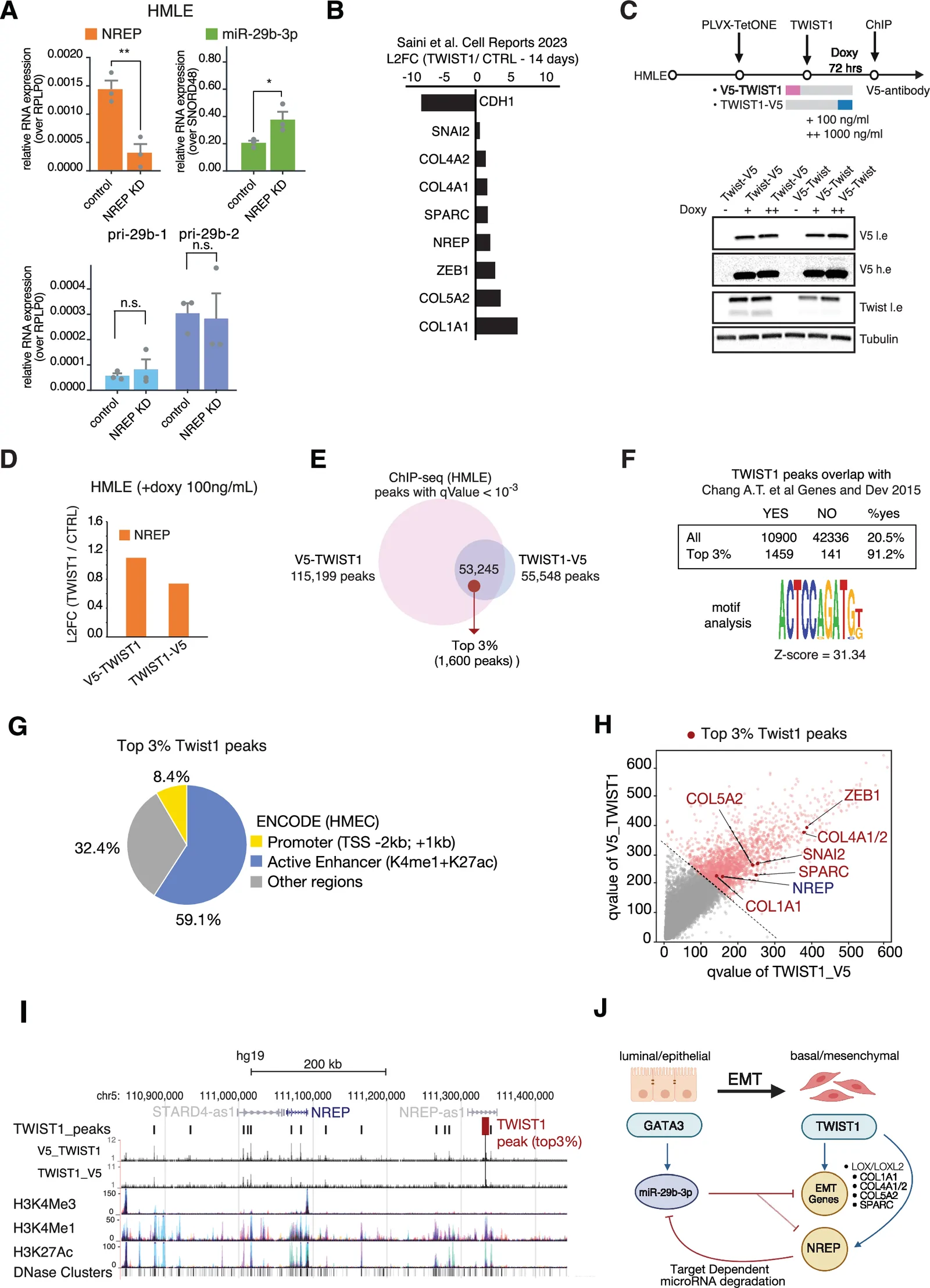
*TWIST1* regulates *NREP* in mesenchymal breast cancer cells. (**A**) CRISPRi was used to silence *NREP* using independent sgRNAs (*N* = 3) in the HMLE cell line. Data were reported as in Fig. 4C. (**B**) Differential gene expression upon TWIST1 overexpression in HMLE. Shown are miR-29b-3p targets that were found as significantly regulated in Saini et al (Saini et al, 2023), together with epithelial (*CDH1*) and mesenchymal (*SNAI2* and *ZEB1*) genes. (**C**) Top: scheme summarizing the strategy used for identifying TWIST1 target genes in HMLE cells. Bottom: Western blot from lysates of HMLE expressing TWIST1-V5 or V5-TWIST1 probed with antibodies anti-V5 (1:5000 Abcam, ab9116) or anti-TWIST1 (1:500, clone 2C1a; Santa Cruz). Samples were collected after 72 h of treatment with 100 ng/ml (+) or 1000 ng/ml (++) of doxycycline. (**D**) Bar chart reporting *NREP* differential expression measured by RT-qPCR upon acute TWIST1 activation (*N* = 2, one experiment with V5_TWIST1 and one with TWIST1_V5). (**E**) Total number of peaks (*q* value <0.001) from each set of ChIP-seq experiments and the overlap (Chi-square). (**F**) Comparison of the TWIST1 peaks identified in this paper with those from (Chang et al, 2015), identified by ChIP-seq using a TWIST1-ER construct. Shown is the overlap of all peaks (53,245) and of the Top 3% (1600). Top 3% peaks were subjected to motif analysis using *crawler* tool, retrieving as the top hit the TWIST1 motif shown. (**G**) Pie chart of the genomic position of the Top 3% peaks relative to gene promoters or enhancers (defined by peaks of H3K4me1 and H3K27ac, as from ENCODE ChIP-seq obtained in HMEC, GSM733705 and GSM733660). (**H**) Scatter plot displaying the q-value (−log10 scale) of ChIP-seq peaks found in V5-TWIST1 vs TWIST1-V5. A dashed line highlights the Top 3% high-confidence list. Representative target genes are highlighted, including *NREP*. (**I**) Genomic tracks (Genome Browser) of the *NREP* locus, reporting TWIST1 ChIP-seq peaks together with the histone marks from ENCODE. The peaks from the Top 3% list are highlighted in red. In gray are reported genes in the genomic window that were not expressed in the dataset. (**J**) Scheme summarizing a double negative feedback mechanism of EMT in breast cancer, involving transcriptional regulation and the TDMD mechanism. GATA3 and miR-29b-3p are active in luminal/epithelial cells, as opposed to TWIST1 and NREP in basal/mesenchymal breast cells. The *NREP*:miR:29b-3p pairing reinforces the EMT transition by reducing miR-29b-3p activity via TDMD. The targets reported in the scheme are downregulated upon NREP KD and further supported by miR-eCLIP or TARBASE. Source data are available online for this figure.

In breast, miR-29b-3p is highly expressed in luminal cells, where it acts in a double positive feedback loop with the transcriptional factor GATA3 and influences the expression of genes of the epithelial-to-mesenchymal transition (EMT), stemness and microenvironment (Chou et al, 2013). Importantly, the loss of miR-29b-3p in epithelial cells increases mesenchymal and pro-metastatic traits either in tumor cells or in a non-tumorigenic epithelial cell line (HMLE), targeting a network of genes involved in collagen remodeling (Chou et al, 2013). Based on these premises, we investigated if *NREP* was promoting TDMD on miR-29b-3p also in HMLE, a well-characterized model of epithelial-to-mesenchymal transition (EMT), containing epithelial cells and a small subpopulation of CD44^high^CD24^low^ cells with mesenchymal and stem-like properties (Mani et al, 2008). The knockdown of *NREP* by CRISPRi produced a significant increase in miR-29b-3p levels at a post-transcriptional level (Fig. 5A). TWIST1 transcription factor was previously shown to trigger the transition from CD44^low^CD24^high^ to CD44^high^CD24^low^ and promote EMT in HMLE (Mani et al, 2008). Therefore, we analyzed in detail the interplay between the NREP:miR-29b-3p axis and EMT plasticity. We interrogated an available gene expression dataset of HMLE stimulated with TWIST1 and observed induction of *NREP* and several miR-29b targets (Fig. 5B) (Saini et al, 2023). We confirmed by RT-qPCR that the *NREP* transcript was induced by acute TWIST1 expression, using a doxycycline-inducible TWIST1 (V5-TWIST1 and TWIST-V5, with a N-terminal or C-terminal V5 tag, respectively, Fig. 5C,D). We performed chromatin immunoprecipitation followed by sequencing (ChIP-seq) using the anti-V5 antibody and retrieved a set of reproducible peaks (*N* = 53,245, Fig. 5E; Dataset EV4). The strongest peaks (top 3%, 1600 peaks) were characterized by an enrichment for the TWIST1 motif (Fig. 5F) and were strongly significantly overlapping (91.2%, *p* < 10^−5^) with a list of TWIST1-bound regions obtained in a previous study (Fig. 5F) (Chang et al, 2015). These top peaks were mostly located on active enhancers (59%, Fig. 5G), including regions proximal to the *NREP* gene locus (Fig. 5H,I), suggesting that TWIST1 can directly control the expression of *NREP*. Notably, top peaks were located on the promoter of many validated miR-29b targets, such as collagen genes (e.g., *COL1A1*, *COL4A1-2*, *COL5A2*, Fig. 5H), which were also found to be responsive to *NREP* perturbation. Together, these data suggest a double feedback regulatory loop, involving TWIST1 and *NREP*, that promotes the expression of collagen genes and mesenchymal traits through convergent mechanisms (both transcriptional and post-transcriptional via TDMD; Fig. 5J). This regulatory mechanism acts in the opposite direction to GATA3 and miR-29b-3p and supports a role of the TDMD mechanism in fine-tuning phenotypic and transcriptional plasticity of breast cells.

### The *NREP*:miR-29b-3p axis is involved in cancer cell transcriptional plasticity

**Figure EV4:**
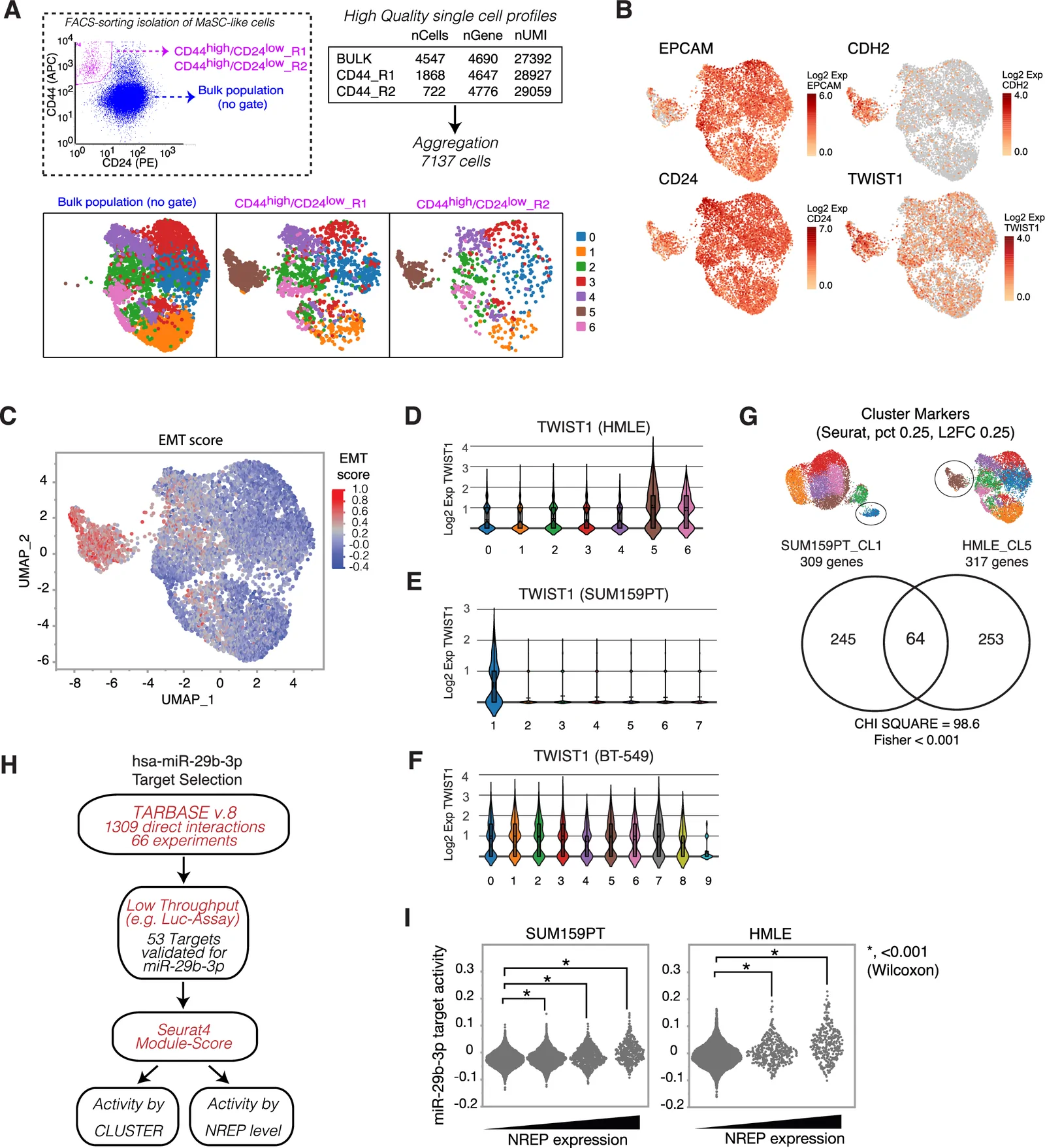
*NREP* controls miR-29b-3p levels and activity in CD44^high^/CD24^low^ HMLE cells. (**A**) Single-cell analysis of stem-like cells (maSC-like) in HMLE. Top: schematic workflow, with representative FACS profile of CD44^high^/CD24^low^ cells and summary of cells/features analyzed. Bottom: UMAP visualization of the aggregate dataset (7137 cells), grouped by samples and colored by clusters. Clusters were identified using standard methods (see Methods) based on transcriptional similarity within each sample; cluster numbers are arbitrary and not comparable across different cell lines. (**B**) UMAP plot of HMLE cells, colored by the expression of typical epithelial (EPCAM, CD24) or mesenchymal (CDH2 and TWIST1) markers. (**C**) UMAP plot colored by the EMT module score (see Methods). (**D**,** F**) Violin plots of TWIST1 expression across HMLE clusters (**D**), SUM159PT clusters (**E**), and BT-549 clusters (**F**). (**G**) Overlap between the mesenchymal clusters in SUM159PT and HMLE cell lines based on cluster markers. (**H**) Strategy for the definition of miR-29b-3p targets activity in single-cell gene expression profiles. (**I**) Activity of miR-29b-3p targets stratified according to different bins of NREP expression (bins are defined as mean, +1 SD, +2 SD, +3 SD; the first bin contains all cells with zero values). Statistical analysis by the Wilcoxon test, comparing each cluster versus all the others. *p < 0.001.

**Figure 6:**
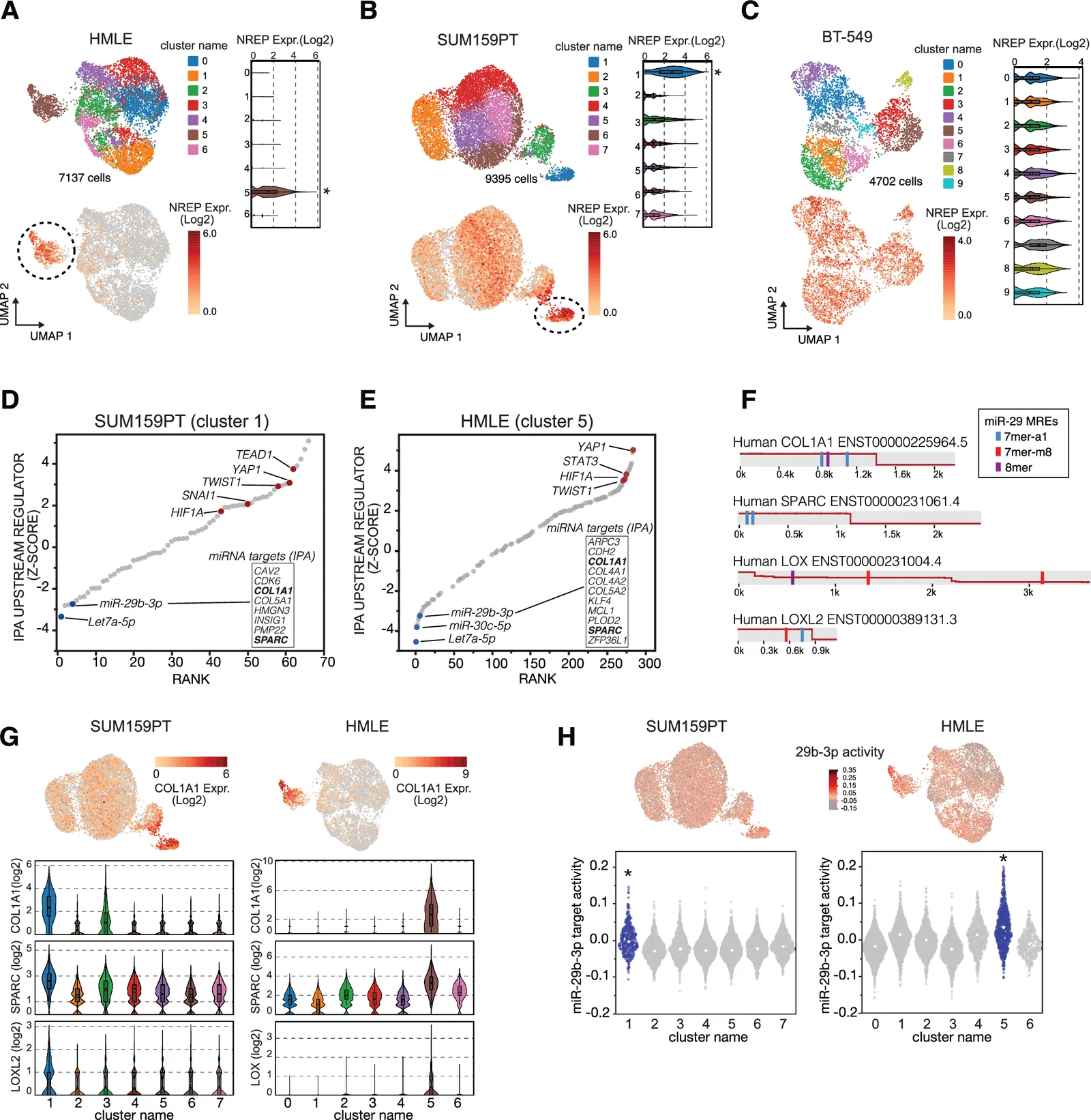
The NREP:miR-29b-3p axis is involved in fine-tuning epithelial plasticity. (**A**–**C**) UMAP representation of scRNA-seq data from HMLE (panel **A**, *N* = 2), SUM159PT (panel **B**, *N* = 2), and BT-549 (panel **C**, *N* = 1). For each panel, top: cluster assignment, bottom: the expression of *NREP* (log normalized). On the right, a violin plot reports the distribution of *NREP* expression by cluster. Statistical analysis by the Wilcoxon test, comparing each cluster versus all the others. *p < 0.001. A circle marks the cluster with the highest NREP expression. (**D**,** E**) The plot shows the results from the Upstream Regulator analysis (Ingenuity Pathway analysis, IPA) performed using the 309 markers from Cluster 1 (SUM159PT, panel **D**) or the 317 markers from Cluster 5 (HMLE, panel **E**). In the plot are reported the upstream transcription/regulation factors ranked according to the Z-scores (y-axis, from low to high). All entries have a *p* < 0.001. Some of the most significant positive and negative regulators are highlighted in red and in blue, respectively. The targets of miR-29b-3p predicted by IPA are shown in the insert box. (**F**) 3’ UTR regions of *COL1A1*, *SPARC*, *LOX*, and *LOXL2* genes. Shown are the TargetScan predicted binding regions for the miR-29 family. (**G**) Expression of *COL1A1*, *SPARC*, *LOX*, and *LOXL2* genes in scRNA-seq of SUM159PT and HMLE. top: UMAP representation of *COL1A1* expression (log normalized); bottom: violin plots reporting the distribution of gene expression by cluster. (**H**) miR-29b-3p activity was calculated in scRNA-seq profile (as by Seurat3 *ModuleScore* function; see Methods). Shown are the UMAP (top) and the violin plots (bottom) reporting the distribution of miR-29b-3p activity for each cluster. Statistical analysis performed by the Wilcoxon test, comparing each cluster versus all the others. *p < 0.001. Source data are available online for this figure.

We explored the relationship between *NREP*, miR-29b-3p and EMT plasticity by single-cell RNA sequencing in HMLE, SUM159PT and BT-549. For HMLE cells, we performed single-cell analysis on either the bulk population or FACS-sorted CD44^high^CD24^low^ cells, which represent only a minor subpopulation (<1%, Fig. EV4A). After aggregation of the single-cell datasets, a CD44^high^CD24^low^ specific cluster of cells (CL5) emerged, showing distinctive features of EMT and stemness (Fig. EV4B–D). Strikingly, *NREP* expression was confined only to the CD44^high^CD24^low^ cluster, where *TWIST1* was strongly enriched (Figs. 6A and EV4B,D). Heterogeneity in *NREP* and *TWIST1* expression was found in SUM159PT, but not in BT-549. In BT-549, the two genes were highly expressed in all clusters, suggesting that this cell line represents a more advanced stage of EMT (Figs. 6B,C and EV4E,F). Conversely, in SUM159PT, *NREP* and *TWIST1* expression were specifically associated with a distinctive cluster of cells (CL1, Figs. 6B and EV4E). A comparison of the two *NREP* specific subpopulations, namely HMLE-CL5 and SUM159-CL1, highlighted a significant similarity in gene expression markers (64 genes, *p* < 0.001 Fisher’s test, Fig. EV4G; Dataset EV5), and a similar set of putative “upstream regulator” (Ingenuity Pathway Analysis—IPA) with activation of EMT promoting factors (e.g., TWIST1, YAP1, and HIF1A, Fig. 6D,E; Dataset EV5). These subpopulations were characterized by low activity of tumor-suppressor miRNAs, most notably miR-29b-3p (Fig. 6D,E), with *COL1A1*, *SPARC, LOX*, and *LOXL2* among the most regulated targets (Fig. 6F,G). To corroborate these findings, we calculated miR-29b activity at single-cell resolution using the list of Tarbase-validated miR-29b targets (Fig. EV4H). A positive correlation between miR-29b targets and *NREP* levels was found in single-cell data (Fig. EV4I), and the two *NREP*-specific clusters, HMLE-CL5 and SUM159-CL1, showed the highest expression of miR-29b targets (Fig. 6H). Notably, a recent publication from our lab (Nadalin et al, 2024) has demonstrated the biological significance of this subpopulation (CL1), which is characterized by an enrichment of cells with tumor-initiating capacity (TIC-cells) and by the increased expression of specific collagen genes (Appendix Fig. S3). The transcriptional program of CL1 was found to be remarkably similar to a hybrid EMT metaprogram (Nadalin et al, 2024), which has been repeatedly associated to metastatic dissemination (Kroger et al, 2019; Simeonov et al, 2021). Taken together, these data strongly support a role for NREP in restricting miR-29b-3p activity at the level of specific cancer subpopulations, reinforcing the hypothesis of an *NREP*-miR-29b-3p axis involved in transcriptional plasticity of cancer cells and EMT transition.

### *SERPINE1*-mediated TDMD confers selective advantages to breast cancer cells

**Figure 7:**
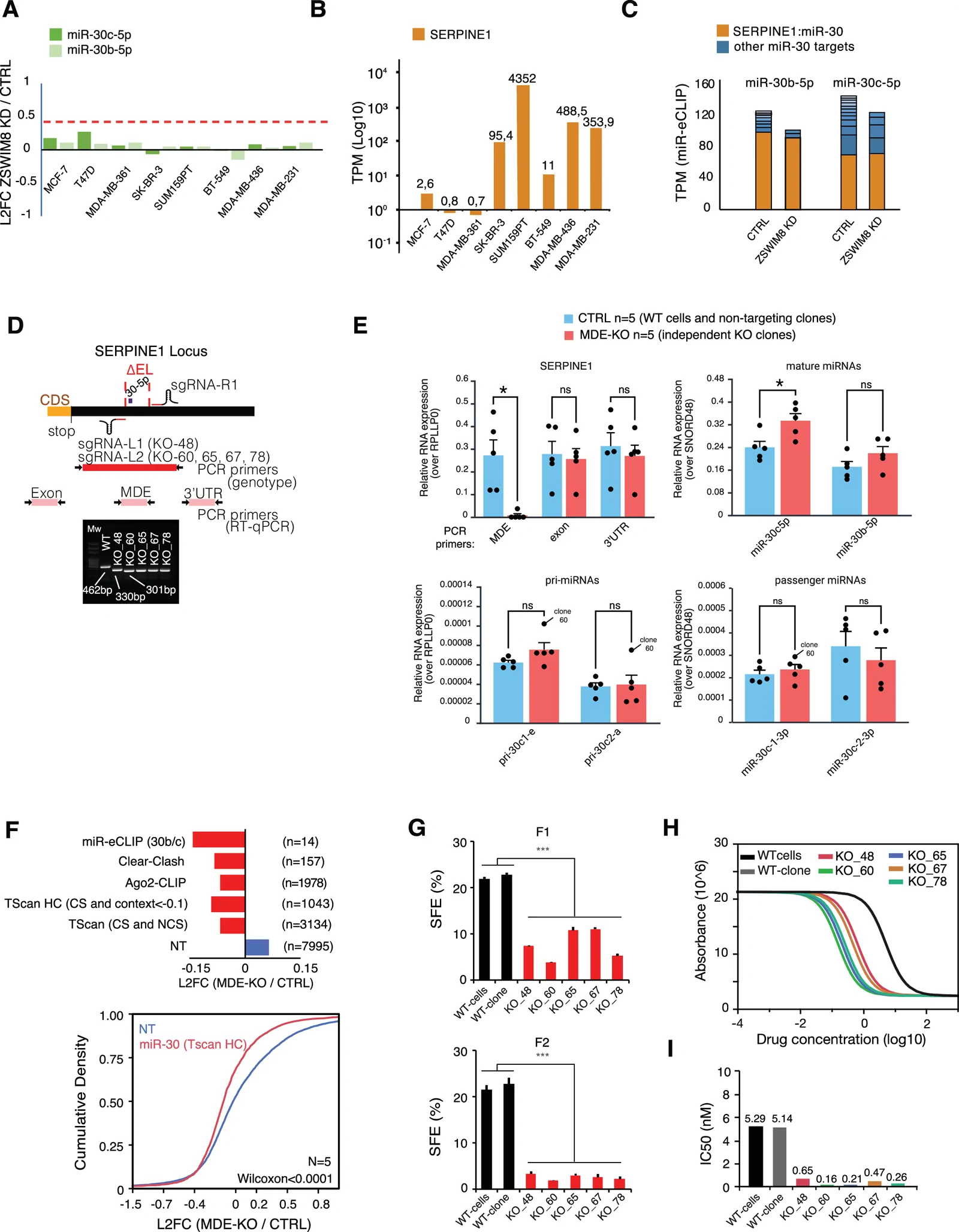
The disruption of SERPINE1:miR-30c interaction affects 3D growth and sensitivity to paclitaxel in breast cancer cells. (**A**) Differential expression of miR-30c-5p and miR-30b-5p in the BC cell lines, measured upon ZSWIM8 KD (data are from sRNA-seq experiments, Dataset EV1, *N* = 3). (**B**) Expression of *SERPINE1* in the same BC cell lines (TPM, by RNA-seq). (**C**) Stacked bar chart showing the normalized counts for each miR-30b-5p and miR-30c-5p target by miR-eCLIP in SUM159PT cells (as in Fig. 3F, *N* = 2). (**D**) Scheme showing details of the CRISPR-Cas9 strategy used for the removal of miR-30 degradation element (MDE) from the 3’ UTR of *SERPINE1*. Shown are the different sgRNAs used and the primer pairs used for genotyping and for RT-qPCR. (**E**) Expression levels by RT-qPCR of *SERPINE1* transcript, miR-30c-5p and miR-30b-5p, passenger strands and pri-miRNAs of miR-30c-5p across five independent MDE-KO clones. As controls, we employed parental SUM159PT cells and four different clones that were subjected to CRISPR-Cas9 but retained the wild-type MDE. For *SERPINE1*, different primer pairs were used, designed on the MRE, exon and the 3’ UTR. Data were normalized over *RPLP0* (for *SERPINE1* and pri-miRNAs) and *SNORD48* (for miRNAs). *P* values were calculated through a two-way ANOVA-test *p ≤ 0.05; *N* = 5, average ± SEM. (**F**) RNA expression analysis of controls and SERPINE1-MDE-KO clones by RNA-seq. Top: bar chart reporting the L2FC (MDE-KO vs CTRL, *N* = 5, as in (**E**), using different subsets of miR-30 targets (as in Fig. 3K). Bottom: cumulative distribution function (CDF) of the L2FC of genes (MDE-KO vs CTRL) comparing miR-30 predicted targets (CS with context score <−0.1) vs non-targets (NT, *N* = 12,733). Significance by the Wilcoxon test, *p* ≤ 0.05. (**G**) Sphere forming efficiency (SFE) assay performed on CTRLs (parental SUM159PT cells and one representative unedited clone) and five independent MDE-KO clones for *SERPINE1*. The SFE was measured both at the first (F1) and the second generation (F2) of spheres (*N* = 2; average in percentage and SEM). *P* values by Student’s *T*-test, ***p ≤ 0.001. (**H**,** I**) Sensitivity to paclitaxel was measured on CTRL and MDE-KO clones (as defined in (**G**); *N* = 5) for *SERPINE1*. Cell viability measured by ADP-Glo Max Assay in cells treated for 3 days with paclitaxel at different concentrations was used to calculate IC50 values. Shown are the logistic curves used to calculate drug sensitivity (panel **H**) and the bar chart reporting the IC50 measured (panel **I**). The results shown are from one of two independent experiments. Source data are available online for this figure.

**Figure EV5:**
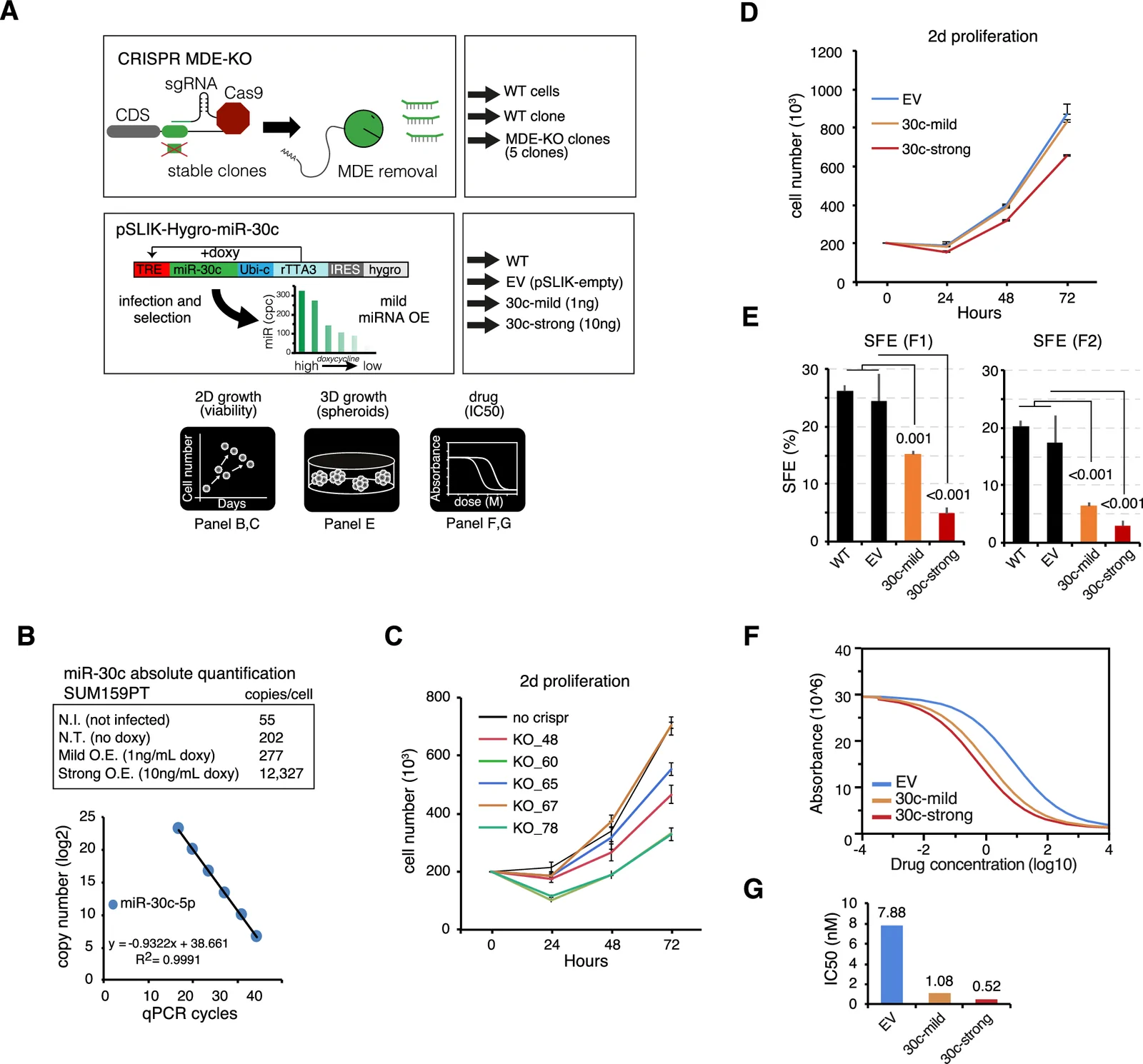
Phenotypes related to SERPINE1:miR-30c-5p interaction. (**A**) Schematic representation of the different methodological strategies used to investigate the phenotypes that depend on the SERPINE1:miR-30c-5p interaction. (**B**) Upper panel: a table summarizes miR-30c-5p levels by absolute quantification in cells infected with an inducible construct for miR-30c-5p overexpression. NI not infected cells, NT infected cells not treated with doxycycline. Lower panel: titration curve used for the absolute quantification of miR-30c-5p (see Methods). (**C**) Growth curve of SUM159PT clones bearing the MDE deletion in SERPINE1 3’UTR. *N* = 3 (average cell number and SEM). (**D**) Growth curve of SUM159PT cells upon mild or strong miR-30c-5p overexpression. *N* = 3 (average cell number and SEM). (**E**) Bar charts reporting the results of the sphere formation assay performed in SUM159PT. Shown is the sphere forming efficiency (average % and SEM from three replicate wells) measured at the first (F1) and at the second generation (F2). *N* = 2. *P* values by Student’s *T*-test. (**F**,** G**) Cell viability was measured by ADP-Glo Max Assay in SUM159PT cells treated for 3 days with Paclitaxel at different concentrations (panel **F**) and used to calculate IC50 values (panel **G**). Results shown are from one of two independent experiments.

*SERPINE1* is an endogenous TDMD trigger that can induce the degradation of miR-30b-5p and miR-30c-5p in mouse fibroblasts and in human cells. In silico analysis from our lab predicted *SERPINE1*:miR-30c-5p as a frequently occurring pan-cancer TDMD pair (Ghini et al, 2018; Simeone et al, 2022). Further, miR-30c-5p is generally downregulated in tumors, including breast (Bockhorn et al, 2013a; Bockhorn et al, 2013b). Surprisingly, when we knocked down ZSWIM8 in breast cancer cells, miR-30b-5p and miR-30c-5p were never found as potential TDMD substrates (Fig. 7A). Indeed, these two miRNAs have not been previously reported as substrates in any other study based on the loss of function of ZSWIM8 (Han et al, 2020; Jones et al, 2023; Shi et al, 2023; Shi et al, 2020). This result was particularly puzzling given that: (i) *SERPINE1* is very highly expressed in many basal/TNBC breast cancer cells, reaching very high expression in SUM159PT (TPM >1000, Fig. 7B); (ii) *SERPINE1*:miR-30b/c chimeric reads were abundant in the miR-eCLIP dataset in SUM159PT, accounting for the vast majority of the miR-30b/c target pool (Fig. 7C). Hence, we decided to investigate the *SERPINE1*:miR-30b/c interaction in SUM159PT cells by suppressing the TDMD pairing with CRISPR (Fig. EV5A), as previously performed in mouse fibroblasts (Ghini et al, 2018). We generated five independent clones bearing a small deletion in the *SERPINE1* 3’ UTR region that is involved in the interaction with miR-30 (Fig. 7D). As expected, *SERPINE1* mRNA expression was maintained, but the expression of the miRNA-degradation element (MDE) was abolished in the CRISPR-KO clones as compared to control clones (Fig. 7E). We observed a statistically significant increase of the levels of mature miR-30c-5p, and an increase, but not statistically significant, of miR-30b-5p (Fig. 7E). This observation is consistent with our previous experiments in mouse cells (Ghini et al, 2018) and reflects the stronger complementarity of miR-30c-5p with SERPINE1, as predicted by TDMDfinder. Therefore, we primarily focused on the *SERPINE1*:miR-30c-5p axis henceforth. Primary precursors of miR-30c-5p were not regulated, although some variability was observed between clones (e.g., KO-60, Fig. 7E) —in line with recent findings that CRISPR editing introduces some molecular and transcriptional variability (Prajapat et al, 2025). This supports the conclusion that *SERPINE1* functions as an endogenous TDMD trigger for miR-30c-5p in breast cells (Fig. 7E). RNA sequencing of parental and MDE-KO cells confirmed that the stabilization of miR-30c-5p impacted miR-30 activity, as endogenous targets predicted by TargetScan or obtained by Ago2-CLIP or CLEAR/CLASH experiments were significantly repressed (Fig. 7F; Dataset EV3). MiR-30c-5p is known to have a tumor-suppressor role in breast cancer, by increasing the sensitivity to chemotherapy drugs and inhibiting the growth of cancer stem cells (Bockhorn et al, 2013b; Chao et al, 2015; Yu et al, 2010). We monitored these cancer phenotypes in MDE-KO clones, comparing the results with those obtained by inducing a mild (~2-3-fold, in line with the mild induction produced by TDMD) or strong (>10-fold) miR-30c-5p expression (Fig. EV5A,B). MDE-KO clones and cells with mild miR-30c-5p overexpression did not display any consistent effect on cell proliferation (Fig. EV5C,D), although some differences were observed between clones (e.g., KO-60). Nonetheless, all MDE-KO clones showed a marked impairment in the ability to form mammospheres in non-adherent conditions (Fig. 7G), an assay frequently used to study the properties of cancer stem cells (Dontu et al, 2003). A significant decrease in sphere-forming efficiency was observed at different generations, similar to what was obtained by mild miR-30c-5p overexpression (Figs. 7G and EV5E). Moreover, we measured the sensitivity of cancer cells to Paclitaxel, an “anti-microtubule agent” used in the treatment of various cancers, including breast (Foa et al, 1994). Both MDE-KO clones and miR-30c-5p over-expressing cells displayed a 5–10-fold increased sensitivity to Paclitaxel treatment (Figs. 7H, I and EV5F,G). Together, these data show that *SERPINE1*-directed miR-30c-5p degradation is active in breast cancer cells and has an impact on cancer phenotypes.

### A ZSWIM8-independent TDMD mechanism controls miR-30c-5p

**Figure 8:**
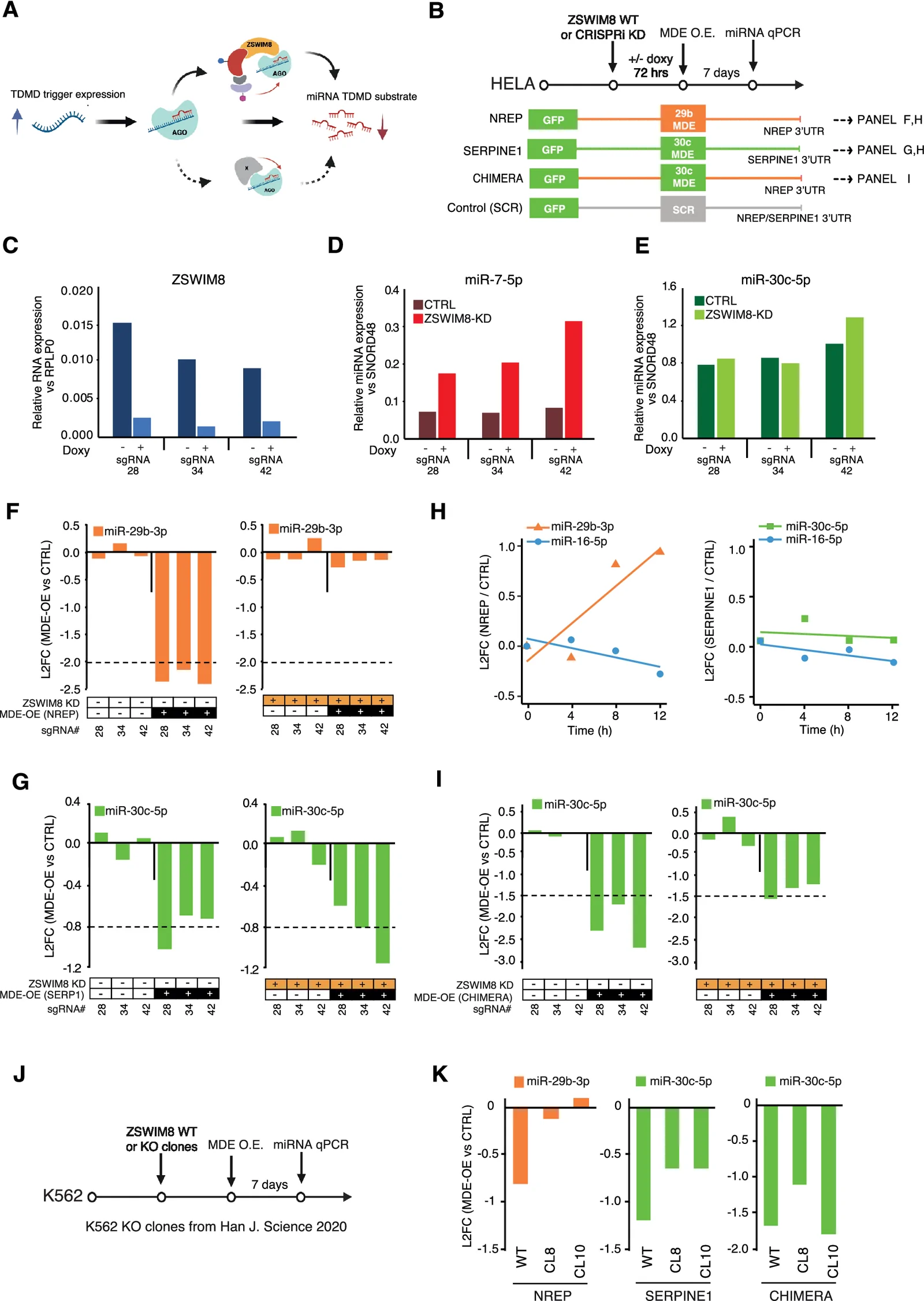
A ZSWIM8-independent TDMD mechanism controls miR-30c-5p. (**A**) Scheme summarizing a ZSWIM8-dependent and a ZSWIM8-independent TDMD pathway. (**B**) Scheme of the experimental strategy and constructs used to assess ZSWIM8-dependent and -independent TDMDs. CHIMERA contains an optimal MDE for miR-30c (a sequence complementary to the miRNA except at positions 9, 10, and 11) and the flanking region from the *NREP* transcript. (**C**–**E**) Expression levels of *ZSWIM8* (**C**), miR-7-5p (**D**), and miR-30c-5p (**E**) were measured by RT-qPCR in HeLa cells with (+) or without (−) perturbation of ZSWIM8 by CRISPRi for 7 days. Data were shown separately for each individual sgRNA. (**F**) TDMD effect on miR-29b-3p by *NREP* is ZSWIM8-dependent. Shown is the L2FC of miR-29b-3p levels (NREP-MDE vs Ctrl), measured in the absence of doxycycline (ZSWIM8 WT, on the left) or in the presence of doxycycline (ZSWIM8 KD, on the right). Data are shown separately for each individual sgRNA. (**G**) TDMD effect on miR-30c-5p by *SERPINE1* is ZSWIM8-independent. Shown the L2FC of miR-30c-5p levels (SERPINE1-MDE vs Ctrl), in the absence of doxycycline (ZSWIM8 WT, on the left) or in the presence of doxycycline (ZSWIM8 KD, on the right). Data were shown separately for each individual sgRNA. (**H**) The proteasome inhibitor MG132 was used on HeLa cells to rescue TDMD effects, as in (Shy et al, 2020). Left: miR-29b-3p and miR-16-5p (used as control) were measured by RT-qPCR upon overexpression of NREP-MDE in HeLa cells treated with MG132 (10 mM) at different time points. Right: miR-30c-5p and miR-16-5p measured by RT-qPCR upon overexpression of SERPINE WT. For both plots, miRNA expression levels were calculated as relative RNA expression over the *SNORD48* and normalized over a control MDE. The expression at time 0 with no treatment was used as a reference. Lines show the least-squares fit for each miRNA. (**I**) TDMD effect on miR-30c-5p was measured upon overexpression of the chimeric construct (see panel B). Shown the L2FC of miR-30c-5p levels (CHIMERA vs Ctrl), in the absence of doxycycline (ZSWIM8 WT, on the left) or in the presence of doxycycline (ZSWIM8 KD, on the right). Data were shown separately for each individual sgRNA. (**J**) Scheme of the experimental strategy to assess ZSWIM8-dependent and -independent TDMDs in ZSWIM8 knock-out K562 clones. (**K**) Shown are the L2FC of miR-29b-3p levels (NREP-MDE vs Ctrl) and miR-30c-5p (SERPINE1 or CHIMERA vs Ctrl), measured in wild-type K562 cells in two independent ZSWIM8 KO clones. Source data are available online for this figure.

We postulated that *SERPINE1* can promote degradation of miR-30c-5p by a TDMD mechanism, which does not require ZSWIM8. To prove the existence of a ZSWIM8-independent TDMD pathway, we performed a series of TDMD assays, based on the overexpression of a specific MDE coupled with the perturbation of ZSWIM8 (Fig. 8A,B). We generated ZSWIM8 CRISPRi in HeLa, a highly proficient cellular model in the TDMD assays (Simeone et al, 2022) and with low endogenous NREP and SERPINE1 levels. This choice provided an optimal testbed for the mechanistic dissection of TDMD, ensuring high perturbation efficiency and minimal endogenous interference from known TDMD triggers. Loss of ZSWIM8 was effective with three different sgRNAs and induced accumulation of miR-7-5p, but not miR-30c-5p (Fig. 8C–E), in line with previous data using the same cell line (Shi et al, 2020). As expected, NREP-MDE overexpression triggered miR-29b-3p degradation with fourfold repression, but the effect was almost completely abrogated upon ZSWIM8 KD, confirming that *NREP*:miR-29b axis is ZSWIM8-dependent (Fig. 8F). In contrast, SERPINE1-MDE overexpression triggered the degradation of miR-30c-5p with twofold repression, either in the presence or absence of ZSWIM8, showing that *SERPINE1* can trigger miR-30c-5p degradation via a ZSWIM8-independent post-transcriptional mechanism (Fig. 8G; Appendix Fig. S4A,B). It is known that the activity of the proteasome is essential for ZSWIM8-dependent TDMD (Han et al, 2020; Shi et al, 2020), thus we performed the TDMD assays while treating the cells with MG132, a largely used proteasome inhibitor. The expression of miR-29b-3p could be rescued by treatment with MG132, confirming that TDMD by NREP-MDE involved proteasome-mediated proteolysis (Fig. 8H). Conversely, miR-30c-5p levels were not affected by proteasome inhibition, demonstrating that miR-30c-5p degradation is independent of the ubiquitin-proteasome system (Fig. 8H). Finally, we generated a synthetic construct by placing an optimal TDMD pairing with miR-30c-5p in the 3’ UTR of *NREP* (Fig. 8B). This chimeric construct still induced miR-30c-5p degradation in a ZSWIM8-independent fashion (Fig. 8I; Appendix Fig. S4A,B), suggesting that the mechanism is likely associated with the miRNA substrate (i.e., miR-30c-5p) rather than the specific trigger (i.e., *SERPINE1*).

Since CRISPRi- mediated ZSWIM8 perturbation leaves residual transcript levels (~10% by RT-qPCR, see Fig. 8C), we considered the possibility that this remaining activity might be sufficient to elicit TDMD of miR-30c-5p. To rule out this scenario, we repeated the MDE overexpression assay in clonal ZSWIM8 knock-out K562 cells (Han et al, 2020) (Fig. 8J). In knock-out cells, TDMD of miR-29b-3p by NREP-MDE was completely abolished, as expected (Fig. 8K). In contrast, miR-30c-5p degradation by SERPINE1- and CHIMERA-MDE constructs remained largely active and occurred through a post-transcriptional mechanism (Fig. 8K; Appendix Fig. S4C,D). These data provide definitive evidence that miR-30c-5p degradation does not rely on ZSWIM8 and point to an alternative TDMD pathway.

## Discussion

### A catalog of TDMD-regulated microRNAs in breast cancer cell lines

The discovery of ZSWIM8 as a crucial factor for TDMD paved the way to the systematic identification of endogenous miRNA substrates. This strategy has recently been used to define the landscape of TDMD-regulated miRNAs in flies, nematodes, fishes and during mouse embryonic development (Jones et al, 2023; Shi et al, 2023). In this study, we exploited CRISPRi to perturb ZSWIM8 expression in a panel of breast cancer cell models, representing the major molecular subtypes (Luminal, Basal/TNBC, and HER2). In total, we found 33 miRNAs increased after ZSWIM8 KD. Most of the hits were identified only in single cell lines and with small fold changes. In addition, the identification of TDMD miRNA substrates is not straightforward, as changes in miRNA levels can be influenced by the presence of secondary effects. Therefore, we applied a stringent normalization process and included in a high confidence set only those miRNAs that showed minimal regulation at the transcriptional level (“TDMD net effect” ≥0.3 L2FC). Globally, this analysis identified 19 ‘High-Confidence miRNAs’ upon depletion of ZSWIM8. Among them, ten miRNAs were previously reported as substrates (Han et al, 2020; Jones et al, 2023; Shi et al, 2023; Shi et al, 2020), providing additional support to our results. Most substrates were regulated specifically in one cell line or in a specific tumor subtype (e.g., miR-29b-3p in Basal/TNBC and miR-146b-5p in Luminal). A similar pattern, with broad or specific control of miRNA substrates, was observed in embryonic development (Jones et al, 2023; Shi et al, 2023) and can be the result of a similar expression distribution of the target transcripts (“triggers”). The identification of cell line- and subtype-specific substrates suggests the potential of considering these RNA molecules as an innovative target for breast cancer treatment. Indeed, some of these miRNAs were previously associated to breast cancer progression, such as miR-29b-3p (Chou et al, 2013), miR-146b-5p (Tordonato et al, 2021), and miR-33b-5p (Lin et al, 2015). MiR-29b-3p displays a tumor suppressive activity by controlling a network of pro-metastatic factors involved in angiogenesis and remodeling of the tumor microenvironment, such as VEGFA, ANGPTL4, PDGF, LOX, and MMP9 (Chou et al, 2013). MiR-33a/b, in addition to its role in the regulation of lipid, cholesterol homeostasis and glucose metabolism (Rayner et al, 2010), inhibits breast cancer metastasis by targeting HMGA2, SALL4, and TWIST1 (Lin et al, 2015) and has been found repressed in most cancer types (Zhang et al, 2023). Finally, our group has linked miR-146b-5p depletion to reduced pharmacological resistance in breast cancer stem cells (Tordonato et al, 2021). These examples suggest that TDMD may contribute to cancer cell plasticity and tumor-associated transcriptional states by promoting the selective degradation of anti-tumor miRNAs in specific cellular contexts.

Importantly, even if regulating the levels of miRNAs only modestly, TDMD can affect miRNA activity on targets. We exploited miR-eCLIP to monitor transcriptome-wide the landscape of AGO-bound miRNA targets in control and ZSWIM8 perturbed cells. Integrating multiomics datasets (sRNA-seq and miR-eCLIP) and bioinformatics predictions (TDMDfinder) (Simeone et al, 2022), we could (i) verify that miRNAs target occupancy is affected by TDMD; (ii) recognize specific triggers, in particular we identified novel miR-33a/b-5p regulators (*ABCA1* and *HADHB1*); (iii) define the targetome of individual miRNAs, which was typically limited to a few transcripts (~10–20 targets per miRNA). On this latter point, miR-29b-3p captured our attention by showing an increased target occupancy towards a set of pro-metastatic genes in ZSWIM8 perturbed cells (Chou et al, 2013). Overall, we provide a complete framework of analysis to identify miRNAs under TDMD that are functionally relevant. We used miR-eCLIP to directly detect miRNA:target chimeric reads bound to AGO2, providing strong evidence of physical interaction in cells. A limitation of this study is that, due to the significant economic cost, we limited the analysis of miRNA activity by miR-eCLIP only to a specific cell line. Therefore, the functional relevance of the high-confidence miRNAs that were not addressed in SUM159PT will require additional studies. A second limitation of the study is that we have not generated MDE-KO clones for ABCA1 and HADHB to demonstrate that they act as TDMD triggers at the endogenous level. This is complicated by feedback regulatory loops between miR-33a/b and their host/target genes, which preclude straightforward interpretation of MDE deletions. MiR-33a and miR-33b are co-transcribed with sterol regulatory element–binding proteins (SREBP-1 and SREBP-2), which are master transcriptional regulators of lipid metabolism (Rayner et al, 2010). One of their direct transcriptional targets is ABCA1 (Rayner et al, 2010), a cholesterol-efflux pump that mediates retrograde sterol movement from the plasma membrane to the ER, thereby influencing SREBP1/2 maturation and activity. In parallel, miR-33a/b potently represses ABCA1 post-transcriptionally, with four binding sites in the 3’UTR, three of them being proximal to the predicted MDE (Rayner et al, 2010). This establishes a tight transcriptional and post-transcriptional feedback circuit, and MDE deletions in ABCA1 would likely trigger complex secondary effects, making interpretation of TDMD-specific outcomes particularly challenging. Therefore, while overexpression-based assays demonstrate that both ABCA1 and HADHB can induce degradation of miR-33a/b in a sequence- and pairing-dependent manner, further dissection of their endogenous roles will require more elaborate experimental designs.

### TDMD as a fine-tuning mechanism integrating cell plasticity transcriptional circuitries

By integrating expression data, *TDMDfinder* predictions, and miR-eCLIP chimeric reads, we identified NREP as the only candidate fulfilling all criteria for functional validation. We could confirm that *NREP* is the main (possibly only) trigger for miR-29b-3p. Notably, this regulatory axis is specifically active only in a subset of basal cell lines with mesenchymal features. We observed a remarkable heterogeneity in *NREP* expression, reflecting the specific induction of this gene along with different stages of the epithelial to mesenchymal transition. By monitoring miRNA activity at single-cell resolution and using breast plastic cell models (i.e., HMLE), we provided evidence that *NREP* might act synergistically with TWIST1 to foster EMT, restricting the expression and activity of miR-29b-3p. Interestingly, *NREP* was previously shown to spatially restrict miR-29b-3p expression in the cerebellum (Bitetti et al, 2018). We propose that this role is not limited to that specific tissue and that *NREP* might define miR-29b-3p temporally and spatially during the EMT process, which has a critical role in most types of cancer. In agreement with this hypothesis, we previously characterized the *NREP*:miR-29b-3p pair as ‘pan-cancer TDMD’ (Simeone et al, 2022). Here, we identify a set of miR-29b-3p targets that is responsive to NREP perturbation in cell lines, and that is positively associated with NREP expression across BRCA TCGA samples and other tumor types. Furthermore, it was recently found that NREP modulates tumor growth and metastasis in breast cancer models (Ruan et al, 2024). Whether this phenotype is specifically mediated by TDMD remains to be established, but it is tempting to speculate that TDMD plays a part. More generally, the *NREP* case showed that TDMD might be confined to specific cell subpopulations, suggesting that the effect size of this process is likely underestimated in bulk analyses. While additional studies are needed to explore the biological and therapeutic implication of this regulatory loop— particularly in the context of cancer stem cells—our findings demonstrate that TDMD-mediated repression is active in breast cells and contributes to the modulation of highly relevant cancer subpopulations. Although we did not perform direct phenotypic assays to assess tumor progression in this study, the association between NREP, miR-29b-3p repression and EMT-related subpopulations is consistent with prior in vivo and mechanistic studies and provides a strong rationale for further functional validation.

### ZSWIM8-independent TDMD mechanisms

In our panel of breast cancer cell lines, we failed to observe stabilization for miR-30b-5p and miR-30c-5p upon ZSWIM8 depletion, but we showed that TDMD by *SERPINE1* was active by the genetic removal of the miR-30 binding element in a TNBC cell line. Importantly, we previously published that endogenous SERPINE1 levels control miR-30c-5p by TDMD also in mouse embryonic fibroblast (MEFs) (Ghini et al, 2018). Conversely, a recent study on a ZSWIM8 knock-out mouse model failed to observe miR-30c-5p induction across multiple tissues, including MEFs (Shi et al, 2023). Therefore, SERPINE1 acts as a trigger for miR-30c-5p in a ZSWIM8-independent manner in multiple contexts, and the mechanism appears conserved across species, suggesting that the underlying regulatory logic is evolutionarily selected. Following this finding, we exploited an MDE-OE assay to formally demonstrate that miR-30c-5p TDMD does not require ZSWIM8 nor the ubiquitin-proteasome system, pinpointing to the existence of alternative mechanisms in mammalian cells. Recent evidence further supports this view, showing that Cyrano-like and Serpine1-like TDMD sites can be artificially repurposed to induce degradation of other miRNAs, including miR-155, even in the absence of ZSWIM8 (Ortega et al, 2024). Together with our findings on endogenous SERPINE1-mediated decay, these data support the existence of alternative TDMD routes that do not rely on the canonical ZSWIM8–ubiquitin–proteasome axis. This is consistent with the observation that numerous miRNAs are insensitive to ZSWIM8 depletion (Han et al, 2020; Shi et al, 2023) despite the presence of predicted TDMD triggers for many of them (Simeone et al, 2022). Notably, miR-155 has been reported to exhibit relatively rapid turnover (Kingston and Bartel, 2019; Marzi et al, 2016), raising the possibility that additional miRNAs undergo ZSWIM8-independent decay, although the endogenous triggers responsible for such a mechanism remain to be identified. A comprehensive and unbiased dissection of the ZSWIM8-independent mechanism would require dedicated screening approaches analogous to those previously used to identify factors involved in miR-7–Cyrano-mediated TDMD (Han et al, 2020; Shi et al, 2020). However, implementing a similar strategy for miR-30c-5p is technically challenging. The mir-30 family comprises multiple members sharing an identical seed sequence, and miR-30c-5p is generated from distinct genomic loci, complicating the design of selective reporters and genetic perturbations that would allow unambiguous interpretation of genome-wide screens. Therefore, while our data clearly demonstrate the existence of a conserved, ZSWIM8-independent TDMD event, a full mechanistic dissection will require dedicated future studies. Possible involvement of 3′-end modifications warrants consideration. Tailing and trimming are features previously associated with miRNA turnover (Han and Mendell, 2023). In our previous work, *SERPINE1-*mediated miR-30c-5p degradation in mouse fibroblasts was accompanied by an increase in adenylated miRNA isoforms (Ghini et al, 2018). However, recent studies have shown that 3′ tailing is not required for ZSWIM8-dependent TDMD, and adenylation cannot be considered an obligate “death mark” (Han et al, 2020; Kleaveland et al, 2018; Shi et al, 2020). Rather, enrichment of adenylated species may reflect selective degradation of non-adenylated isoforms, resulting in a kinetic enrichment of surviving species. Thus, while 3′-end modifications are associated with miRNA turnover, their precise mechanistic contribution to ZSWIM8-independent TDMD remains to be elucidated. Together, these observations support the view that TDMD does not rely on a single molecular mechanism, but rather represents a broader regulatory framework involving distinct, possibly miRNA-specific, decay pathways. In conclusion, the identification of a conserved ZSWIM8-independent TDMD event represents a conceptual boundary for ZSWIM8-based substrate discovery and is critical for the correct interpretation of ZSWIM8-perturbation datasets and associated phenotypic analyses.

## Methods

### Cell cultures and cell sorting

All cells were routinely checked for mycoplasma contamination and always tested negative. Cells carrying the PB-TRE-dCas9-KRAB construct were cultured in the same media of parental cell lines, but with 10% tetracycline free (TET FREE) FBS and 100 μg/mL Hygromycin B. SUM159PT were cultured under an atmosphere of 10% CO_2_ at 37 °C, while all other lines were kept at 5% CO_2_. Cell media are reported in the table below. CD44^high^CD24^low^ and CD44^low^CD24^high^ HMLE cells were isolated through FAC-sorting. Cells were blocked with PBS-BSA 10% for 1 h at 4 °C and then stained with CD44-APC (clone C26; BD Pharmigen) and CD24-PE (clone ML5; BD Pharmigen) antibodies for 45 min at 4 °C. Cells were sorted on a BD InFlux V7 flow cytometer. Clonal *ZSWIM8 knockout (ZWIM8* –/–) K562 cell lines (#8 and #10) were a kind gift of Prof. J. T. Mendell (Han et al, 2020).

**Table:** 

Cell line	Subtype	Medium	Hygro selection
SUM159PT	BASAL	Ham’s F12, 5% fetal bovine serum (FBS), 2 mM glutamine, 5 ug/mL insulin, 1 ug/mL hygrocortisone, 10 mM HEPES, 100ug/ml of P/S	200 ug/ml
MDA-MB-231	BASAL	DMEM, 10%FBS + 2mM L-glutamine + 1%P/S	200 ug/ml
BT-549	BASAL	DMEM, 10%FBS, 2 mM L-glutamine, 1%P/S	100 ug/ml
MDA-MB-436	BASAL	DMEM:Ham’s F12 medium (1:1 mixture) supplemented with 2 mM L-glutamine and 10%FBS + 1%P/S	100 ug/ml
T47D	LUMINAL	DMEM, 10%FBS, 2 mM L-glutamine, 1%P/S	100 ug/ml
MCF-7	LUMINAL	DMEM, 10%FBS, 2 mM L-glutamine, 1%P/S	100 ug/ml
MDA-MB-361	HER2	DMEM, 10%FBS, 2 mM L-glutamine, 1%P/S	100 ug/ml
SK-BR-3	HER2	DMEM, 10%FBS, 2 mM L-glutamine, 1%P/S	100 ug/ml
HMLE	Normal	MEGM:Dmem-F12 (1:1 mixture), 0.5 ug/mL hydrocortisone, 10 ug/ml insulin, 10 ng/ml EGF, 1%P/S	200 ug/ml
HeLa		DMEM + 10%FBS + 2mM L-glutamine + 1%P/S	200 ug/ml
K562		RPMI + 10%FBS + 1%P/S	

### Cloning of PB-TRE-dCas9-KRAB

The PB-TRE-dCas9-KRAB plasmid was generated by subcloning the cDNA sequence of the KRAB repressor domain from the pHAGE TRE dCas9-KRAB (pHAGE TRE dCas9-KRAB was a gift from Rene Maehr & Scot Wolfe, Addgene plasmid # 50917). The dCas9-KRAB sequence was amplified by PCR and cloned in frame into the PB-TRE-dCas9-VPR backbone (PB-TRE-dCas9-VPR was a gift from George Church, Addgene plasmid # 63800) within the AscI/AgeI sites. The cloning was sequence-verified by Sanger Sequencing.

### Generation of dCas9-KRAB cell lines by PiggyBac Transposition

Cells were seeded at 60–70% confluency the day before transposition in six-well plates. Cells were transfected according to the Lipofectamine 3000 protocol with 500 ng of transposon DNA (PB-TRE-dCas9-KRAB) and 200 ng of SuperPiggy-Bac transposase helper plasmid (Systems Bioscience). After 72 h from transfection, cells were put in selection with 200 μg/mL Hygromycin B. HMLE cells, which could not be efficiently transfected, were nucleofected with Amaxa P3 Primary cell 4D-Nucleofector kit from Lonza, according to the manufacturer’s instructions. Briefly, 100k cells were treated with a mixture of 1 μg of PB-TRE-dCas9-KRAB transposon DNA + 400 ng of SuperPiggy-Bac transposase helper plasmid, diluted in Nucleofector solution P3 + Supplement 1. Nucleofection was performed using Lonza Amaxa 4D Nucleofector with X unit and program EL-133 in a 20 μl-strip cuvette. HMLE were then seeded in MEGM medium until full recovery and selected with 100 μg/ml hygromycin for 1 week after nucleofection.

### Cloning of sgRNAs for CRISPRi

sgRNAs were cloned either in the lentiGuide-Puro (a gift from Feng Zhang, Addgene plasmid # 52963) or in the Cellecta-pRSGScribe1-EFS-TagRFP-2A-Puro-U6-sgRNA_ONLY-bc-3LTR (pRSGScribe1 hereafter) plasmids. LentiGuide cloning occurs within Esp3I sites. Forward and Reverse phosphorylated oligos (Merck) were annealed and diluted 1:200 in H_2_O for the subsequent ligation reaction with the LentiGuide plasmid. For the pRSGScribe cloning of sgRNA was performed by Gibson assembly, using dsDNA fragments of 300 bp (eBlocks, IDT) designed to contain the sequence of the sgRNA and 50 bp of overlapping regions with the linearized plasmid at both ends (AgeI / NdeI). The reaction was performed with 0.05 pmol of plasmid and 0.1 mol of insert at 50 °C for 15 min with NEBuilder HiFi master mix (NEB). All the constructs were sequence-verified by Sanger sequencing. The sgRNA sequences are reported in Dataset EV6.

### Lentiviral vectors packaging and infection

Viruses were produced in HEK293T at 80% of confluency with the following transfection mix: 20 μg Transfer Vector, 13 μg of psPAX2 (gag&pol) (Addgene #12260), 7 μg of pMD2G (envelope) (Addgene #12259), 94 μL of RT CaCl_2_ and water up to 750 μL. Then, 750 μL of 2xHBS were added dropwise, and 500 μL/10 cm plate of transfection mix was added to the cells. The medium was changed after 6 h and viruses harvested after 48 h, filtered (0.22μm) and ultracentrifuged for 2 h at 50,000 × *g* (rotor SW32 Ti, Beckman Coulter), at 4 °C. The viral pellet was resuspended in 300 μL of PBS 1X and stored at −80 °C. Cell lines used in this work were seeded at 50% confluency the day before the infection. Infection was carried out overnight, followed by media replacement. Selection started 48 h after the end of infection. Cells were treated with 0.5 μg/mL of Doxycycline for seven doublings to induce the knockdown of the gene of interest.

### Generation of SERPINE1-MRE knock-out clones by CRISPR

SgRNAs flanking the miRNA responsive element (MRE) of miR-30 contained in the SERPINE1 transcript were designed using the CRISPick design tool (Doench et al, 2016). SgRNAs were cloned into the PX458 vector (a gift from Feng Zhang, Addgene Plasmid #48138), and SUM159PT were co-transfected with combinations of left and right sgRNAs (sgRNA-L1 + sgRNA-R1 or sgRNA-L2 + sgRNA-R1, Fig. 6D) together with Cas9-GFP. At 48 h after the transfection, the top 5% of GFP+ cells were sorted as single cells into 96-well plates. After 2 weeks, deletions in clonal cells were screened by PCR using primers designed to detect bi-allelic deletions. Positive clones were confirmed by DNA sequencing. The protocol efficiency was about 66% for mono-allelic and 17% for bi-allelic deletions for SUM159PT cells. The PCR primers and the sgRNAs used in clone screening are reported in Dataset EV6.

### MDE overexpression (OE) assays

MDE sequences and controls (scrambled sequences) were sub-cloned within the BoxB sites of the lentiviral RNA motif plasmid cloning backbone (a gift from Paul Khavari, Addgene plasmid # 107253). SUM159PT and MDA-MB-436 dCas9-KRAB cells were infected, selected with puromycin and analyzed by RT-qPCR or sRNA-seq after 7 days. For the experiments combining MDE OE and ZSWIM8 perturbation (shown in Fig. 8), HeLa dCas9-KRAB cells were treated with 0.5 μg/mL doxycycline to induce the expression of the dCas9-KRAB protein and harvested after seven cell doublings from the induction. ZSWIM8–/– K562 clones and wild-type (WT) K562 cells were infected with MDE-OE constructs via spinoculation as follows: 1 × 10^6^ cells were resuspended in 2 mL of complete medium containing 80 µL of lentivirus (100× concentrated) and 1 µg/mL polybrene. Cells were then centrifuged at 800×*g* for 2 h at 32 °C. Infected K562 cells were harvested 8 days post infection for downstream analyses.

The full list of constructs is reported in Dataset EV6.

### Inducible expression of miR-30c

Doxycycline inducible expression of miR-30c was achieved using the pSLIK-Hygro system (a gift from Iain Fraser, Addgene plasmid # 25737) containing the native stem loop structure of the miRNA, plus an additional 300 bps of upstream and downstream DNA. The cassette was obtained through gene synthesis (GeneArt, ThermoScientific). As a negative control, the pSLIK-Hygro empty vector has been used.

### Sphere formation efficiency (SFE) assay

To obtain primary spheres, 200 cells were plated in ultra-low adherent 24-well plates in the presence of methylcellulose (MethoCult M3236, Stem Cell Technologies) diluted 1:1 in MEBM supplemented with 5 μg/ml insulin, 0.5 μg/ml hydrocortisone, 2% B27 (Invitrogen), 20 ng/ml EGF and bFGF (BD Biosciences), and 4 μg/ml heparin (Sigma). For second-generation assays, spheres were enzymatically and mechanically dissociated, and 200 viable cells were plated. Spheres were counted after 7 days, considering structures with a diameter >70 μm and a circularity >0.7. Image analysis was performed with Fiji (ImageJ).

### Cell viability analysis (Paclitaxel response)

Cells (10,000/well) were seeded in 96-well plates and treated with paclitaxel (1000, 100, 10, and 1 nM). After 72 h, cell vitality was measured by ADP-Glo™ Max Assay (Promega, V7001) following the manufacturer’s instructions.

### miRNA, pri-miRNA, and mRNA RT-qPCR expression analyses

Mature miRNAs were detected by RT-qPCR using the miRCURY LNA system (Qiagen) as by manufacturers’ instructions. Reverse transcription was performed using 10 ng of total RNA; cDNA was diluted 1:60, and 3 µL of the diluted cDNA were used for the reaction mix. RT-qPCR for primary transcripts (pri-miRNAs) was performed using the SuperScript VILO cDNA Synthesis Kit (Life Technologies) and the Fast SYBR green master mix (Life Technologies). Reverse transcription was performed using 1 µg of total RNA, and 25 ng of diluted cDNA were used for the reaction mix. All reactions were performed in triplicates. We designed pri-miRNA primers by visual inspection of the RNA sequencing tracks (bigwig) obtained from the corresponding cell lines in the UCSC Genome Browser. For intergenic and/or intronic miRNAs, we preferentially designed primers in a range of 500 base pairs upstream and downstream of the miRNA of interest. For intronic miRNAs, we designed additional primer pairs in exonic regions of the host gene. The sequences of each primer pairs were designed using Primer3 Plus. Primer pairs were tested for the linear amplification of their target with serial dilutions of cDNA. The complete list of the RT-qPCR primers used in this study is provided in Dataset EV6. For the absolute quantification of miR-30c-5p, a titration curve was built using a synthetic miR-30c-5p RNA oligo (Sigma). The oligo was diluted 1:10 in a range from 10^11^ to 10^6^ copies and then reverse transcribed as any other mature miRNA. A range from 10^6^ to 10^2^ copies was used as input for the qPCR and miR-30c-5p copies/cell were estimated from the linear fitting, considering an RNA/cell content of ~12 pg.

### Small RNA sequencing and data analysis

Total RNA was isolated with the miRNeasy Mini Kit (Qiagen). Small RNA sequencing (sRNA-seq) libraries were prepared using 1 μg of total RNA with the TruSeq Small RNA Kit (Illumina), following the manufacturer’s instructions. Sequencing was performed on an Illumina Novaseq 6000 (50 bp pair-end read mode at a 5 million read depth per sample). Sequencing quality was checked in the FASTQC report; all experiments showed a Phred quality score >30. MiRNA counting was performed with the *Isomirage* tool (Muller et al, 2014): mapped reads were then normalized based on the library size (reads-per-million), using the sum of all canonical and templated miRNA reads. All subsequent analyses were performed by removing low-expressed miRNAs (<10 RPM in Controls or ZSWIM8 perturbed cells). For the identification of differentially expressed miRNAs, we used a threshold of L2FC >0.3 (ZSWIM8 perturbed vs. control cells) and a *P* value by Student’s *t*-test <0.05. Statistical significance was further evaluated by EdgeR and DESeq2 using the raw counts from Isomirage and a two-factor experimental design (ZSWIM8 perturbed vs. controls), using an FDR-adjusted *p* value <0.05. Data were reported in Dataset EV1.

### RNA sequencing and data analysis

Libraries were generated from 500 ng of total RNA, using the TruSeq RNA Library Prep Kit v2 (Illumina). Sequencing was performed on an Illumina Novaseq 6000 at 50 bp pair-end read mode and 30 million reads. Reads were trimmed, filtered and aligned using STAR v2.7.3. Read count extraction and TPM normalization were performed using Feature Counts. Differential gene expression analysis was performed with the Bioconductor Deseq2 package v1.34 (Love et al, 2014), using default parameters. For miRNA-target analysis, miR-29 and miR-30 targets were downloaded from TargetScan 8.0, including information on conservation and the type of seed (8-mers, 7-mer-m8, and 7-met-1A). Annotation of experimentally validated targets was performed with Tarbase (v.8); targets identified from intramolecular ligation approaches (namely CLASH and CLEAR-seq) were from (Helwak et al, 2013; Moore et al, 2015); targets identified from AGO2-CLIP experiments were from the ENCORI database (2023 update version) (Li et al, 2014); miR-eCLIP targets were from this work. Data are reported in Dataset EV3. Pair-wise correlation analysis between the expression of NREP or miR-29b-3p with the targets of miR-29b-3p was performed using *cbioportal* (Gao et al, 2013), ENCORI/Starbase (Li et al, 2014), and GEPIA3 (Kang et al, 2025), using as input the TCGA datasets indicated in each figure.

### Single-cell RNA sequencing and data analysis

Cell lines were analyzed by scRNA seq (10X Genomics) either in bulk (BT-549, SUM159PT) or in FACS-sorted HMLE cells. Single-cell suspensions (500–1000 cells/μL) were mixed with reverse transcription mix using the 10x Genomics Chromium Single-cell 3’ reagent kit protocol V2 (BT-549 and HMLE) or V3.1 (SUM159PT) and loaded onto 10x Genomics Single-Cell 3’ Chips (www.10xgenomics.com). Libraries were generated according to manufacturers’ instructions and sequenced on Illumina Novaseq 6000 Sequencing System (with a single or dual indexing format according to the manufacturer’s protocol V2 or V3.1). We aimed at a coverage of 50 K reads per cell in each sequencing run. Read alignment, UMI counting and cell-containing barcode (CB) calling were performed using the *count* function from cellranger (v2.1 for BT-549 and HMLE, v6.0 for SUM159PT) on the human reference genome GRCh38. The different samples composing the single-cell HMLE dataset were aggregated using cellranger *aggr*, normalizing the depth across the input libraries. No Seurat-based integration, anchor-based alignment, or batch-correction procedure was applied. Subsequent analyses (quality filtering, normalization, dimensionality reduction, clustering, and differential gene expression) were carried out with Seurat (v4.0.5) (Hao et al, 2021). SUM159PT library was processed as previously reported (Nadalin et al, 2024). HMLE and BT-549 libraries were processed similarly. Briefly, the pipeline included: (i) filtering cells with >2000 and <5000 features and <5 percent mitochondrial genes, (ii) LogNormalization of the data with a 10000 scale factor, (iii) variable feature identification with *vst* method and nfeatures = 2000, (iv) cell clustering on scaled-data using as parameters k = 10 and r = 0.5, (v) UMAP reduction with RunUMAP using default parameters on the same input used for clustering, (vi) cluster markers identification using the *FindAllMarkers* function from Seurat, with parameters pct = 0.25 and L2FC = 0.25. Cluster categories and UMAP coordinates were then imported into Loupe software (v6.5) for graphical representation of selected genes by color-coded maps or violin plots. Functional analyses of upstream regulators of cluster markers (SUM159-CL1 and HMLE-CL5) were generated through the use of IPA (QIAGEN Inc.). Pathway activity (miR-29b target genes and EMT) was computed with *ModuleScore* function from Seurat, using as gene lists the set of 53 miR-29b target from Tarbase v.8 (reported in Dataset EV5) or the following EMT-markers (“*ZEB1”*, “*TWIST1*”, “*SNAI1”*, “*SNAI2*”, “*CDH2*”, “*VIM*”, “*KRT5*’,‘*TP63*”, “*FN1*”, “*ITGA6*”, “*ITGB4*”, “*SERPINE1*”), respectively.

### TWIST1 chromatin immunoprecipitation followed by sequencing

A N-terminal or C-terminal V5-Tagged TWIST1 cassette was obtained through gene synthesis (GeneArt, Thermo Scientific) and cloned into a pLVX-TetOne-Puro construct (Takara). HMLE cells were infected with ultracentrifuge-concentrated (100x) lentiviruses, selected with 1 μg/mL puromycin and then induced with 1 μg/mL doxycycline. TWIST1 induction was confirmed by western blotting after 72 h of doxycycline treatment (Antibody clone 2C1a, Santa Cruz; dilution 1:500). The preparation, processing, and sequencing of ChIP-seq samples were performed as previously described (Sabò et al, 2014). ChIP was performed with an anti-V5 antibody (10μg; Abcam, ab9116 ChIP grade). ChIP-seq libraries were prepared with 2–10 ng of DNA using an in-house protocol (Blecher-Gonen et al, 2013) and sequenced on an Illumina HiSeq 2000. ChIP-seq NGS reads were aligned to the human reference genome hg19 through the BWA aligner, using default settings. Peaks were called using the MACS2 software (v2.0.10) with the option “– mfold = 7,30 -*p* 0.00001”, thus outputting only enriched regions with *P* value <10^−5^. Promoter peaks were defined as all peaks with at least one base pair overlapping with the interval between −2 kb to +1 kb from the nearest TSS. The presence of Twist1 E-boxes was performed using the Trawler web-tool (Ettwiller et al, 2007) with default parameters, using as input the top 3% peaks (by *q* value) as reported by MACS2. For the overlap with HMEC enhancers, we retrieved the peaks of K4Me1 and K27Ac from the ENCODE datasets GSM733705 and GSM733660, respectively. Active enhancers were defined from the overlap of K4Me1 and K27ac histone marks. Twist1 peaks (Chang et al, 2015) were uplifted from h18 to hg19 before comparison with our dataset. Data were reported in Dataset EV4.

### miR-eCLIP and data analysis

miR-eCLIP experiments were performed and analyzed by Eclipse Bioinnovations Inc. The standard eCLIP protocol (Van Nostrand et al, 2016) was modified to enable chimeric ligation of miRNA and mRNA (Manakov et al, 2022). While plated, ~20 × 10^6^ cells were UV crosslinked at 400 mJoules/cm^2^ with 254 nm radiation, pelleted, snap frozen on ice dry and stored until use at −80 °C. Cell pellets were then lysed with 1 mL of eCLIP lysis mix and sonicated (QSonica Q800R2) for 5 min, 30 s on/30 s off with an energy setting of 75% amplitude, followed by digestion with RNase-I (Ambion). A primary mouse monoclonal AGO2/EIF2C2 antibody (sc-53521, Santa Cruz Biotechnology) was incubated for 1 h with magnetic beads pre-coupled to the secondary antibody (M-280 Sheep Anti-Mouse IgG Dynabeads, Thermo Fisher 11202D) and added to the homogenized lysate for overnight immunoprecipitation at 4 °C. Following overnight IP, 2% of the sample was taken as the paired size-matched input, with the remainder magnetically separated and washed with eCLIP high stringency wash buffers. Chimeric ligation was then performed on-bead at room temperature for 1 h with T4 RNA ligase (NEB). IP samples were then dephosphorylated with alkaline phosphatase (FastAP, Thermo Fisher) and T4 PNK (NEB) and an RNA adapter was ligated to the 3′ ends. IP and input samples were cut from the membrane at the AGO2 protein band size to 75 kDa above. Western blot was visualized using anti-AGO2 primary antibody (50683-RP02, SinoBiological) at a 1:2000 dilution, with TrueBlot anti-rabbit secondary antibody (18-8816-31, Rockland) at 1:6000 dilution. RNA adapter ligation, IP-western, reverse transcription, DNA adapter ligation, and PCR amplification were performed as previously described. Sequencing was performed as SE122 on the NextSeq 2000 platform. After sequencing, samples were processed with Eclipsebio’s proprietary analysis pipeline (v1). UMIs were pruned from read sequences using umi_tools (v1.1.1). Next, 3’ adapters were trimmed from reads using cutadapt (v3.2). Reads were then mapped to a custom database of repetitive elements and rRNA sequences. All non-repeat mapped reads were mapped to the genome (UCSC version GRCh38/hg38) using STAR (v2.7.7a). PCR duplicates were removed using umi_tools (v1.1.1). AGO2-eCLIP peaks were identified within eCLIP samples using the peak caller CLIPper (v2.0.1). For each peak, IP versus input fold enrichments and *p* values were calculated. miRNAs from miRBase (v22.1) were “reverse mapped” to any reads that did not map to repetitive elements or the genome using bowtie (v1.2.3). The miRNA portion of each read was then trimmed, and the remainder of the read was mapped to the genome using STAR (v2.7.7a). PCR duplicates were resolved using umi_tools (v1.1.1), and miRNA target clusters were identified using CLIPper (v2.0.1). Each cluster was annotated with the names of miRNAs responsible for that target. Peaks were annotated using transcript information from GENCODE release 41 (GRCh38.p13) with the following priority hierarchy to define the final annotation of overlapping features: protein coding transcript (CDS, UTRs, intron), followed by non-coding transcripts (exon, intron). To assess reproducibility, the modified coefficient of variation (MCV) was calculated on the logarithm of the normalized intensity of each peak (i.e., chimeric read counts per miRNA). The method compares the observed MCV (oMCV) values against a theoretical distribution representing reproducible peaks. It then estimates a reproducibility index, calculated as the probability of an oMCV value coming from the distribution of reproducible peaks. Since the actual distribution of reproducible peaks is unknown, the Empirical Bayes approach was used to first estimate a vector of mean intensities and a matrix of variance-covariances, where the dimension of these vectors and matrices is equal to the number of replicates in the experiment. Then, these parameters were used to generate intensity values for each peak randomly. The MCV is calculated in each random sample (rMCV) and compared against the oMCV. The proportion of times the oMCV is smaller than rMCV is then used to estimate the reproducibility index (a value between zero and one). A higher value indicates a greater probability that the peak is reproducible. A reproducible miRNA target peak is defined as a peak with ≥3 chimeric reads on average and a reproducibility index ≥0.5. To confidently identify statistically significant differences between sample groups, DESeq2 was used. First, the raw counts were normalized by genomic peaks across all our libraries, using relative log expression (REL). Next, the information from all the peaks was used to estimate the variability (dispersion) between samples for each peak in a way that considers the logarithmic nature of miR-eCLIP data (Wald test, adjusted for multiple comparisons using the FDR procedure). For each differential analysis performed, Dataset EV2 reports the coordinates of each chimeric peak, which gene and miRNA is associated with the peak, the average normalized chimeric read counts in each sample group, the associated fold change and −log_10_-transformed significance.

Differential miRNA target occupancy (ZSWIM8 vs control cells) was estimated either from the average L2FC of the peaks related to the same miRNA, or from the ratio of the sum of the normalized counts. In this second approach, highly abundant targets display a higher weight. The two methods yielded largely concordant results (compare Figs. 3D with EV2G).

### Other statistical analysis

We did not use any criteria to determine the sample size, which was comparable to that reported in previous studies. No blinding or randomization method was applied. All experiments were replicated at least twice, including single-cell RNA-seq. Gene perturbations by CRISPRi were performed with at least three independent sgRNAs. Gene perturbations by CRISPR-KO (ZSWIM8) were performed with at least two independent clones. ChIP of TWIST1 was performed by tagging the protein both at C-term and N-term. No data were excluded from the analysis.

### Graphics

Some of the graphics and the synopsis were created with BioRender.com.

## Supplementary information

Appendix

Peer Review File

Data Set EV1

Data Set EV2

Data Set EV3

Data Set EV4

Data Set EV5

Data Set EV6

Source data Fig. 1

Source data Fig. 2

Source data Fig. 3

Source data Fig. 4

Source data Fig. 5

Source data Fig. 6

Source data Fig. 7

Source data Fig. 8

Expanded View Figures

## Data Availability

All sequencing data generated in this study have been deposited in GEO with the accession numbers GSE237728 (miRNA-seq, single-cell RNA-seq of BT-549 and HMLE, bulk RNA-seq), GSE264419 (ChIP for TWIST1) and GSE263552 (miR-eCLIP). Single-cell RNA-seq data of SUM159 cells were previously published and are available in the ArrayExpress database under accession codes E-MTAB-13064 (https://www.ebi.ac.uk/biostudies/arrayexpress/studies/E-MTAB-13064). Meta-analysis from previous studies was performed using: validated miRNA targets from Tarbase (v8); AGO-CLIP targets from ENCORI v2023 (https://rnasysu.com/encori/); ChIP TWIST1 peaks from https://genesdev.cshlp.org/content/suppl/2015/03/10/gad.242842.114.DC1/Supp_Table1_TwistPeakLocation_hg18.xls); ENCODE enhancers for HMLE cells from GSM733705 and GSM733660. predicted miRNA targets from TargetScan (v8). This study did not involve the use of any custom code. All analyses were performed using standard, publicly available software tools as described in the Methods section. The source data of this paper are collected in the following database record: biostudies:S-SCDT-10_1038-S44318-026-00889-8.
