# Supplementary material for: Canonical and non-canonical miRNA degradation shapes state transitions and stemness in breast cancer
Source: EMBO J. 2026 Aug 17;45(19):6813–46. doi: 10.1038/s44318-026-00889-8 (PMC13631368; doi:10.1038/s44318-026-00889-8)
Supplement: Supplementary file 1 [file 44318_2026_889_MOESM17_ESM.pdf]

Expanded View Figures

Figure EV1. Identification of high-confidence TDMD miRNA substrates.

(**A**, **B**) For each miRNA found as upregulated upon ZSWIM8 KD (see Fig. 2E) is reported the distribution of reads from the 5p/3p strands, the TDMD net effect (red bar) and the values (L2FC values upon ZSWIM8-KD) of miRNA substrate (red dots) and matched miRNA (pink) obtained by sRNA-Seq analysis, together with their pri-miRNAs (green), and host genes (blue dots) obtained by RT-qPCR, normalized with either RPLP0 or GADPH (two or more dots are shown, considering the two normalizations and different pair of primers used). A dashed bar indicates the threshold for the "TDMD net effect" (≥0.3 L2FC). $N = 3$. (**C**) Regulation of TDMD substrates identified in this study in other human cell lines (left K562; right HEK293; data from (Han et al, 2020)). The graphs show the fold change of miRNA levels assessed upon ZSWIM8 KD over the average expression of the controls. The red dots represent the breast cancer substrates that were detected (>10 TPM) in the corresponding cell line; miRNAs showing an L2FC >0.3 were considered as "induced" and are bolded.

 © The Author(s)

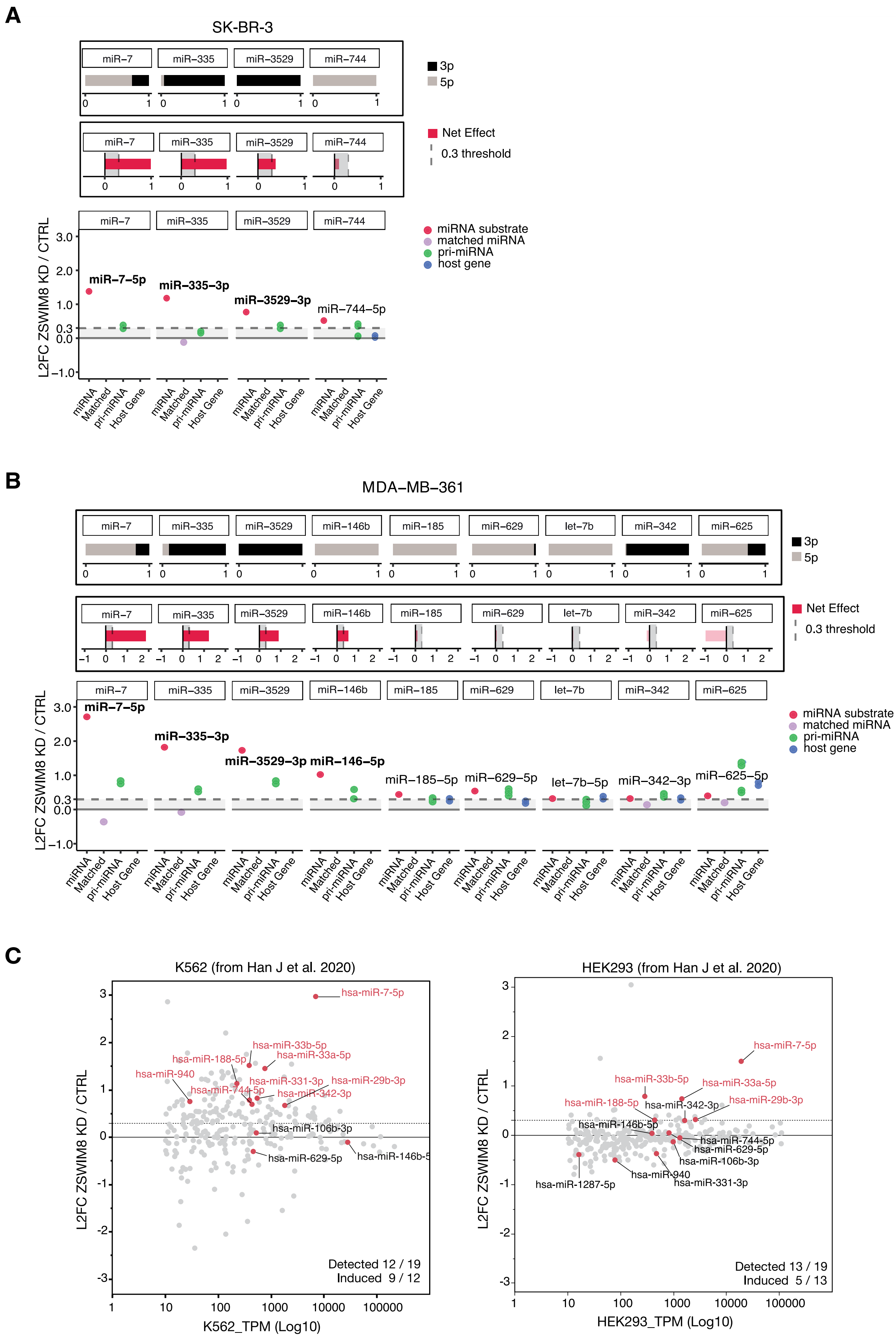
A
SK-BR-3
miR−7
miR−335
miR−3529
miR−744
3p
5p
Net Effect
0.3 threshold
miRNA substrate
matched miRNA
pri-miRNA
host gene
L2FC ZSWIM8 KD / CTRL
miR−7−5p
miR−335−3p
miR−3529−3p
miR−744−5p
miRNA
Matched
pri-miRNA
Host Gene
B
MDA−MB−361
miR−146b
miR−185
miR−629
let−7b
miR−342
miR−625
miR−146−5p
miR−185−5p
miR−629−5p
let−7b−5p
miR−342−3p
miR−625−5p
C
K562 (from Han J et al. 2020)
HEK293 (from Han J et al. 2020)
K562_TPM (Log10)
HEK293_TPM (Log10)
Detected 12 / 19
Induced 9 / 12
Detected 13 / 19
Induced 5 / 13
hsa-miR-7-5p
hsa-miR-33b-5p
hsa-miR-33a-5p
hsa-miR-188-5p
hsa-miR-940
hsa-miR-744-5p
hsa-miR-331-3p
hsa-miR-29b-3p
hsa-miR-342-3p
hsa-miR-106b-3p
hsa-miR-629-5p
hsa-miR-146b-5p
hsa-miR-1287-5p

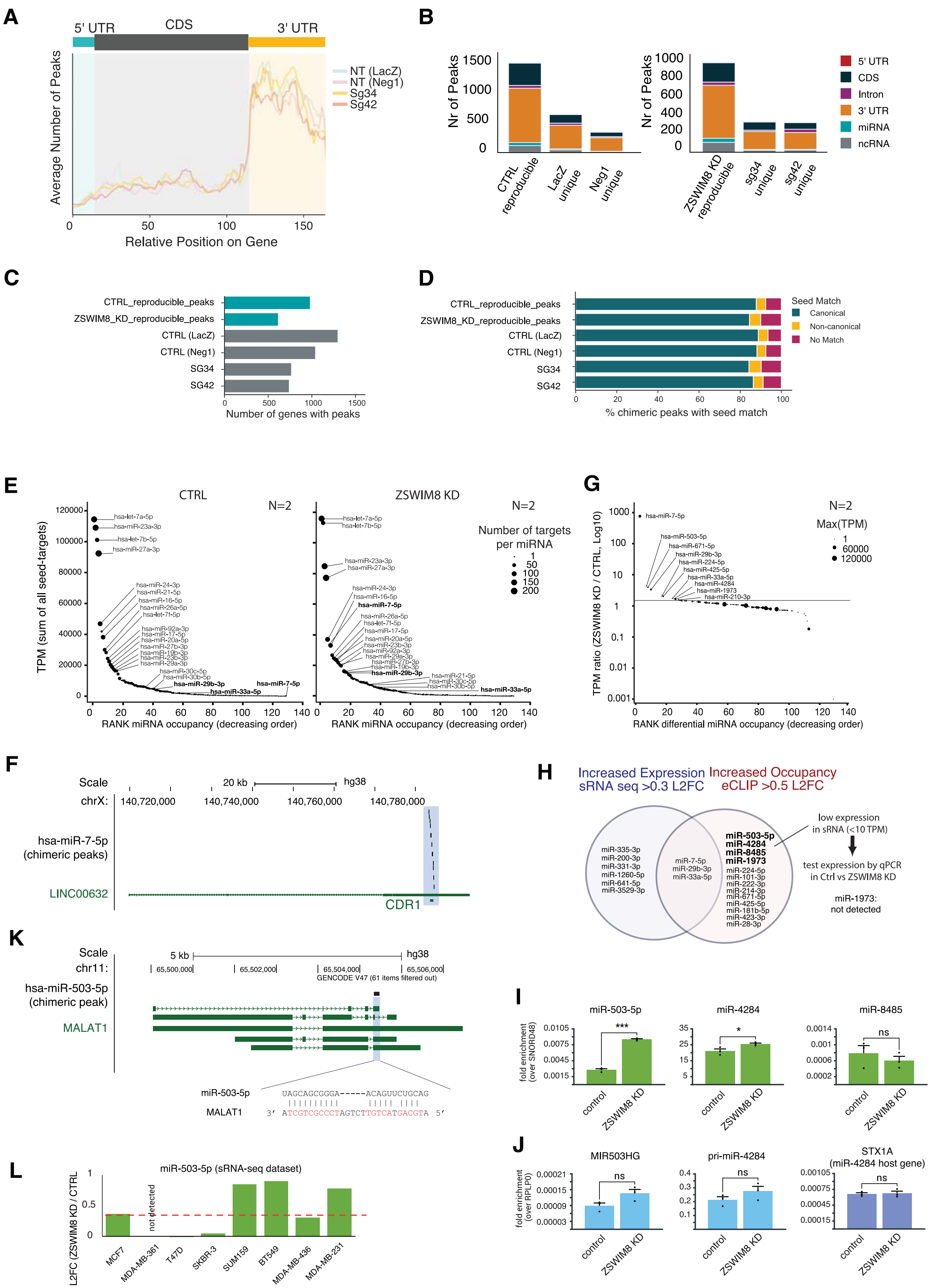
A
5' UTR
CDS
3' UTR
Average Number of Peaks
Relative Position on Gene
NT (LacZ)
NT (Neg1)
Sg34
Sg42
B
Nr of Peaks
CTRL reproducible
LacZ unique
Neg1 unique
ZSWIM8 KD reproducible
sg34 unique
sg42 unique
5' UTR
CDS
Intron
3' UTR
miRNA
ncRNA
C
CTRL_reproducible_peaks
ZSWIM8_KD_reproducible_peaks
CTRL (LacZ)
CTRL (Neg1)
SG34
SG42
Number of genes with peaks
D
Seed Match
Canonical
Non-canonical
No Match
% chimeric peaks with seed match
E
CTRL
ZSWIM8 KD
N=2
TPM (sum of all seed-targets)
RANK miRNA occupancy (decreasing order)
Number of targets per miRNA
G
TPM ratio (ZSWIM8 KD / CTRL, Log10)
RANK differential miRNA occupancy (decreasing order)
Max(TPM)
F
Scale
20 kb
hg38
chrX:
hsa-miR-7-5p (chimeric peaks)
LINC00632
CDR1
H
Increased Expression sRNA seq >0.3 L2FC
Increased Occupancy eCLIP >0.5 L2FC
low expression in sRNA (<10 TPM)
test expression by qPCR in Ctrl vs ZSWIM8 KD
miR-1973: not detected
K
Scale
5 kb
hg38
chr11:
hsa-miR-503-5p (chimeric peak)
MALAT1
GENCODE V47 (61 items filtered out)
I
miR-503-5p
miR-4284
miR-8485
fold enrichment (over SNORD48)
control
ZSWIM8 KD
J
MIR503HG
pri-miR-4284
STX1A (miR-4284 host gene)
fold enrichment (over RPLP0)
L
miR-503-5p (sRNA-seq dataset)
L2FC (ZSWIM8 KD / CTRL)
not detected
MCF7
MDA-MB-361
T47D
SKBR-3
SUM159
BT549
MDA-MB-436
MDA-MB-231

 © The Author(s)

Figure EV2. miR-eCLIP identifies endogenous miRNA:target interactions.

(**A**) Metagene plot showing the average number of AGO2 peaks along the 5′UTR, CDS, and 3′UTR for peaks called in each sample (control sgRNA118 and 119; ZSWIM8 KD sgRNA34 and 42). In order to create the metagene, peaks were downsampled to match the peak set with the smallest number to account for differences in the number of significant peaks and gene lengths were normalized. (**B**) The AGO2 peak upset plot shows the feature distribution for reproducible peaks, as well as unique peaks for each replicate, in control (left) or ZSWIM KD cells (right). (**C**) Number of genes with chimeric peaks in each sample or reproducible across replicates (see Methods for the identification of reproducible peaks). (**D**) Proportion of seed match among chimeric peaks. Canonical sites are 6mer, 7mer-1A, 7mer-m8, and 8mer; noncanonical sites are seed-less: 3′ compensatory or centered. (**E**) Bubble plot showing miRNA target occupancy in control (left) or ZSWIM8 KD (right) cells, as estimated from the cumulative count of all targets found in chimeric reads (expressed in transcript per million, TPM). MiRNAs are ranked from high to low occupancy. The dimension of the bubbles is proportional to the number of targets. (**F**) Chimeric peaks for hsa-miR-7-5p and LINC00632. All the peaks localize on the CDR1as region. (**G**) Bubble plot reporting the differential miRNA activity (ZSWIM8 KD vs Ctrl), calculated by a ratio of the values reported in panel E. The dimension of the bubble is proportional to the max miRNA target occupancy observed either in control or ZSWIM8 KD cells. (**H–J**) MiRNAs with increased target occupancy by eCLIP and low expression by sRNA-seq were re-analyzed by RT-qPCR in control and ZSWIM8 KD cells. MiR-1973 was not detected even by RT-qPCR. (**I**) Bar charts reporting the expression levels by RT-qPCR of mature miRNAs measured upon 0.5 µg/ml doxycycline (+) for seven cell doublings on cells infected with controls and ZSWIM8 targeting sgRNAs (average and SEM; $N = 3$). Data were normalized over *SNORD48*. Statistical analysis with the Student's *t*-test; *$p \leq 0.05$, ***$p \leq 0.001$. (**J**) Bar charts showing the expression levels of pri-miRNAs and host genes by RT-qPCR, as in (**I**). Data were normalized over RPLP0 (average and SEM; $N = 3$). Statistical analysis with the Student's *t*-test. (**K**) Chimeric peak for hsa-miR-503-5p and MALAT1. The peak contains a region of MALAT1 displaying an extended complementarity to miR-503-5p, as shown. (**L**) sRNA-seq data for miR-503-5p were re-analyzed in all BC samples (as in Fig. 1), including those with miR-503-5p expression <10 TPMs, and are reported as average L2FC between control and ZSWIM8 KD cells (see also Dataset EV1).

© The Author(s)

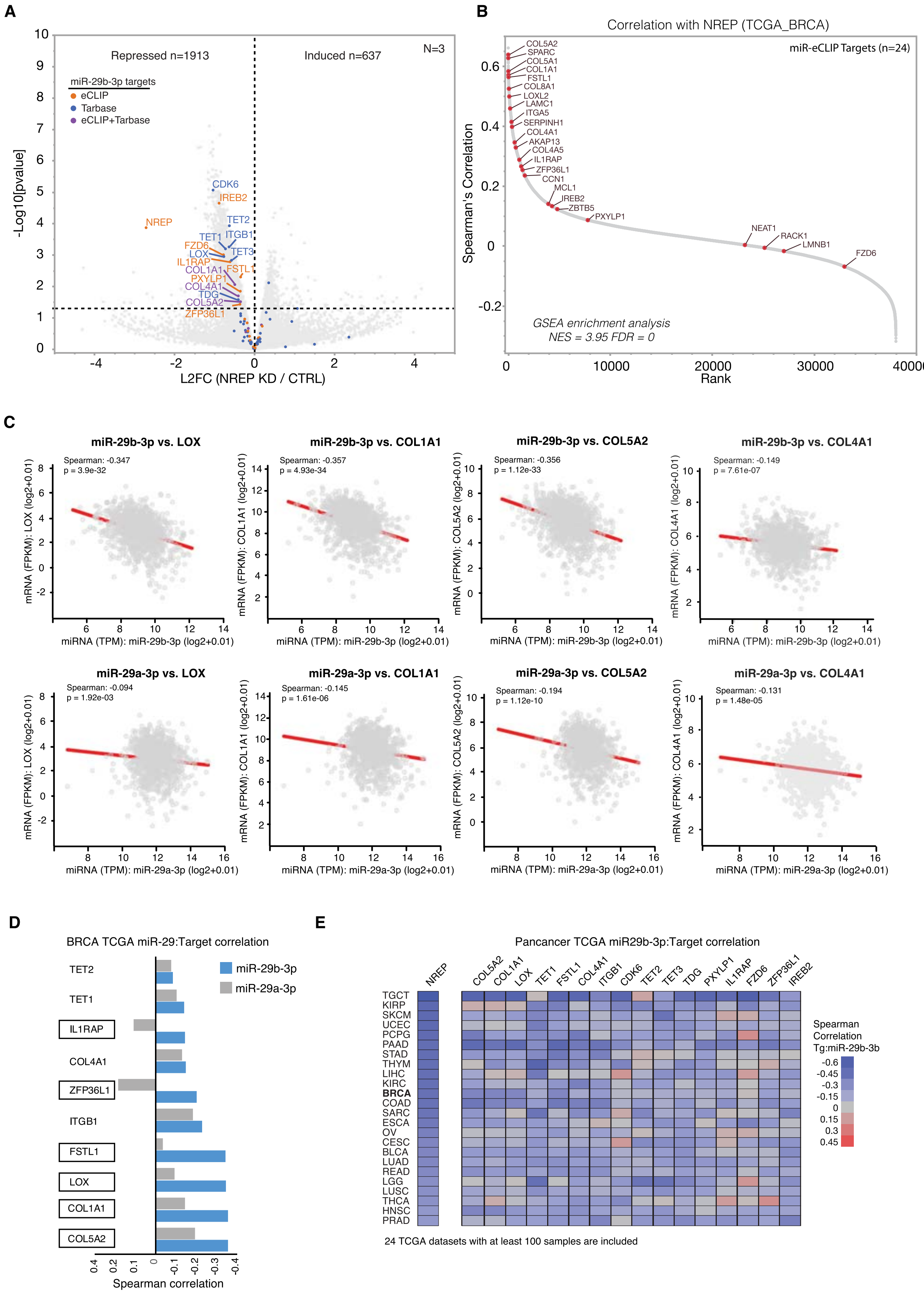
A
N=3
Repressed n=1913
Induced n=637
miR-29b-3p targets
eCLIP
Tarbase
eCLIP+Tarbase
CDK6
IREB2
NREP
TET2
TET1
ITGB1
FZD6
LOX
TET3
IL1RAP
COL1A1
FSTL1
PXYLP1
COL4A1
TDG
COL5A2
ZFP36L1
-Log10[pvalue]
L2FC (NREP KD / CTRL)
B
Correlation with NREP (TCGA_BRCA)
miR-eCLIP Targets (n=24)
COL5A2
SPARC
COL5A1
COL1A1
FSTL1
COL8A1
LOXL2
LAMC1
ITGA5
SERPINH1
COL4A1
AKAP13
COL4A5
IL1RAP
ZFP36L1
CCN1
MCL1
IREB2
ZBTB5
PXYLP1
NEAT1
RACK1
LMNB1
FZD6
Spearman's Correlation
GSEA enrichment analysis
NES = 3.95 FDR = 0
Rank
C
miR-29b-3p vs. LOX
Spearman: -0.347
p = 3.9e-32
miR-29b-3p vs. COL1A1
Spearman: -0.357
p = 4.93e-34
miR-29b-3p vs. COL5A2
Spearman: -0.356
p = 1.12e-33
miR-29b-3p vs. COL4A1
Spearman: -0.149
p = 7.61e-07
miR-29a-3p vs. LOX
Spearman: -0.094
p = 1.92e-03
miR-29a-3p vs. COL1A1
Spearman: -0.145
p = 1.61e-06
miR-29a-3p vs. COL5A2
Spearman: -0.194
p = 1.12e-10
miR-29a-3p vs. COL4A1
Spearman: -0.131
p = 1.48e-05
mRNA (FPKM): LOX (log2+0.01)
mRNA (FPKM): COL1A1 (log2+0.01)
mRNA (FPKM): COL5A2 (log2+0.01)
mRNA (FPKM): COL4A1 (log2+0.01)
miRNA (TPM): miR-29b-3p (log2+0.01)
miRNA (TPM): miR-29a-3p (log2+0.01)
D
BRCA TCGA miR-29:Target correlation
miR-29b-3p
miR-29a-3p
TET2
TET1
IL1RAP
COL4A1
ZFP36L1
ITGB1
FSTL1
LOX
COL1A1
COL5A2
Spearman correlation
E
Pancancer TCGA miR29b-3p:Target correlation
NREP
COL5A2 COL1A1 LOX TET1 FSTL1 COL4A1 ITGB1 CDK6 TET2 TET3 TDG PXYLP1 IL1RAP FZD6 ZFP36L1 IREB2
TGCT KIRP SKCM UCEC PCPG PAAD STAD THYM LIHC KIRC BRCA COAD SARC ESCA OV CESC BLCA LUAD READ LGG LUSC THCA HNSC PRAD
Spearman Correlation Tg:miR-29b-3b
-0.6 -0.45 -0.3 -0.15 0 0.15 0.3 0.45
24 TCGA datasets with at least 100 samples are included

 © The Author(s)

◀ **Figure EV3.** ***NREP*** **perturbation increases miR-29b-3p activity.**

(**A**) Volcano plot showing differentially regulated genes (p value <0.05; NREP KD vs CTRL, $N = 3$; Deseq2) upon NREP perturbation in SUM159PT cells. Highlighted are miR-29b-3p targets identified by miR-eCLIP or from Tarbase. (**B**) Spearman pair-wise correlation analysis between the expression level of NREP and all other genes ($n = 38,005$) was calculated in the TCGA BRCA dataset. Genes are ranked according to the correlation with NREP (high to low). MiR-29b-3p targets, defined from miR-eCLIP chimeric peaks, are highlighted in red. The targets were used as a gene set to run gene set enrichment analysis (GSEA). The observed normalized enrichment score (NES = 3.95) indicates a strong positive association. (**C**) Top: Spearman pair-wise correlation analysis between the expression level of miR-29b-3p (x-axis, data in TPM) and selected target genes (y-axis, data in FPKM) calculated using the TCGA BRCA dataset. Bottom: the same analysis was performed with miR-29a-3p. (**D**) Bar chart summarizing the Spearman coefficient between miR-29b-3p (blue) or miR-29a-3p (gray) and *NREP*-dependent miR-29b-3p targets (i.e., top 10 from Fig. 4G). A box indicates genes showing stronger correlation with miR-29b-3p as compared to miR-29a-3p. (**E**) Heatmap showing the Spearman correlation of miR-29a-3p with *NREP*-dependent miR-29b-3p targets across 24 TCGA datasets (Breast cancer, BRCA, highlighted in bold). *NREP* is shown as a reference.

© The Author(s)

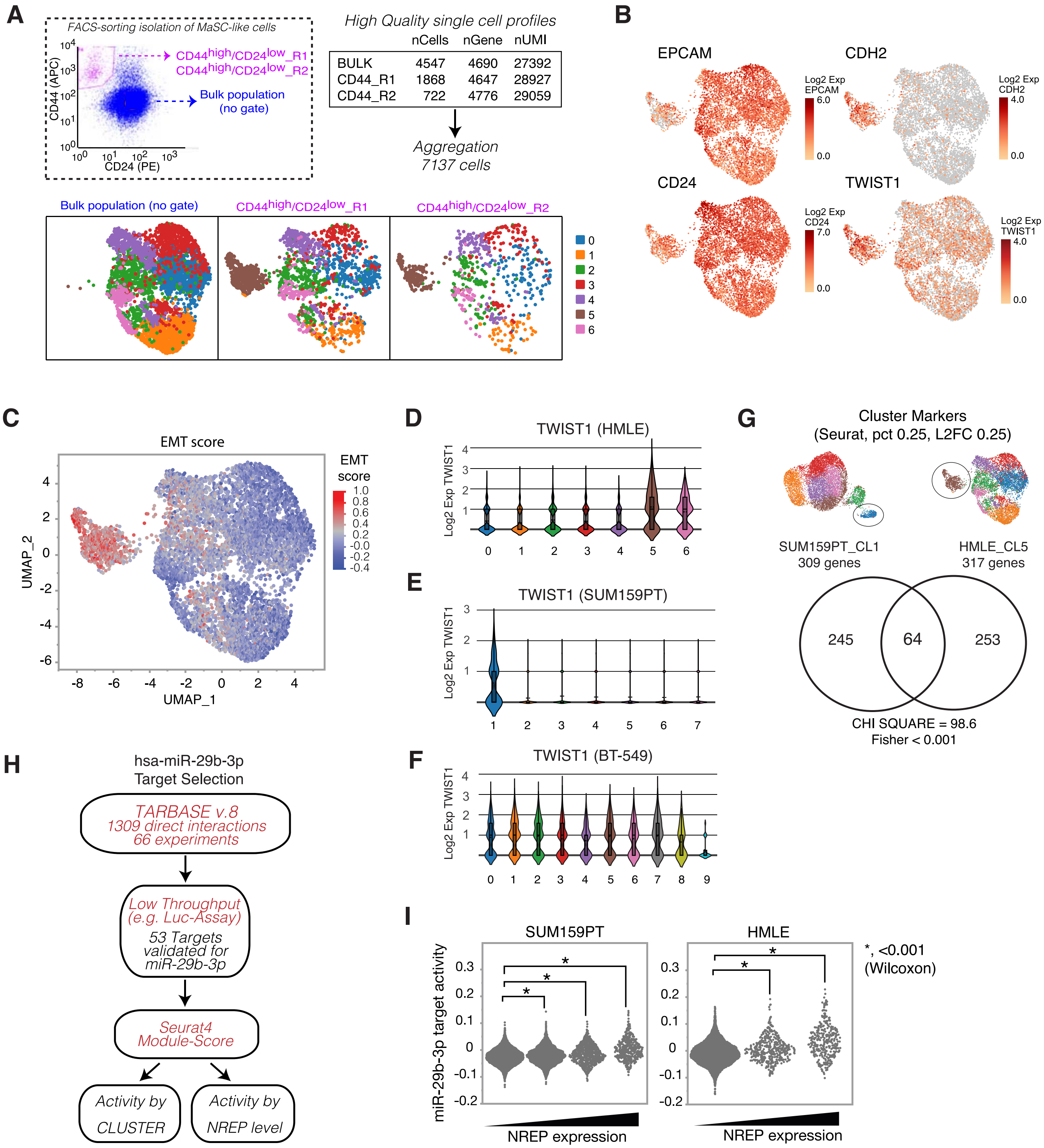
A
FACS-sorting isolation of MaSC-like cells
CD44 (APC)
CD24 (PE)
CD44high/CD24low_R1
CD44high/CD24low_R2
Bulk population (no gate)
High Quality single cell profiles
nCells nGene nUMI
BULK 4547 4690 27392
CD44_R1 1868 4647 28927
CD44_R2 722 4776 29059
Aggregation
7137 cells
Bulk population (no gate)
CD44high/CD24low_R1
CD44high/CD24low_R2
B
EPCAM
CDH2
CD24
TWIST1
Log2 Exp
C
EMT score
UMAP_1
UMAP_2
D
TWIST1 (HMLE)
Log2 Exp TWIST1
E
TWIST1 (SUM159PT)
F
TWIST1 (BT-549)
G
Cluster Markers
(Seurat, pct 0.25, L2FC 0.25)
SUM159PT_CL1
309 genes
HMLE_CL5
317 genes
245
64
253
CHI SQUARE = 98.6
Fisher < 0.001
H
hsa-miR-29b-3p
Target Selection
TARBASE v.8
1309 direct interactions
66 experiments
Low Throughput
(e.g. Luc-Assay)
53 Targets
validated for
miR-29b-3p
Seurat4
Module-Score
Activity by
CLUSTER
Activity by
NREP level
I
SUM159PT
HMLE
miR-29b-3p target activity
*, <0.001
(Wilcoxon)
NREP expression

 © The Author(s)

◀ **Figure EV4. *NREP* controls miR-29b-3p levels and activity in CD44^{high}/CD24^{low} HMLE cells.**

(**A**) Single-cell analysis of stem-like cells (maSC-like) in HMLE. Top: schematic workflow, with representative FACS profile of CD44^{high}/CD24^{low} cells and summary of cells/features analyzed. Bottom: UMAP visualization of the aggregate dataset (7137 cells), grouped by samples and colored by clusters. Clusters were identified using standard methods (see Methods) based on transcriptional similarity within each sample; cluster numbers are arbitrary and not comparable across different cell lines. (**B**) UMAP plot of HMLE cells, colored by the expression of typical epithelial (EPCAM, CD24) or mesenchymal (CDH2 and TWIST1) markers. (**C**) UMAP plot colored by the EMT module score (see Methods). (**D**, **F**) Violin plots of TWIST1 expression across HMLE clusters (**D**), SUM159PT clusters (**E**), and BT-549 clusters (**F**). (**G**) Overlap between the mesenchymal clusters in SUM159PT and HMLE cell lines based on cluster markers. (**H**) Strategy for the definition of miR-29b-3p targets activity in single-cell gene expression profiles. (**I**) Activity of miR-29b-3p targets stratified according to different bins of NREP expression (bins are defined as mean, +1 SD, +2 SD, +3 SD; the first bin contains all cells with zero values). Statistical analysis by the Wilcoxon test, comparing each cluster versus all the others. *$p < 0.001$.

© The Author(s)

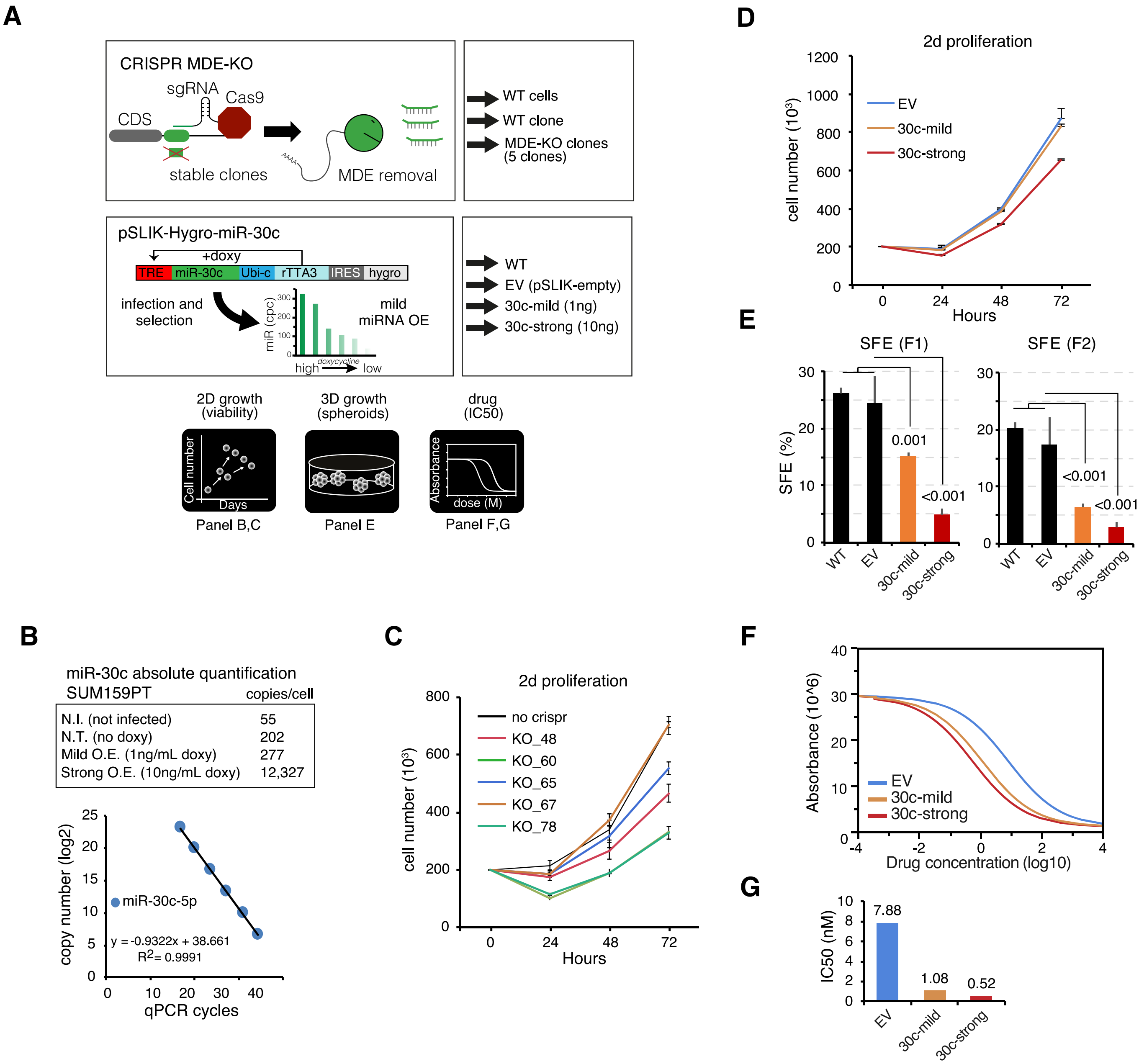

Figure EV5. Phenotypes related to SERPINE1:miR-30c-5p interaction.

(**A**) Schematic representation of the different methodological strategies used to investigate the phenotypes that depend on the SERPINE1:miR-30c-5p interaction. (**B**) Upper panel: a table summarizes miR-30c-5p levels by absolute quantification in cells infected with an inducible construct for miR-30c-5p overexpression. NI not infected cells, NT infected cells not treated with doxycycline. Lower panel: titration curve used for the absolute quantification of miR-30c-5p (see Methods). (**C**) Growth curve of SUM159PT clones bearing the MDE deletion in SERPINE1 3′UTR. $N = 3$ (average cell number and SEM). (**D**) Growth curve of SUM159PT cells upon mild or strong miR-30c-5p overexpression. $N = 3$ (average cell number and SEM). (**E**) Bar charts reporting the results of the sphere formation assay performed in SUM159PT. Shown is the sphere forming efficiency (average % and SEM from three replicate wells) measured at the first (F1) and at the second generation (F2). $N = 2$. *P* values by Student's *T*-test. (**F**, **G**) Cell viability was measured by ADP-Glo Max Assay in SUM159PT cells treated for 3 days with Paclitaxel at different concentrations (panel **F**) and used to calculate IC50 values (panel **G**). Results shown are from one of two independent experiments.

© The Author(s)
