# Supplementary material for: Canonical and non-canonical miRNA degradation shapes state transitions and stemness in breast cancer
Source: EMBO J. 2026 Aug 17;45(19):6813–46. doi: 10.1038/s44318-026-00889-8 (PMC13631368; doi:10.1038/s44318-026-00889-8)
Supplement: Supplementary file 2 [file 44318_2026_889_MOESM1_ESM.pdf]

# Appendix

## Canonical and non-canonical miRNA degradation shapes state transitions and stemness in breast cancer

Carmela Rubolino[1], Manfredi D'Onghia[1], Chiara Tordonato[2,3], Arianna Sabò[2], Alessandro D'Agnelli[1], Sara Bisi[1], Roberto Giambruno[1,4], Bianca Giuliani[1], Matteo Jacopo Marzi[1,*,†] and Francesco Nicassio[1,*,†]

[1] Center for Genomic Science of IIT@SEMM, Istituto Italiano di Tecnologia (IIT) - Via Adamello 16, 20139 Milan, Italy

[2] Department of Experimental Oncology, European Institute of Oncology (IEO) - IRCCS, Milan, Italy

[3] Department of Oncology and Hemato-Oncology, Università degli Studi di Milano, Milan, Italy

[4] Institute for Biomedical Research and Innovation, National Research Council, Palermo, Italy

* equal contribution

† to whom correspondence should be addressed. Email: francesco.nicassio@iit.it; matteo.marzi@iit.it, Tel: +39-0294375174; Fax +39-0294375991; Center for Genomic Science of IIT@SEMM, c/o Campus IFOM-IEO, Via Adamello 16, 20139, Milan, Italy.

**Table of Content**

Appendix Figure S1, page 3

Appendix Figure S2, page 5

Appendix Figure S3, page 8

Appendix Figure S4, page 10

Appendix References, page 12

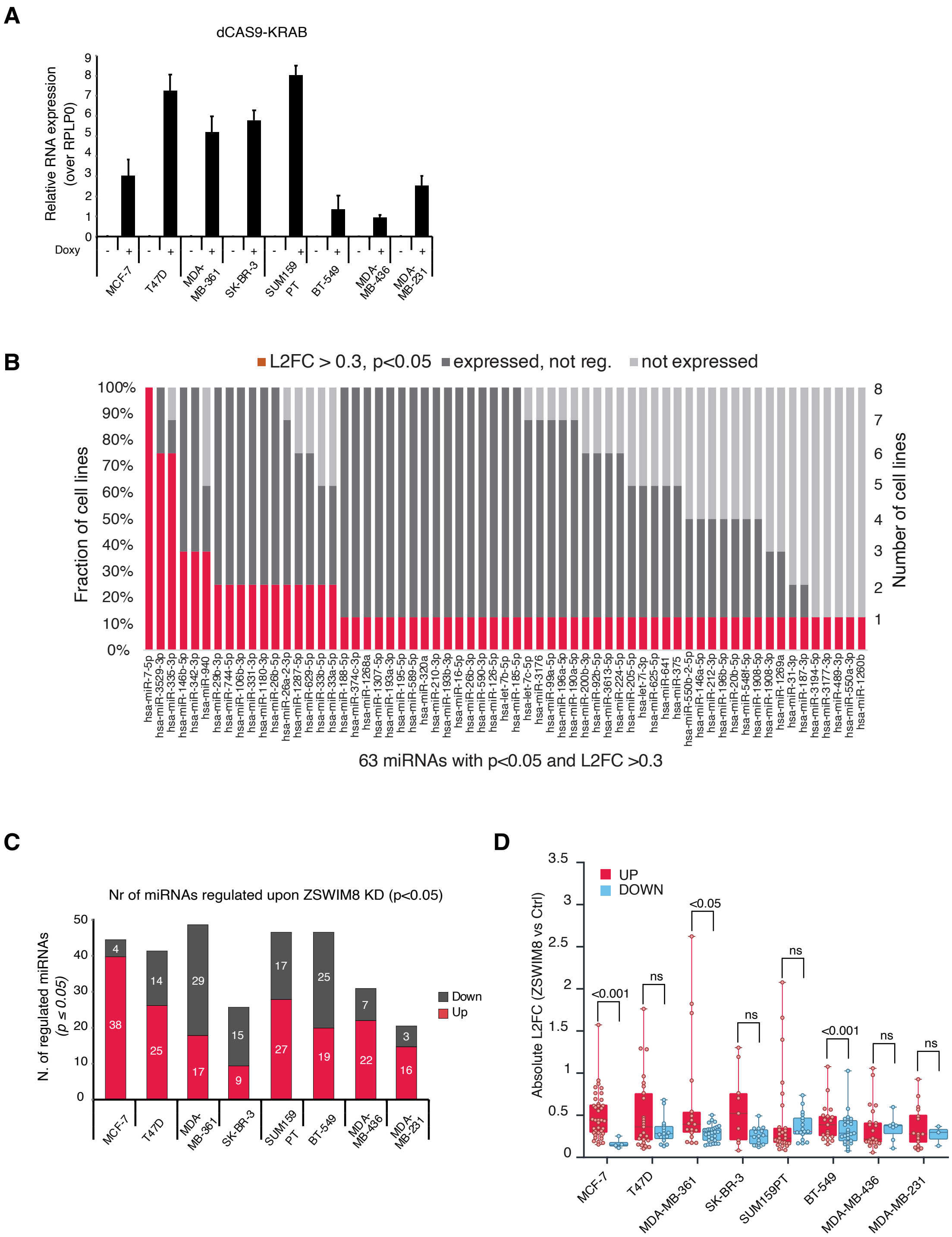
A
dCAS9-KRAB
Relative RNA expression (over RPLP0)
Doxy
MCF-7
T47D
MDA-MB-361
SK-BR-3
SUM159 PT
BT-549
MDA-MB-436
MDA-MB-231
B
L2FC > 0.3, p<0.05
expressed, not reg.
not expressed
Fraction of cell lines
Number of cell lines
hsa-miR-7-5p
hsa-miR-3529-3p
hsa-miR-335-3p
hsa-miR-146b-5p
hsa-miR-342-3p
hsa-miR-940
hsa-miR-29b-3p
hsa-miR-744-5p
hsa-miR-106b-3p
hsa-miR-331-3p
hsa-miR-1180-3p
hsa-miR-26b-5p
hsa-miR-26a-2-3p
hsa-miR-1287-5p
hsa-miR-629-5p
hsa-miR-33b-5p
hsa-miR-33a-5p
hsa-miR-188-5p
hsa-miR-374c-3p
hsa-miR-1268a
hsa-miR-1307-5p
hsa-miR-193a-3p
hsa-miR-195-5p
hsa-miR-589-5p
hsa-miR-320a
hsa-miR-210-3p
hsa-miR-193b-3p
hsa-miR-16-5p
hsa-miR-26b-3p
hsa-miR-590-3p
hsa-miR-126-5p
hsa-let-7b-5p
hsa-miR-185-5p
hsa-let-7c-5p
hsa-miR-3176
hsa-miR-99a-5p
hsa-miR-196a-5p
hsa-miR-190a-5p
hsa-miR-200b-3p
hsa-miR-92b-5p
hsa-miR-3613-5p
hsa-miR-224-5p
hsa-miR-205-5p
hsa-let-7i-3p
hsa-miR-625-5p
hsa-miR-641
hsa-miR-375
hsa-miR-550b-2-5p
hsa-miR-146a-5p
hsa-miR-212-3p
hsa-miR-196b-5p
hsa-miR-20b-5p
hsa-miR-548f-5p
hsa-miR-1908-5p
hsa-miR-1908-3p
hsa-miR-1269a
hsa-miR-31-3p
hsa-miR-187-3p
hsa-miR-3194-5p
hsa-miR-3177-3p
hsa-miR-489-3p
hsa-miR-550a-3p
hsa-miR-1260b
63 miRNAs with p<0.05 and L2FC >0.3
C
Nr of miRNAs regulated upon ZSWIM8 KD (p<0.05)
N. of regulated miRNAs (p ≤ 0.05)
Down
Up
D
UP
DOWN
Absolute L2FC (ZSWIM8 vs Ctrl)
<0.001
ns
<0.05
ns
ns
<0.001
ns
ns
MCF-7
T47D
MDA-MB-361
SK-BR-3
SUM159PT
BT-549
MDA-MB-436
MDA-MB-231

**Appendix Figure S1: Analysis of miRNAs regulated upon ZSWIM8 KD.**

(**A**) dCas9-KRAB expression in breast cancer cell lines. RT-qPCR was performed upon administration of 1 µg/ml of Doxycycline (+) for 72h. The bar chart shows the relative RNA expression (n=3; +/- st.dev) using RPLP0 as reference. (**B**) The stacked bar chart reports the number of cell lines (and the proportion) in which each of the 63 TDMD miRNA substrates was found as upregulated (red, L2FC >0.3, pvalue <0.05), expressed but not upregulated (dark grey, >10 CPM) or not expressed (light grey, <10 CPM). MiRNAs are ordered according to the number of lines where they were found as upregulated. (**C**) Number of significantly upregulated (red) and downregulated (grey) miRNAs (ZSWIM8 KD vs Ctrl) in every cell line (Student's t-test, p ≤ 0.05). (**D**) Effect size of the regulation of miRNAs upon ZSWIM8 KD. The boxplots show the L2FC values of upregulated (red) and downregulated (grey) miRNAs in every cell line (Mann-Whitney nonparametric t-test, *** p ≤ 0.001; * p≤0.05).

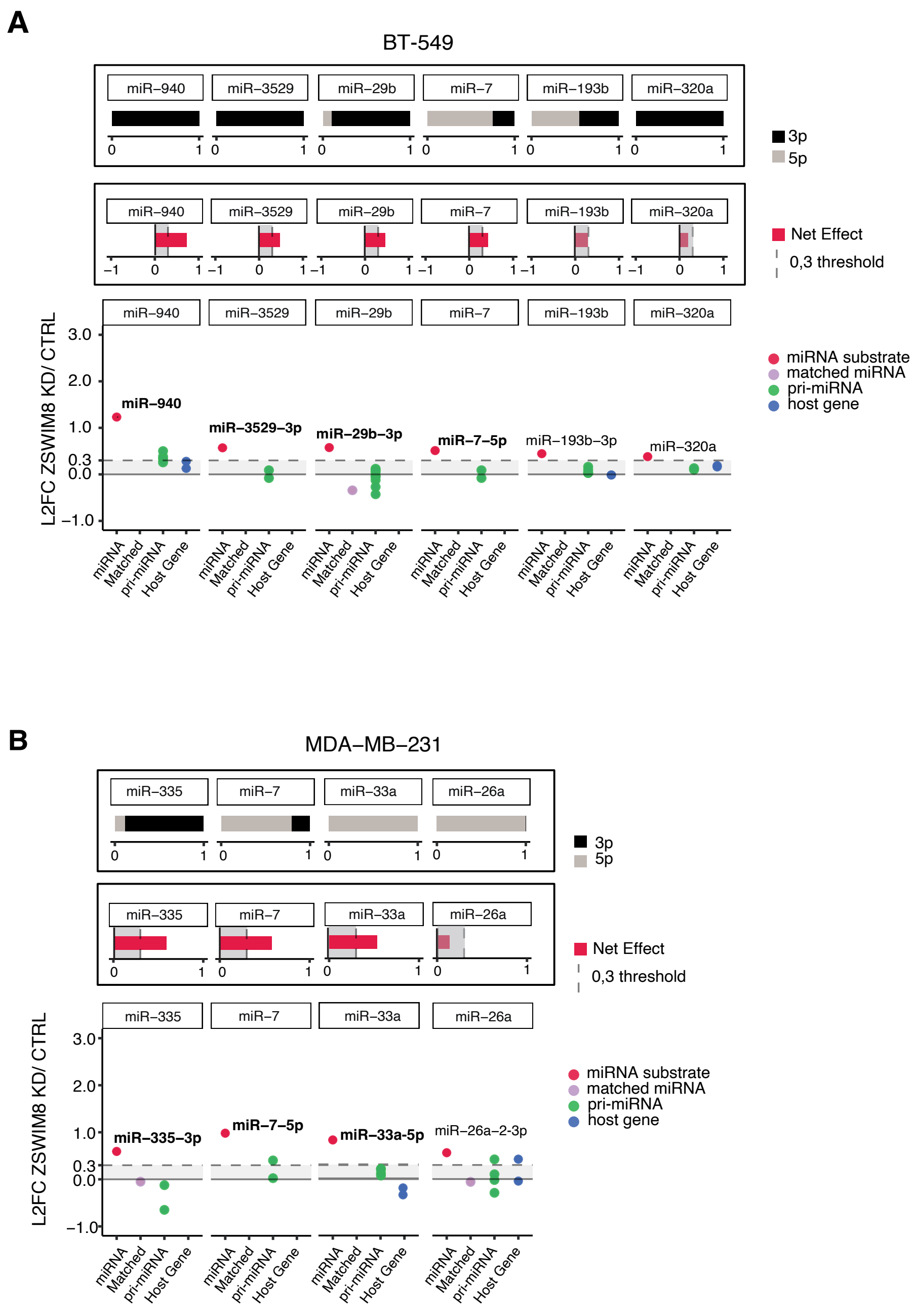
A
BT-549
miR–940
miR–3529
miR–29b
miR–7
miR–193b
miR–320a
3p
5p
Net Effect
0,3 threshold
L2FC ZSWIM8 KD/ CTRL
miR–940
miR–3529–3p
miR–29b–3p
miR–7–5p
miR–193b–3p
miR–320a
miRNA substrate
matched miRNA
pri-miRNA
host gene
miRNA
Matched
pri-miRNA
Host Gene
B
MDA–MB–231
miR–335
miR–7
miR–33a
miR–26a
3p
5p
Net Effect
0,3 threshold
L2FC ZSWIM8 KD/ CTRL
miR–335–3p
miR–7–5p
miR–33a-5p
miR–26a–2-3p
miRNA substrate
matched miRNA
pri-miRNA
host gene
miRNA
Matched
pri-miRNA
Host Gene

C

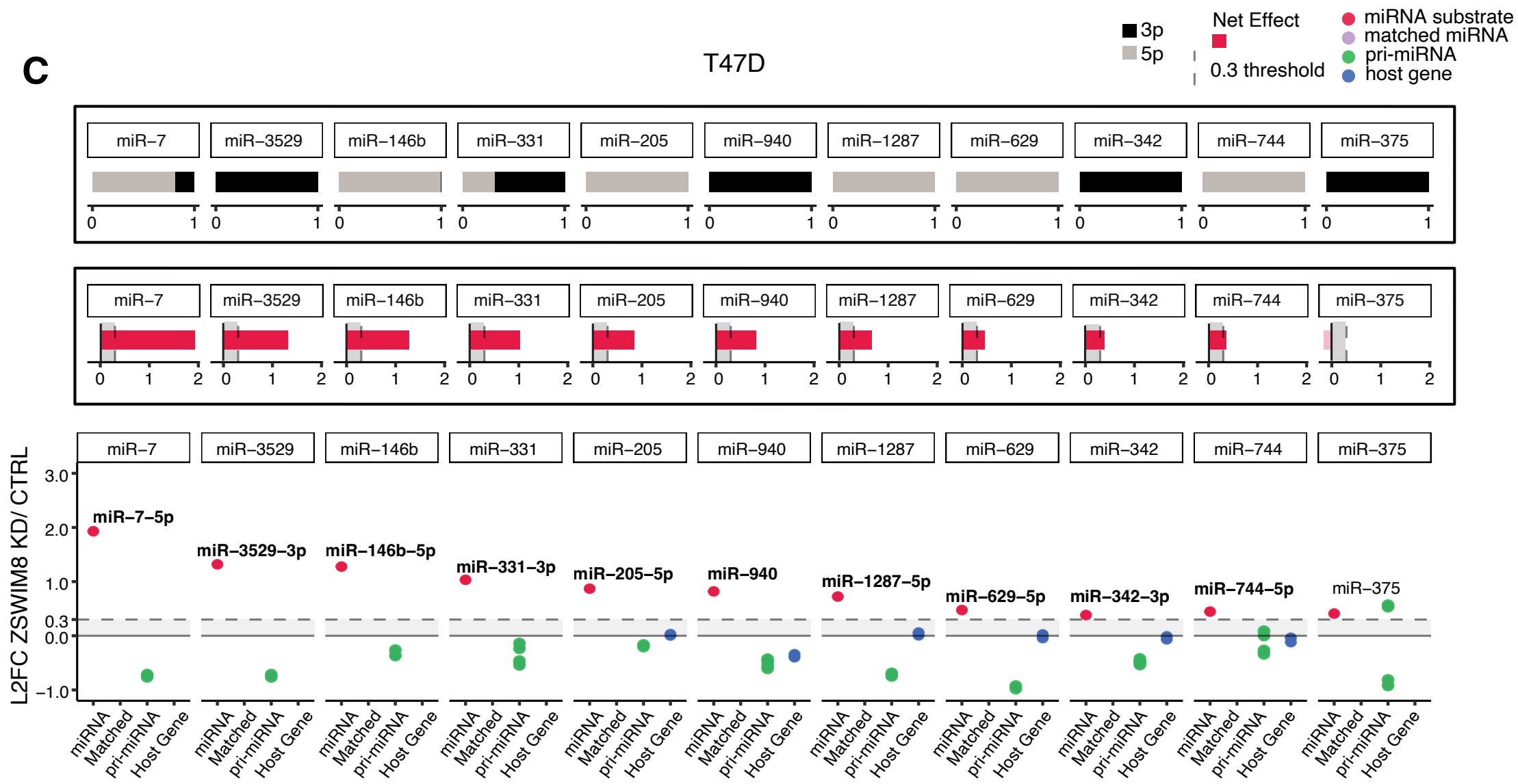
T47D
3p
5p
Net Effect
0.3 threshold
miRNA substrate
matched miRNA
pri-miRNA
host gene
miR−7
miR−3529
miR−146b
miR−331
miR−205
miR−940
miR−1287
miR−629
miR−342
miR−744
miR−375
L2FC ZSWIM8 KD/ CTRL
miR−7−5p
miR−3529−3p
miR−146b−5p
miR−331−3p
miR−205−5p
miR−940
miR−1287−5p
miR−629−5p
miR−342−3p
miR−744−5p
miR−375
miRNA
Matched
pri-miRNA
Host Gene

D

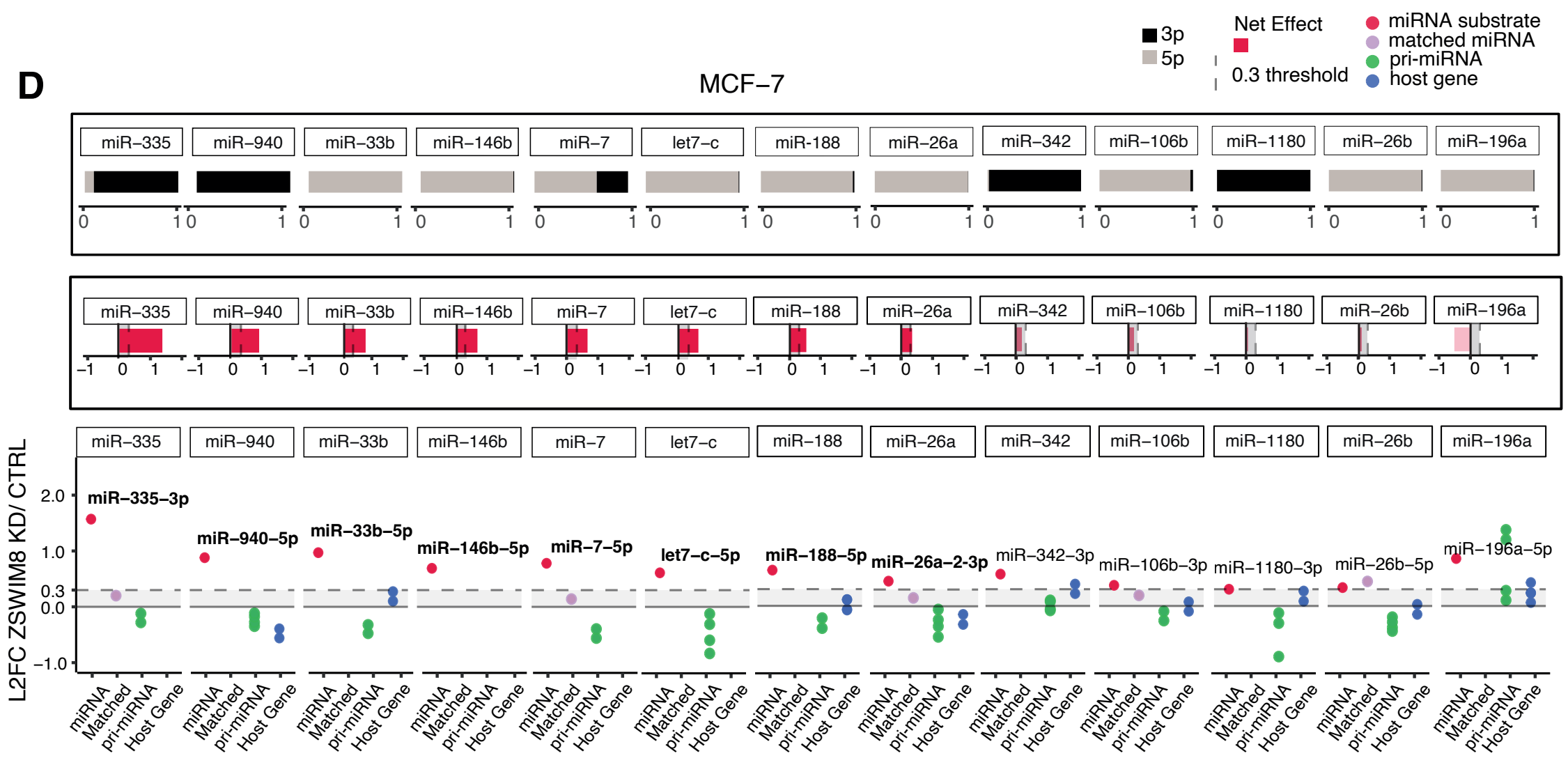
MCF−7
3p
5p
Net Effect
0.3 threshold
miRNA substrate
matched miRNA
pri-miRNA
host gene
miR−335
miR−940
miR−33b
miR−146b
miR−7
let7−c
miR−188
miR−26a
miR−342
miR−106b
miR−1180
miR−26b
miR−196a
L2FC ZSWIM8 KD/ CTRL
miR−335−3p
miR−940−5p
miR−33b−5p
miR−146b−5p
miR−7−5p
let7−c−5p
miR−188−5p
miR−26a−2−3p
miR−342−3p
miR−106b−3p
miR−1180−3p
miR−26b−5p
miR−196a−5p
miRNA
Matched
pri-miRNA
Host Gene

**Appendix Figure S2: Identification of high confidence TDMD miRNA substrates.**

(**A-D**) For each miRNA found as upregulated upon ZSWIM8 KD (see **Fig 2E**) is reported the distribution of reads from the 5p/3p strands, the TDMD net effect (red bar) and the values (L2FC values upon ZSWIM8-KD) of miRNA substrate (red dots) and matched miRNA (pink) obtained by smallRNA-Seq analysis, together with their pri-miRNAs (green), and host genes (blue dots) obtained by RT-qPCR, normalized with either RPLP0 or GADPH (two or more dots are shown, considering the two normalizations and different pair of primers used). A dashed bar indicates the threshold for the "TDMD net effect" (≥ 0.3 L2FC).

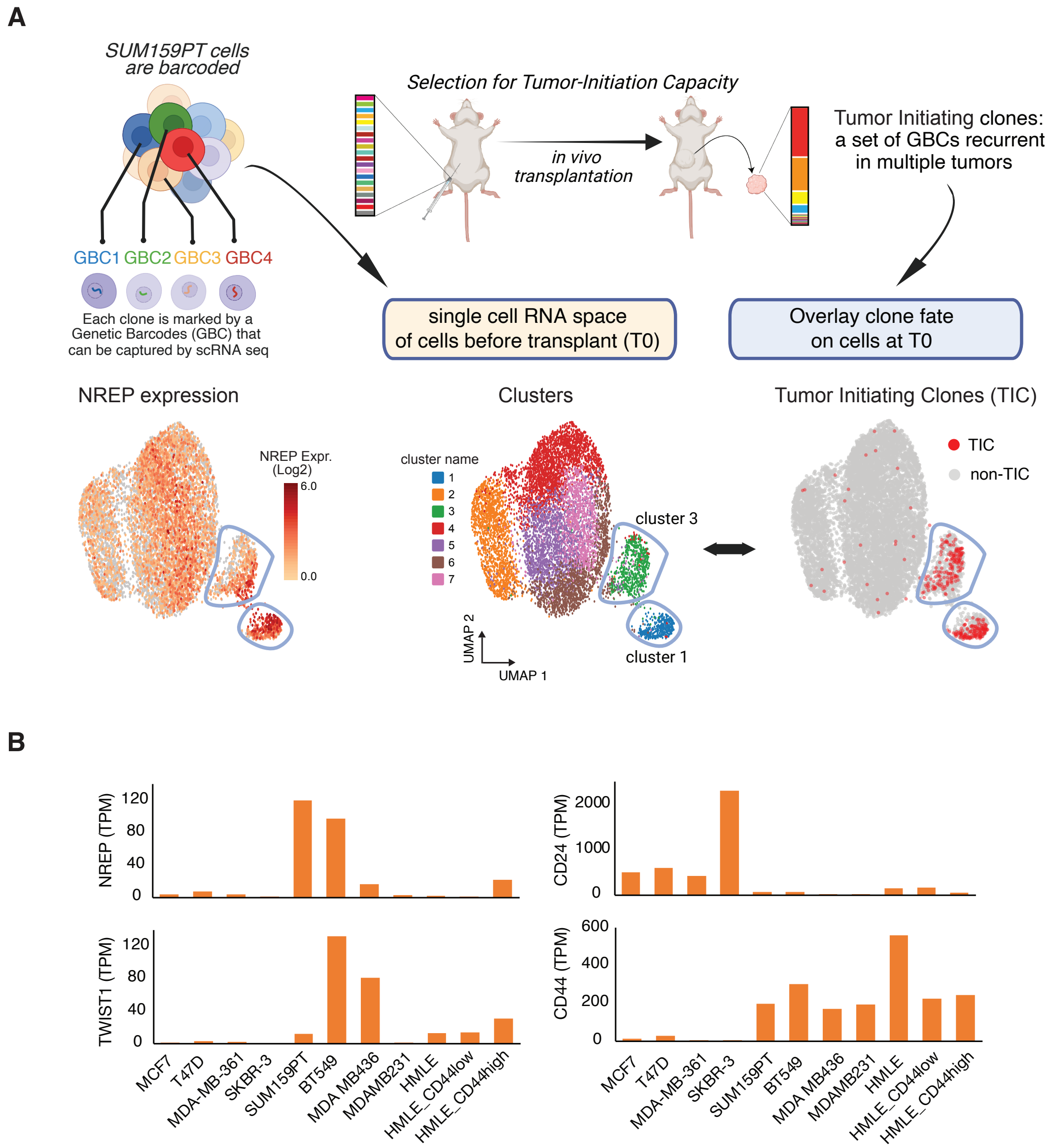
A
SUM159PT cells are barcoded
GBC1 GBC2 GBC3 GBC4
Each clone is marked by a Genetic Barcodes (GBC) that can be captured by scRNA seq
Selection for Tumor-Initiation Capacity
in vivo transplantation
Tumor Initiating clones: a set of GBCs recurrent in multiple tumors
single cell RNA space of cells before transplant (T0)
Overlay clone fate on cells at T0
NREP expression
NREP Expr. (Log2)
6.0
0.0
Clusters
cluster name
1
2
3
4
5
6
7
cluster 3
cluster 1
UMAP 2
UMAP 1
Tumor Initiating Clones (TIC)
TIC
non-TIC
B
NREP (TPM)
TWIST1 (TPM)
CD24 (TPM)
CD44 (TPM)
MCF7
T47D
MDA-MB-361
SKBR-3
SUM159PT
BT549
MDA MB436
MDAMB231
HMLE
HMLE_CD44low
HMLE_CD44high

**Appendix Figure S3: Identification of tumor initiating cells in SUM159PT cells**

(**A**) Schematics of the experimental design for the identification of tumor initiation clones in SUM159PT. Barcoded SUM159PT cells were injected orthotopically in NSG mice; after tumor formation, clonal identity and frequency were evaluated by retrieving GBC from gDNA. Barcodes recurrently found in multiple mice were defined as "tumor initiating clones" (TICs). TICs were then mapped on the different subpopulations defined by single cell RNA sequencing at parental state (T0). The two subpopulations that were strongly enriched for TICs (cluster 1 and cluster 3) display high expression of NREP. The figure is adapted from Nadalin et al, 2024. (**B**) Expression of NREP, TWIST1, CD24 and CD44 across BC cell lines and HMLE, either bulk or sorted CD44high/CD24low and CD44low/CD24high populations (TPM, by RNA sequencing).

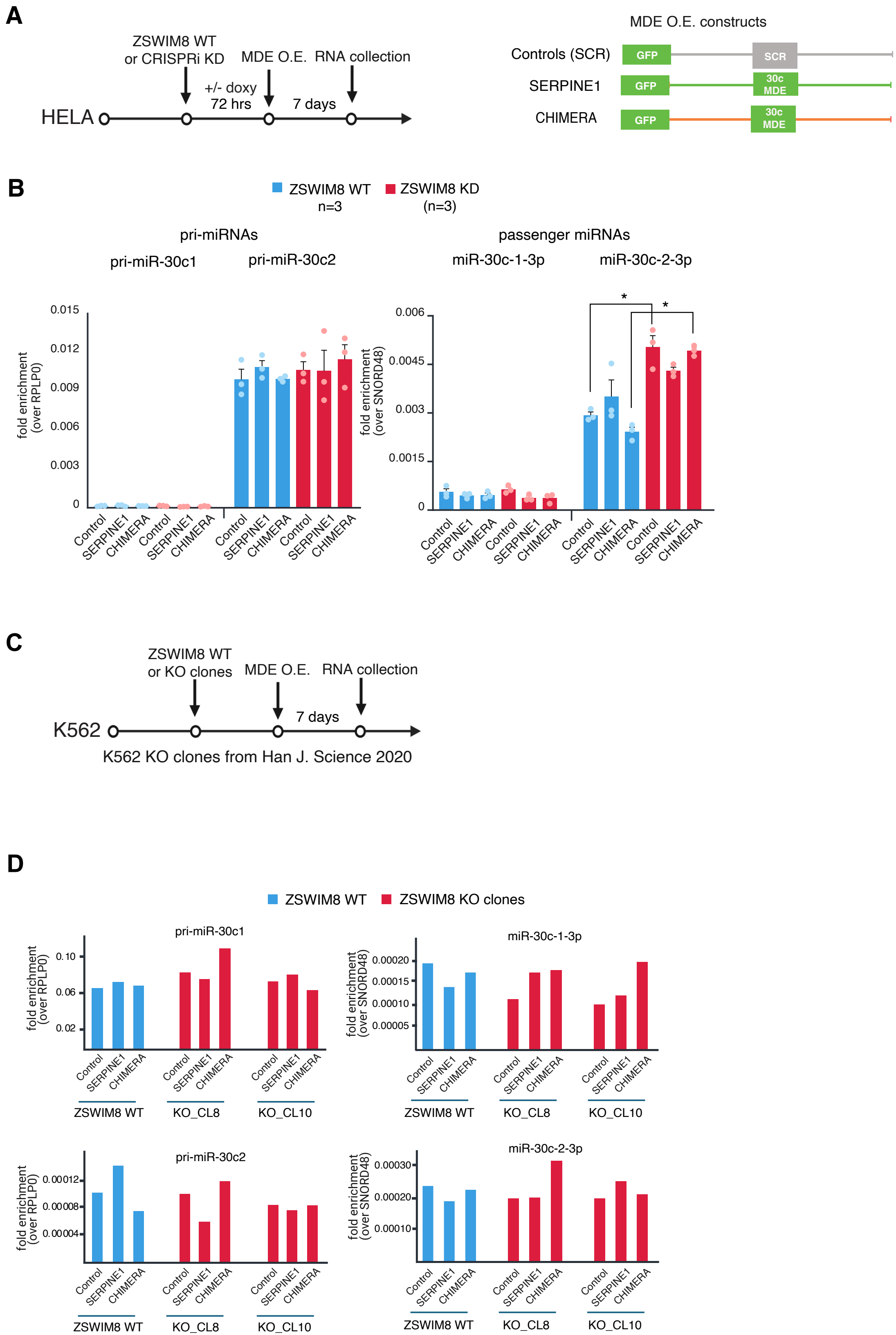

A
ZSWIM8 WT
or CRISPRi KD
MDE O.E.
RNA collection
+/- doxy
72 hrs
7 days
HELA
MDE O.E. constructs
Controls (SCR)
SERPINE1
CHIMERA
GFP
SCR
30c MDE
B
ZSWIM8 WT n=3
ZSWIM8 KD (n=3)
pri-miRNAs
pri-miR-30c1
pri-miR-30c2
passenger miRNAs
miR-30c-1-3p
miR-30c-2-3p
fold enrichment (over RPLP0)
fold enrichment (over SNORD48)
Control
SERPINE1
CHIMERA
C
ZSWIM8 WT
or KO clones
MDE O.E.
RNA collection
7 days
K562
K562 KO clones from Han J. Science 2020
D
ZSWIM8 WT
ZSWIM8 KO clones
pri-miR-30c1
miR-30c-1-3p
pri-miR-30c2
miR-30c-2-3p
ZSWIM8 WT
KO_CL8
KO_CL10

**Appendix Figure S4: Post-transcriptional and ZSWIM8 independent TDMD mechanism**

(**A**) Schematic overview of the experimental strategy used in HeLa used to assess ZSWIM8 dependent and -independent TDMD mechanism. Cells were infected with sgRNA targeting ZSWIM8 (sg28, sg34, sg42), then treated or not with doxycycline to induce ZSWIM8 knockdown (WT vs KD) and finally transduced with different TDMD constructs. Samples were collected after 7 days for the analyses. Shown also the TDMD constructs that were used. (**B**) Expression levels of pri-miRNAs and passenger strands for miR-30c-5p were measured by RT-qPCR in HeLa cells. Data are normalized to *RPLP0* (for pri-miRNAs) and *SNORD48* (for passenger strands). Bar charts show the average and SEM (N=3). P-values were calculated through Student's t-test comparing WT and ZSWIM8 KD cells expressing the same TDMD construct. The only significant effect observed was an increase (rather than a decrease) in miR-30c-2-3p levels upon ZSWIM8 KD. * = $p < 0.05$. (**C**) Schematic of the experimental strategy used in K562 cells to assess ZSWIM8-dependent and -independent TDMDs, comparing wild-type or ZSWIM8 knock-out clones. (**D**) Expression levels of pri-miRNAs and passenger strands were measured by RT-qPCR in K562 WT or KO clones (KO_CL8 and KO_CL10) upon over-expression of the same TDMD constructs used in panel A and B. Bar charts report the expression levels normalized over *RPLP0* (for pri-miRNAs) and *SNORD48* (for passenger strands) for individual replicates.

## Appendix References

Nadalin F, Marzi MJ, Pirra Piscazzi M, Fuentes-Bravo P, Procaccia S, Climent M, Bonetti P, Rubolino C, Giuliani B, Papatheodorou I *et al* (2024) Multi-omic lineage tracing predicts the transcriptional, epigenetic and genetic determinants of cancer evolution. *Nat Commun* 15: 7609
