# Supplementary material for: Canonical and non-canonical miRNA degradation shapes state transitions and stemness in breast cancer
Source: EMBO J. 2026 Aug 17;45(19):6813–46. doi: 10.1038/s44318-026-00889-8 (PMC13631368; doi:10.1038/s44318-026-00889-8)
Supplement: Supplementary file 3 [file 44318_2026_889_MOESM2_ESM.pdf]

THE EMBO JOURNAL

Canonical and non-canonical miRNA degradation shapes state transitions and stemness in breast cancer

Carmela Rubolino, Manfredi D'Onghia, Chiara Tordonato, Arianna Sabò, Alessandro D'Agnelli, Sara Bisi, Roberto Giambruno, Bianca Giuliani, Matteo Marzi, and Francesco Nicassio

Corresponding authors: Francesco Nicassio (francesco.nicassio@iit.it) , Matteo Marzi (matteo.marzi@iit.it)

Review Timeline:

Submission Date:	19th Sep 25
Editorial Decision:	13th Nov 25
Revision Received:	9th Mar 26
Editorial Decision:	25th May 26
Editor's Correspondence:	2nd Jun 26
Revision Received:	3rd Jul 26
Accepted:	22nd Jul 26

Editor: Daniel Klimmeck

Transaction Report:

(Note: With the exception of the correction of typographical or spelling errors that could be a source of ambiguity, letters and reports are not edited. Depending on transfer agreements, referee reports obtained elsewhere may or may not be included in this compilation. Referee reports are anonymous unless the Referee chooses to sign their reports.)

Dear Dr Nicassio,

Thank you again for the submission of your manuscript (EMBOJ-2025-122495) to The EMBO Journal, as well as for your patience with our feedback at this time. As mentioned earlier, your study was assessed by three reviewers with expertise in cancer biology and RNA research, whose comments are enclosed below.

As you will see from the experts' reports, the referees acknowledge the analysis and potential interest and value of your findings. However, they also express a number of important issues which need to be addressed thoroughly to make them supportive of publication in the EMBO Journal.
In more detail, referee #1 states that the findings are too preliminary and mechanistic detail provided on miRNA mode of action specification and the non-canonical ZSWIM8-independent pathway remains too limited (ref#1, pts.4, 7). This referee also points out to major gaps in the analysis regarding pathophysiological relevance of your findings (ref#1, pts.5,6). Further, the experts raise a number of issues related to the additional controls required, data presentation and statistics applied, as well as overall discussion of related literature, that would need to be conclusively addressed to achieve the level of robustness and clarity needed for The EMBO Journal.

Given the overall interest stated and broader angle of your findings, we are able to invite you to revise your manuscript experimentally to address the referees' comments. However, please note that the extent of revisions requested appear threshold in our view for the amount of complementary work we typically invite for our venue; also, I need to stress that we do require strong support from the referees on a revised version of the study in order to move on to publication of the work.

I would appreciate if you could contact me during the next weeks for exchange e.g. a video call to discuss your perspective on the comments and potential plan for revisions.

We generally allow three months as standard revision time. As a matter of policy, competing manuscripts published during this period will not negatively impact on our assessment of the conceptual advance presented by your study. However, we request that you contact the editor as soon as possible upon publication of any related work, to discuss how to proceed. Should you foresee a problem in meeting this three-month deadline, please let us know in advance and we may be able to grant an extension.

When submitting your revised manuscript, please carefully review the instructions below.

Please feel free to approach me any time should you have additional questions related to this.

Thank you for the opportunity to consider your work for publication.

I look forward to your revision.

Best regards,

Daniel Klimmeck

Daniel Klimmeck, PhD
Senior Editor
The EMBO Journal

Instruction for the preparation of your revised manuscript:

1) a .docx formatted version of the manuscript text (including legends for main figures, EV figures and tables). Please make sure that the changes are highlighted to be clearly visible.

2) individual production quality figure files as .eps, .tif, .jpg (one file per figure).

3) a .docx formatted letter INCLUDING the reviewers' reports and your detailed point-by-point response to their comments. As part of the EMBO Press transparent editorial process, the point-by-point response is part of the Review Process File (RPF), which will be published alongside your paper.

4) a complete author checklist, which you can download from our author guidelines (https://wol-prod-cdn.literatumonline.com/pb-

assets/embo-site/Author Checklist%20-%20EMBO%20J-1561436015657.xlsx). Please insert information in the checklist that is also reflected in the manuscript. The completed author checklist will also be part of the RPF.

5) Please note that all corresponding authors are required to supply an ORCID ID for their name upon submission of a revised manuscript.

6) It is mandatory to include a 'Data Availability' section after the Materials and Methods. Before submitting your revision, primary datasets produced in this study need to be deposited in an appropriate public database, and the accession numbers and database listed under 'Data Availability'. Please remember to provide a reviewer password if the datasets are not yet public (see https://www.embopress.org/page/journal/14602075/authorguide#datadeposition).
In case you have no data that requires deposition in a public database, please state so in this section. Note that the Data Availability Section is restricted to new primary data that are part of this study.
*** Note - All links should resolve to a page where the data can be accessed. ***

7) Our journal encourages inclusion of *data citations in the reference list* to directly cite datasets that were re-used and obtained from public databases. Data citations in the article text are distinct from normal bibliographical citations and should directly link to the database records from which the data can be accessed. In the main text, data citations are formatted as follows: "Data ref: Smith et al, 2001" or "Data ref: NCBI Sequence Read Archive PRJNA342805, 2017". In the Reference list, data citations must be labeled with "[DATASET]". A data reference must provide the database name, accession number/identifiers and a resolvable link to the landing page from which the data can be accessed at the end of the reference. Further instructions are available at .

8) At EMBO Press we ask authors to provide source data for the main and EV figures. Our source data coordinator will contact you to discuss which figure panels we would need source data for and will also provide you with helpful tips on how to upload and organize the files.

Numerical data can be provided as individual .xls or .csv files (including a tab describing the data). For 'blots' or microscopy, uncropped images should be submitted (using a zip archive or a single pdf per main figure if multiple images need to be supplied for one panel). Additional information on source data and instruction on how to label the files are available at .

9) We replaced Supplementary Information with Expanded View (EV) Figures and Tables that are collapsible/expandable online (see examples in https://www.embopress.org/doi/10.15252/embj.201695874). A maximum of 5 EV Figures can be typeset. EV Figures should be cited as 'Figure EV1, Figure EV2" etc. in the text and their respective legends should be included in the main text after the legends of regular figures.

- For the figures that you do NOT wish to display as Expanded View figures, they should be bundled together with their legends in a single PDF file called *Appendix*, which should start with a short Table of Content. Appendix figures should be referred to in the main text as: "Appendix Figure S1, Appendix Figure S2" etc. See detailed instructions regarding expanded view here: .

- Additional Tables/Datasets should be labelled and referred to as Table EV1, Dataset EV1, etc. Legends have to be provided in a separate tab in case of .xls files. Alternatively, the legend can be supplied as a separate text file (README) and zipped together with the Table/Dataset file.

10) When assembling figures, please refer to our figure preparation guideline in order to ensure proper formatting and readability in print as well as on screen:
http://bit.ly/EMBOPressFigurePreparationGuideline

Please remember: Digital image enhancement is acceptable practice, as long as it accurately represents the original data and conforms to community standards. If a figure has been subjected to significant electronic manipulation, this must be noted in the figure legend or in the 'Materials and Methods' section. The editors reserve the right to request original versions of figures and the original images that were used to assemble the figure.

11) For data quantification: please specify the name of the statistical test used to generate error bars and P values, the number (n) of independent experiments (specify technical or biological replicates) underlying each data point and the test used to calculate p-values in each figure legend. The figure legends should contain a basic description of n, P and the test applied. Graphs must include a description of the bars and the error bars (s.d., s.e.m.).

Please remember: Digital image enhancement is acceptable practice, as long as it accurately represents the original data and conforms to community standards. If a figure has been subjected to significant electronic manipulation, this must be noted in the figure legend or in the 'Materials and Methods' section. The editors reserve the right to request original versions of figures and the original images that were used to assemble the figure.

Further information is available in our Guide For Authors: https://www.embopress.org/page/journal/14602075/authorguide

We realize that it is difficult to revise to a specific deadline. In the interest of protecting the conceptual advance provided by the work, we recommend a revision within 3 months (10th Feb 2026). Please discuss the revision progress ahead of this time with the editor if you require more time to complete the revisions.

Referee #1:

This manuscript makes four main claims: (1) that TDMD plays a role in human cancer; (2) that knockdown of ZSWIM8 in a series of cultured breast cancer cell lines reveals multiple miRNA-TDMD trigger pairs, most specific to a single cell line; (3) that TDMD is linked to transcriptional plasticity and tumor heterogeneity, and (4) that miRNAs can be degraded by a ZSWIM8-independent TDMD mechanism. Each of these claims could easily take an entire paper to establish properly. Although there are many hints of interesting discoveries in the authors' data, in its current form the manuscript is too preliminary for publication.

Major Concerns

(1) Much of the data in the paper is presented as "log2 fold-change," a metric that obscures the actual abundance of the miRNA or TDMD trigger. It is extremely rare for a low-abundance miRNA to be biologically functional in a particular cell type. Showing the actual data in a scatter plot (e.g., control vs. ZSWIM8 knockdown) would allow a reader to assess whether a miRNA whose abundance increases upon a reduction in ZSWIM8 mRNA is sufficiently abundant to be of biological interest.

(2) Student's t-test is not appropriate for determining the significance of changes in abundance measured by small RNA-seq (e.g., Fig 1D, MA plots). High-throughput sequencing data are non-parametric. Moreover, statistical significance must be corrected for multiple hypothesis testing. An uncorrected p-value of 0.05 will no longer be significant once the correction is applied. The standard method, typically using Dseq2, is to use the Wald test and correct for multiple testing using the Benjamini-Hochberg procedure.

(3) It is puzzling how tiny effect sizes, measured with just three replicates, can be significant. The situation becomes even more confusing once the authors introduce "net effect" as a metric. Given the large difference in the CDF plots for TargetScan-predicted targets vs. non-targets (e.g., Fig. 4E), it would be simpler to restrict the study to those miRNAs whose increased abundance upon ZSWIM8 knockdown has a meaningful effect on the abundance of the miRNA's targets.

(4) miR 29b-3p differs from miR 29a and miR 29c-3p by five and four nucleotides, respectively. Which of these differences contributes to the differential effect of ZSWIM8 knockdown?

(5) The authors speculate repeatedly about the connection between TDMD and oncogenesis, but present no evidence showing that when the abundance of ZSWIM8 or a specific TDMD trigger is reduced that oncogenic progression is altered.

(6) Similarly, the authors propose that TDMD plays a role in human cancer, yet all of their studies are confined to immortalized, breast cancer cell lines in vitro. To make such an assertion would require, for example, evidence correlating mutations in ZSWIM8 or a TDMD trigger to clinical cancer progression or showing a change in oncogenesis upon altering the function or abundance of ZSWIM8 or a TDMD trigger in an appropriate orthotopic mouse model.

(7) Although the finding that *SERPINE1* triggers TDMD of miR 30c-5p independently from ZSWIM8 or proteasome function is interesting, the authors identify the phenomenon, then move on to other experiments. In fact, the data for tailing and trimming of miR 30c-5p resides in a 2018 paper from these authors. The finding of ZSWIM8-independent TDMD merits an entire research paper explaining at least some of this novel mechanism.

Minor Points

(1) It is unclear why the authors introduce a new term, "matched strand," for what is normally called *passenger* or *miR** strand of a miRNA duplex.

(2) Is there an error in this sentence, "...were prioritized as high-confidence TDMD substrates based on the concordance between increased expression upon ZSWIM8 depletion and reduced binding to their targets"? Do the authors mean increased binding to their targets? Why would binding be decreased when miRNA levels increase?

(3) The term "miRNA activity" is confusing. eCLIP measures miRNA target site occupancy, not miRNA function. It would be helpful to specify which biochemical process is being discussed instead of using the vague word "activity."

(4) The figures are tiny. Once further reduced in a journal, they will become unreadable.

(5) Please indicate the number of biologically independent replicates for the various high-throughput sequencing experiments.

Referee #2:

Rubolino and colleagues study the mechanisms of TDMD across several breast cancer cell lines. They identify miRNAs regulated by TDMD and identify their triggers. The study begins broadly and then narrows its focus to two specific examples in breast cancer: NREP:miR-29b-3p and SERPINE1:miR-30. The first interaction is shown to depend on ZSWIM8, whereas the second appears ZSWIM8-independent. Overall, the experiments are well designed and well carried out. Moreover, the manuscript is well written with good visualizations, which makes it easy to follow. In summary, the manuscript, in my opinion, definitely hits the significance threshold for this journal (and other journals of even higher impact factor). However, a few questions and suggestions should be considered/clarified before publication.

Major concerns
1. In Figure 1C, individual data points and error bars are missing for the control samples. Without them, it is difficult to interpret why T47D is reported as $p < 0.05$ while showing less variability than MCF-7 ($p < 0.001$).
2. Figure 1E: Roughly 25 % (31 miRNAs) are detected in multiple cell lines. Why are the remaining miRNAs observed in only one line? Is their expression cell-line-specific?
3. Some experiments in Figure 2CD miss measurements for the 'matched miRNA' and/or 'hostgene' (the pink and blue dots). Can the authors clarify why these are missing?
4. The authors state that miR-7 interacts with LINC00632, but all peaks are located within the CDR1as exon. How can one distinguish interaction with LINC00632 from binding to the spliced CDR1as transcript? Isn't it more likely that miR-7 interacts with CDR1as directly?
5. The authors state that NREP is a potential trigger for miR-29b-3p based on TDMDfinder, but in Figure 3F NREP appears only in the control binding plot, but not in ZSWIM8-KD. In addition, the text refers to Figure 3G when arguing that COL5A1 and SPARC lack TDMD hallmarks, but that figure does not seem to support the claim.
6. For the scRNA-seq experiment in the HMLE cells, the authors sequenced two FACS-sorted replicates and one 'bulk'. If I understand it correctly, the brown cluster in Figure S5A represents the sorted population. If so, why do the FACS-sorted replicates still contain so many other cells? Could this be explained by imperfect sorting, or did the authors use any integration approach in Seurat that forces cluster alignment?
Minor concerns
7. ZSWIM8 is misspelled once as "ZWSIM8" at the end of the introduction.
8. The legend for Figure 7C is missing and should be added.

Referee #3:

In this manuscript, Nicassio and colleagues study the process of target-directed miRNA decay (TDMD) in breast cancer cells. TDMD has been described relatively recently, and it is arguably one of the most timely and active lines of research into RNA biology. While the main mechanism of TDMD via ZSWIM8-mediated AGO proteosomal degradation has been recently described, the propensity of TDMD in different physiological systems, the nature of TDMD triggers, and the biological functions of individual TDMD interactions between miRNAs and their targets are all only partially understood. The manuscript describes very rigorous work with important progress on all three of these, and as such, it is an excellent fit for The EMBO Journal. The manuscript is very well written and the data are presented mostly clearly. The TDMD targets identified in the various breast cancer cell lines will be an excellent resource for further study; also, the overall modest increases in miRNA levels observed with a few clear exceptions put important bounds on TDMD relevance. The characterization of miR-33 triggers will also open interesting research directions, as the potential within-pathway feedback loop is very interesting. The ZSWIM-independent SERPINE1-miR-30 axis will also be very interesting to study further out of scope of this study.

I have only minor suggestions before recommending to publish:
1. In Figure 2D, it is not clear enough what is going on, in particular in the 2nd box with the net effects. Adding more boundaries (e.g., a y axis) or making the figure larger will make it easier to understand.
2. In Figure 4O - The other genes in the region beside NREP should be shown.
3. Figure 6- Can the fold changes in miR-30 be compared to the fold changes induced by ZSWIM8-mediated decay? In particular how it compares to the net effect threshold of 0.3 used in the previous parts of the manuscript?

Referee #1:

This manuscript makes four main claims: (1) that TDMD plays a role in human cancer; (2) that knockdown of ZSWIM8 in a series of cultured breast cancer cell lines reveals multiple miRNA-TDMD trigger pairs, most specific to a single cell line; (3) that TDMD is linked to transcriptional plasticity and tumor heterogeneity, and (4) that miRNAs can be degraded by a ZSWIM8-independent TDMD mechanism. Each of these claims could easily take an entire paper to establish properly. Although there are many hints of interesting discoveries in the authors' data, in its current form the manuscript is too preliminary for publication.

Major Concerns

R1.Q1. *Much of the data in the paper is presented as "log2 fold-change," a metric that obscures the actual abundance of the miRNA or TDMD trigger. It is extremely rare for a low-abundance miRNA to be biologically functional in a particular cell type. Showing the actual data in a scatter plot (e.g., control vs. ZSWIM8 knockdown) would allow a reader to assess whether a miRNA whose abundance increases upon a reduction in ZSWIM8 mRNA is sufficiently abundant to be of biological interest.*

R1.R1. We agree with the reviewer that evaluating TDMD events based solely on log2 fold-change (L2FC) can be misleading, and that absolute miRNA abundance is a key parameter to assess potential biological relevance. For this reason, miRNA abundance is explicitly taken into account throughout the analysis. In **Figure 1D**, miRNAs are displayed using MA plots in which the x-axis reports the average miRNA expression (RPM) in control samples, allowing direct visualization of the basal abundance on which ZSWIM8 depletion acts. Importantly, miRNAs expressed below a defined threshold (RPM <10 in ZSWIM perturbed cells) were excluded from downstream analyses, even when showing significant fold changes, as very low-abundance miRNAs are unlikely to be functionally relevant. This cutoff restricts the analysis to approximately the ~200 most abundantly expressed miRNAs per cell line, excluding poorly expressed species. In addition, downstream analyses focus on miRNAs that show endogenous target occupancy by miR-eCLIP, providing independent biochemical evidence that these miRNAs engage their targets at physiological expression levels. Together, these criteria ensure that the identified TDMD substrates are supported both by abundance-aware expression data and by direct evidence of miRNA–target interaction.

To clarify this point, we revised the Methods section and the **Figure 1D** legend, updated the x-axis label and added a dashed vertical line marking the RPM>10 cutoff.

R1.Q2. *Student's t-test is not appropriate for determining the significance of changes in abundance measured by small RNA-seq (e.g., Fig 1D, MA plots). High-throughput sequencing data are non-parametric. Moreover, statistical significance must be corrected for multiple hypothesis testing. An uncorrected p-value of 0.05 will no longer be significant once the correction is applied. The standard method, typically using Dseq2, is to use the Wald test and correct for multiple testing using the Benjamini-Hochberg procedure.*

R1.R2. We agree that high-throughput sequencing data require appropriate statistical modelling and correction for multiple hypothesis testing, although Student's t-test has been used before for miRNA-seq, where reads capture the entire gene level information and the number of expressed species is usually in the mid-low range (Simeone et al., NAR 2022). To provide a more robust statistical framework, in the revised manuscript, we re-analysed the small RNA-seq datasets using both DESeq2 and EdgeR, applying the Wald test (DESeq2) or the quasi-likelihood F-test (EdgeR) and correcting for multiple testing using the Benjamini–Hochberg procedure. The results are now reported in **Figure 1D**, **Figure 1E** and **Dataset EV1**. In the updated **Figure 1D**, candidate TDMD substrates were selected only if L2FC >0.3 and are now annotated according to their FDR-adjusted significance, using thresholds of FDR <0.05 and FDR <0.2. This allows the reader to immediately distinguish high-confidence events from those supported by a more relaxed threshold across the different cellular models. The **Results** and **Discussion** sections were updated accordingly in the revised manuscript.

An updated snapshot of **Figure 1D** is provided below (shown on the left) to illustrate how statistical support is now explicitly reported compared to the previous version (shown on the right).

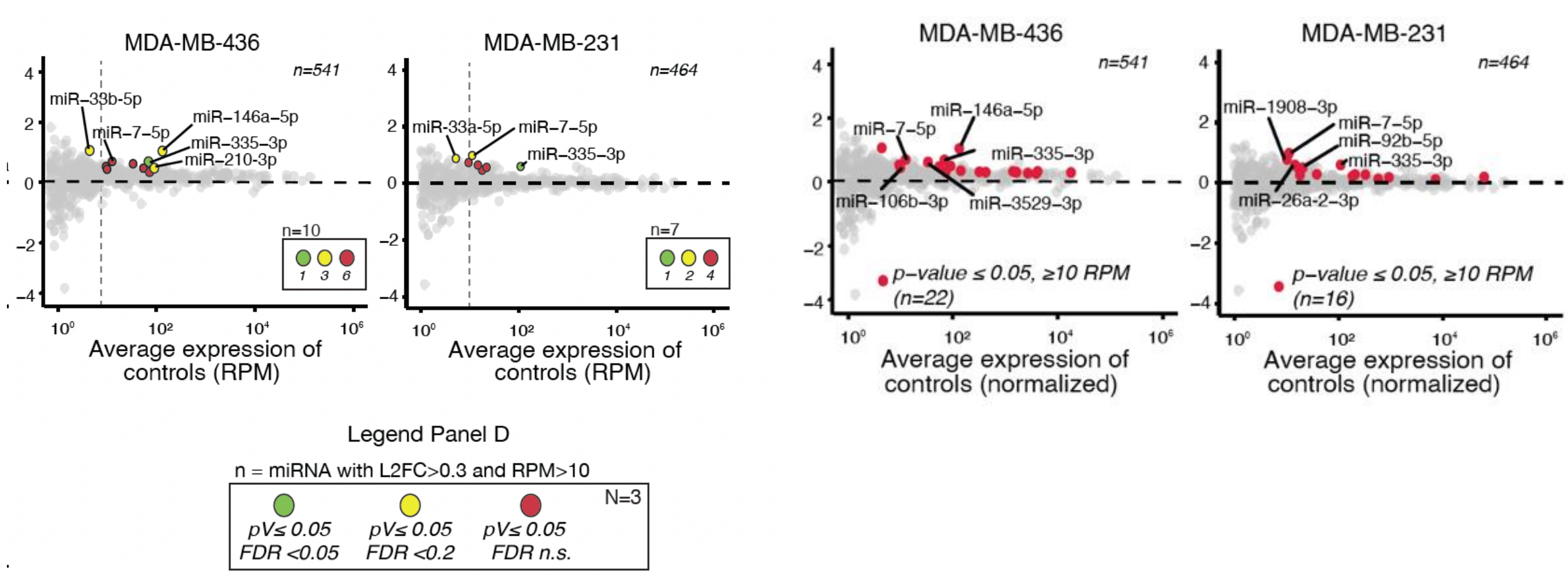

R1.Q3. *It is puzzling how tiny effect sizes, measured with just three replicates, can be significant. The situation becomes even more confusing once the authors introduce "net effect" as a metric. Given the large difference in the CDF plots for TargetScan-predicted targets vs. non-targets (e.g., Fig. 4E), it would be simpler to restrict the study to those miRNAs whose increased abundance upon ZSWIM8 knockdown has a meaningful effect on the abundance of the miRNA's targets.*

R1.R3. We understand the reviewer's concern regarding the magnitude of the observed effects and the use of the "net effect" metric. TDMD is not expected to generate large, binary changes in steady-state miRNA levels. Rather, it contributes quantitatively to miRNA turnover, and moderate increases upon ZSWIM8 depletion are consistent with previous reports and with the notion that TDMD acts as a modulatory layer of post-transcriptional control. The "net effect" metric was introduced precisely because the overall changes are modest. When fold changes are limited in magnitude, it becomes essential to verify that the observed regulation is genuinely post-transcriptional and not driven by altered transcription or precursor processing. By subtracting the transcriptional component (assessed via pri-miRNA and host gene measurements), the net effect isolates the post-transcriptional contribution attributable to TDMD. This step increases the stringency of the analysis by eliminating potential confounding factors, rather than inflating small effects. We updated the manuscript in **Results** section to clarify the rationale and implementation of the "net effect".

With respect to the suggestion to restrict the analysis to miRNAs whose increased abundance upon ZSWIM8 knockdown produces a clear effect on their predicted targets, we believe that such a strategy would not be appropriate in the context of global ZSWIM8 depletion. ZSWIM8 KD simultaneously stabilizes multiple miRNAs and can also indirectly alter the expression of others, leading to complex combinatorial and secondary transcriptomic effects (see **Appendix Fig. S1C,D**). Under these conditions, changes in target mRNA abundance cannot be unambiguously attributed to a single miRNA, making bulk RNA-seq–based target derepression an unreliable primary filtering strategy.

For this reason, we adopted a sequential approach: first, identifying candidate TDMD substrates using abundance-aware and statistically stringent criteria; second, isolating the post-transcriptional component via the net-effect metric; and third, assessing biological impact through miR-eCLIP and trigger-specific perturbation experiments (e.g., NREP manipulation), which provide a more controlled and interpretable readout.

Importantly, the revised manuscript now combines FDR-adjusted statistics (DESeq2/EdgeR), defined L2FC thresholds, and net-effect filtering, thereby restricting the dataset to robust and biologically interpretable TDMD substrates.

R1.Q4. *miR 29b-3p differs from miR 29a and miR 29c-3p by five and four nucleotides, respectively. Which of these differences contributes to the differential effect of ZSWIM8 knockdown?*

R1.R4. The differential behaviour of miR-29b-3p compared to miR-29a-3p and miR-29c-3p upon ZSWIM8 depletion is consistent with the established structural requirements of TDMD. As described in the **Introduction** (**page 3,** first page of the **introduction**), TDMD requires a specific pairing of the miRNA:trigger duplex, involving seed pairing, a central unpaired region (bulge), and extended complementarity at the 3' end of the miRNA (Sheu-Gruttadauria, J. et al. Mol Cell 2019). This is directly illustrated in **Fig. 3I**, which shows the alignment between miR-29b-3p and the NREP TDMD trigger, highlighting the extended 3'-end complementarity required for efficient TDMD. These structural features have been defined in seminal studies of viral and endogenous TDMD systems from our and other laboratories (Cazalla et al., Science 2010; Kleaveland et al., Cell 2018; Bitetti et al., Nat Struct Mol Biol 2018; Ghini et al., Nat Commun 2018). Importantly, sequence differences between miR-29b-3p and the other miR-29 family members primarily reside within the 3' region of the mature miRNA, which is the portion critically involved in TDMD-specific pairing. Previous work has demonstrated that even limited nucleotide changes in the miRNA 3' end can markedly impair TDMD efficiency, while leaving canonical seed-dependent target repression largely unaffected. In other words, mutations that disrupt 3' complementarity abolish TDMD without necessarily altering the canonical regulatory activity of the miRNA. In line with this mechanism, NREP functions as a TDMD trigger preferentially for miR-29b-3p, consistent with compatible 3'-end pairing, whereas other miR-29 family members are less susceptible (Bitetti et al., Nat Struct Mol Biol 2018). The differential sensitivity of miR-29b-3p has also been observed in previous studies using ZSWIM8 loss-of-function approaches (Shi et al Genome Res 2023), and is fully consistent with the mechanistic framework typical of TDMD.

In the updated manuscript we show that in breast cancer miR-29b-3p displays strong correlation with NREP-dependent miR-29 targets, while miR-29a-3p correlation is only moderate or low (**Figs. 4G-I** and **Expanded Fig. EV3C-D**; see also R1.Q5)

R1.Q5. *The authors speculate repeatedly about the connection between TDMD and oncogenesis, but present no evidence showing that when the abundance of ZSWIM8 or a specific TDMD trigger is reduced that oncogenic progression is altered.*

R1.R5. We thank the reviewer for this important comment. We would like to clarify the scope of our claims. The manuscript does not propose that ZSWIM8 depletion per se alters oncogenic progression. Rather, we identify TDMD substrates in breast cancer cells and provide mechanistically supported examples of specific miRNA:trigger axes that modulate transcriptional plasticity and confer selective advantages in defined contexts.

In particular, we report two specific observations: (i) the NREP:miR-29b-3p axis is associated with EMT-related transcriptional states, and (ii) SERPINE1-mediated TDMD of miR-30c confers measurable tumour-related phenotypes, including effects on sphere-forming efficiency and increased resistance to paclitaxel treatment. These conclusions are based

on molecular and functional perturbation experiments in breast cancer cell models. We agree that direct demonstration of altered oncogenic progression *in vivo* would require dedicated phenotypic studies, which are beyond the scope of the present work, and this limitation is explicitly discussed in the revised manuscript (e.g. **Discussion,** page **17,** bottom **:**"*These examples* ***suggest that TDMD may contribute to oncogenesis*** *by promoting the selective degradation of anti-tumor miRNAs in specific cellular context*"; and page **19** "*Furthermore, it was recently found that NREP modulates tumor growth and metastasis in breast cancer model. Whether* ***this phenotype is specifically mediated by TDMD remains to be established****, but it is tempting to speculate that TDMD plays a part.*"; and page **19** "*Although* ***we did not perform direct phenotypic assays to assess tumor progression*** *in this study, the association between NREP, miR-29b-3p repression and EMT-related subpopulations is consistent with prior in vivo and mechanistic studies and* ***provides a strong rationale for further functional validation****.*").

Regarding ZSWIM8 expression, published data (Shi et al Genome Res 2023), our RNA-seq analyses, and publicly available Human Protein Atlas (HPA) datasets consistently show that ZSWIM8 is broadly expressed across tissues, cancer subtypes, BRCA cell lines or cell subpopulations (see at this link: https://www.proteinatlas.org/ENSG00000214655-ZSWIM8). These observations suggest that TDMD specificity in breast cancer is unlikely to depend on differential ZSWIM8 expression per se. Rather, our data indicate that TDMD activity depends on the context-dependent expression of specific miRNA substrates and their cognate RNA triggers, as reflected in **Figs. 1** and **2**, where only selected miRNAs are susceptible to TDMD in a given cellular model despite comparable ZSWIM8 levels. In this work, ZSWIM8 is therefore primarily used as a tool to unmask TDMD substrates rather than proposed as a cancer driver.

To further support the cancer relevance of the NREP:miR-29b-3p axis, we extended our analysis to patient cohorts. We and others previously reported a strong anti-correlation between NREP and miR-29b across multiple cancer (Simeone et al., NAR 2022). Building on this observation, we investigated whether the transcriptional circuitry linking NREP, miR-29b-3p, and the set of miR-29b-3p targets identified by miR-eCLIP and responsive to NREP perturbation (**Figs. 3E** and **Fig. 4E–F**) could also be reconstructed in patients. To this end, we analysed TCGA BRCA datasets in which miRNA and mRNA expression were profiled from the same samples. We observed a strong positive correlation between NREP expression and the specific set of target genes we identified (new panels in **Fig. 4G–I**), and a robust anti-correlation of the targets with miR-29b-3p. Importantly, this anti-correlation was significantly stronger than that observed for miR-29a-3p, which is co-transcribed with miR-29b-3p but is not regulated by NREP (new panels **Fig. 4G–I; Expanded Fig. EV3C–D**). This regulatory relationship extends beyond BRCA (**Expanded Fig. EV3E**), further supporting the relevance of the NREP:miR-29b-3p TDMD axis in cancer-associated transcriptional programs. These analyses and their interpretation are now explicitly described in the revised **Results** section.

Together, these data indicate that while we do not claim a direct demonstration of altered oncogenic progression, we provide mechanistic, functional, and patient-level evidence that specific TDMD interactions are active in breast cancer and linked to biologically relevant transcriptional states and tumour-related phenotypes.

R1.Q6. *Similarly, the authors propose that TDMD plays a role in human cancer, yet all of their studies are confined to immortalized, breast cancer cell lines in vitro. To make such an assertion would require, for example, evidence correlating mutations in ZSWIM8 or a TDMD trigger to clinical cancer progression or showing a change in oncogenesis upon altering the function or abundance of ZSWIM8 or a TDMD trigger in an appropriate orthotopic mouse model****.***

R1.R6. We thank the reviewer for this important comment and agree that our original wording may have overstated the general implication of TDMD in human cancer. We have revised the manuscript to avoid overinterpretation and to better define the scope of our conclusions. Specifically, we do not claim that TDMD is a driver of oncogenic progression, nor that we have demonstrated altered tumor progression in vivo upon manipulation of ZSWIM8 or specific TDMD triggers. Our main conclusion is that TDMD is active in breast cancer cells and specific TDMD axes intersect with cancer-relevant transcriptional programs, transcriptional plasticity, and tumor-associated phenotypes. Demonstrating a direct impact of TDMD on tumor initiation or progression in orthotopic mouse models would represent an important future direction, but it is not required for the mechanistic claims made in the present study.

Independent studies have established tumor-related roles for both components of the NREP:miR-29b axis. NREP has been reported to promote tumor growth and metastatic phenotypes in TNBC models (Ruan et al. Cell Death Discov., 2024). In parallel, miR-29b has been characterized as a tumor suppressor in breast cancer, where its repression enhances metastasis and extracellular matrix remodeling (Chou et al., *Nat. Cell Biol.*, 2013). The latter study, from the Zena Werb laboratory, provided detailed mechanistic evidence linking reduced miR-29 activity to pro-tumorigenic phenotypes. Our contribution is mechanistic. We identify a TDMD-based regulatory layer that functionally connects NREP expression to post-transcriptional repression of miR-29b-3p in breast cancer cells. While TDMD of miR-29 family members was previously described in developmental contexts ((Bitetti et al., Nat Struct Mol Biol 2018), we show that this circuitry is active in human breast cancer and is associated with transcriptionally plastic tumor subpopulations characterized by high trigger expression. We place NREP under the control of EMT-associated transcriptional programs (e.g., TWIST1), thereby linking TDMD to cancer cell plasticity. This provides a complementary and symmetric layer of regulation to previously described transcriptional control of miR-29b-3p (e.g., via GATA3 in luminal contexts), integrating transcriptional and post-transcriptional mechanisms in

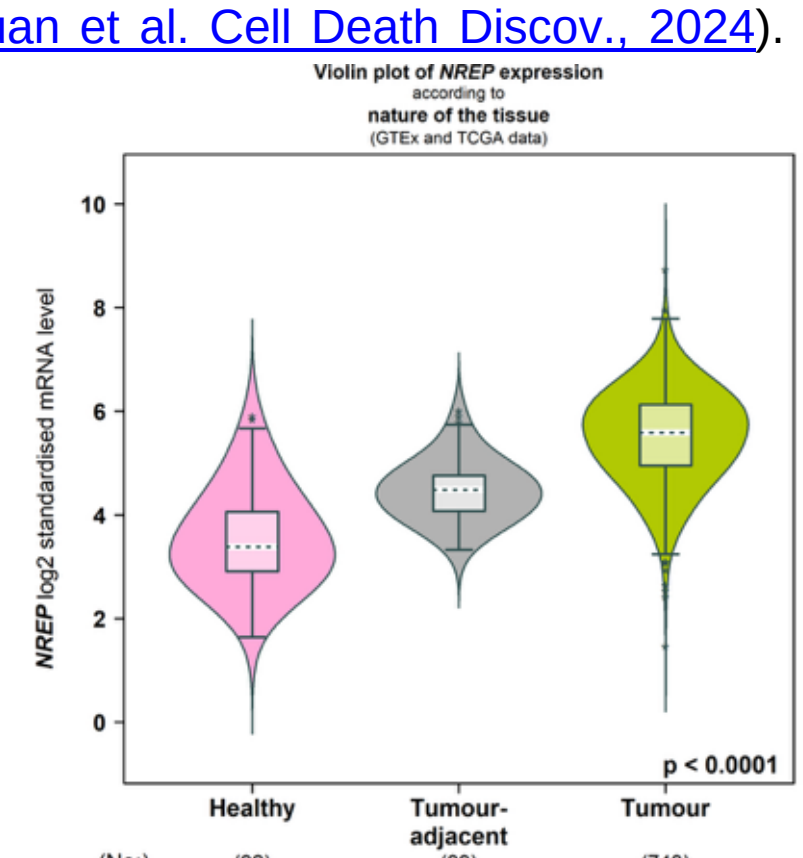

tumor biology.

Finally, regarding genetic evidence, the absence of recurrent mutations in ZSWIM8 or in miRNA degradation elements (MDEs) is not unexpected. In the cases described here, TDMD triggers (e.g., NREP) act to repress miRNAs with tumor-suppressive roles. Under this model, loss-of-function mutations in MDE regions would not be positively selected. Instead, increased expression of the trigger transcripts would be expected to confer selective advantages, consistent with the upregulation observed for NREP in breast tumors, shown here in the violin plot.

In conclusion, while in vivo validation remains an important future direction, our data provide mechanistic, functional, and cancer-dataset evidence that TDMD operates in human breast cancer and contributes to cancer-associated regulatory plasticity, without claiming direct demonstration of altered oncogenic progression.

***R1.Q7** Although the finding that SERPINE1 triggers TDMD of miR 30c-5p independently from ZSWIM8 or proteasome function is interesting, the authors identify the phenomenon, then move on to other experiments. In fact, the data for tailing and trimming of miR 30c-5p resides in a 2018 paper from these authors. The finding of ZSWIM8-independent TDMD merits an entire research paper explaining at least some of this novel mechanism.*

R1.R7. We agree that a full mechanistic dissection of ZSWIM8-independent TDMD would warrant dedicated follow-up work. However, we emphasize that demonstrating that a *bona fide* TDMD event (SERPINE1:miR-30c-5p) can occur independently of ZSWIM8 defines a conceptual boundary of ZSWIM8-based substrate discovery and is therefore critical for the correct interpretation of our dataset. More broadly, it indicates that miRNA degradation cannot be restricted to a single ZSWIM8-dependent pathway. Importantly, our conclusion that miR-30c-5p degradation is ZSWIM8- and proteasome-independent is supported by multiple orthogonal experiments across different breast cancer models, and the phenomenon is conserved across species (mouse and human), suggesting that the underlying regulatory logic is evolutionarily conserved. To clarify the relevance and framing of this result, we expanded the **Discussion** in the revised manuscript (**pages** 20-21).

ZSWIM8-independent mechanism:
It is worth mentioning that we are not alone in reporting ZSWIM8-independent TDMD-like mechanisms. A recent study by Ortega et al. (NAR 2024) demonstrated that Cyrano-like and Serpine1-like TDMD sites can be artificially repurposed to induce degradation of other miRNAs (including miR-155) even in the absence of ZSWIM8. Together with our findings, on endogenous triggers, this supports the existence of alternative TDMD routes that do not rely on the canonical ZSWIM8–ubiquitin–proteasome axis. Notably, miR-155 has been reported to exhibit relatively rapid turnover (Marzi et al., Genome Res 2017). Thus, it is tempting to speculate that miR-155 may also undergo ZSWIM8-independent decay, although the endogenous trigger responsible for such a mechanism has not yet been identified.

Experimental constraints:
In principle, an unbiased dissection of the ZSWIM8-independent mechanism would require a screening strategy analogous to the sensor-based approach used for the miR-7–Cyrano system (Han et al., Science 2020). However, implementing a similar strategy for miR-30c-5p is technically impractical. The mir-30 family comprises multiple members sharing an identical seed sequence, and the mature miR-30c-5p sequence is generated from two independent genomic loci. Consequently, canonical miR-30c sensors would be confounded by repression from other mir-30 family members, rendering genome-wide screening non-interpretable without extensive multi-locus genetic engineering. We now clarify these constraints in the revised **Discussion** (**page** 20).

Tailing and trimming:
Finally, we would like to clarify the relationship between TDMD, 3′ tailing/trimming and ZSWIM8 dependence. While tailing and trimming are classical hallmarks of miRNAs undergoing high turnover, recent studies demonstrate that 3′ tailing is not required for ZSWIM8-dependent TDMD, as blocking tailing (e.g., PAPD4 knockdown) does not prevent substrate degradation (Han et al., Science 2020; Shi et al., Science 2020; Kleaveland et al.,Cell 2018). The reviewer refers to our previous work (Ghini et al., Nat Commun 2018), where SERPINE1-mediated miR-30c-5p degradation in mouse fibroblasts was associated with an increased proportion of adenylated isoforms. However, adenylation has not been demonstrated to function as an obligate “death mark”. As clarified in the revised Discussion, and consistent with the established protective role of adenylation in other miRNA contexts, the apparent enrichment of adenylated forms may reflect selective degradation of non-adenylated isoforms (i.e., kinetic enrichment of surviving species), potentially involving 3′-to-5′ exonucleases such as the exosome or DIS3L2-dependent pathways. We have expanded the **Discussion** to clearly distinguish established observations from mechanistic hypotheses (**page** 21).

In conclusion, we believe that the identification of a conserved, ZSWIM8-independent TDMD event represents an important conceptual advance within the scope of the present work, while acknowledging that a comprehensive mechanistic dissection remains an important direction for future investigation.

Minor Points

***R1.minor (1)** It is unclear why the authors introduce a new term, "matched strand," for what is normally called passenger or miR* strand of a miRNA duplex.*

R1.R8(1). We thank the reviewer for pointing this out. We introduced the term “matched strand” specifically in **Fig. 2, Expanded View Fig. EV1** and **Appendix Fig. S2**, where both strands of each miRNA duplex were quantified to calculate the Net Effect metric.

In this analytical context, the conventional terminology "guide" and "passenger" could be misleading, as TDMD can in principle affect either strand of the duplex. For example, when the TDMD substrate corresponded to the canonical guide strand, we quantified the corresponding passenger strand; conversely, when the substrate was a strand annotated as "passenger" (e.g., miR-26a-2-3p or miR-106b-3p in **Fig. 2E**), we quantified the opposite strand (the complementary "guide"). The term "matched strand" was therefore used to indicate the complementary strand of the same duplex without implying functional hierarchy. Outside of this specific analytical context, miRNAs are consistently referred to by their full names (e.g., miR-30c-5p), to avoid ambiguity.

We hope this clarifies the rationale behind this terminology. To avoid potential confusion, we have added a note in the figure legend *("matched strand" miRNA refers to the opposite strand from the same miRNA duplex")*. explicitly stating that "matched strand" refers to the opposite strand of the same miRNA duplex, irrespective of guide/passenger annotation.

R1.*minor *(2) *Is there an error in this sentence, "...were prioritized as high-confidence TDMD substrates based on the concordance between increased expression upon ZSWIM8 depletion and reduced binding to their targets"? Do the authors mean increased binding to their targets? Why would binding be decreased when miRNA levels increase?*

R1.R8(2). We thank the reviewer for spotting this inconsistency. The original sentence indeed contained an error in wording. As correctly noted, increased miRNA levels upon ZSWIM8 depletion are expected to result in increased target occupancy, not reduced binding. We have now corrected the sentence as follows: "*Three miRNAs (miR-7-5p, miR-29b-3p and miR-33a-5p) were found in common with the substrates identified previously (**Fig. 3E**) and were prioritized as high-confidence TDMD substrates based on the concordance between increased miRNA expression (sRNA-seq) and increased target occupancy (miR-eCLIP) upon ZSWIM8 depletion*."

R1.minor (3) *The term "miRNA activity" is confusing. eCLIP measures miRNA target site occupancy, not miRNA function. It would be helpful to specify which biochemical process is being discussed instead of using the vague word "activity."*

R1.R8(3). We agree that the term "miRNA activity" may be imprecise in this context, as miR-eCLIP measures target site occupancy within AGO complexes rather than downstream functional repression. To avoid ambiguity, we have replaced the term "activity" with "target occupancy" throughout the text and in the relevant figures (**Fig. 3** and **Expanded View Fig. EV2**).

R1.minor (4) *The figures are tiny. Once further reduced in a journal, they will become unreadable.*

R1.R8(4). To address this issue, we have revised the layout and dimensions of several figure panels throughout the manuscript. In particular, the original **Figure 4** has been split into two separate figures (**new Fig. 4** and **new Fig. 5**) allowing for larger and clearer panels. In addition, **Fig. 2C–D**, **Expanded View Fig. EV1** and **Appendix Fig. S2** have been reformatted to improve clarity and readability, also in response to Reviewer 2's suggestion. Overall, multiple panels across the manuscript have been resized and reorganized to ensure optimal legibility at journal scale.

R1.minor (5) *Please indicate the number of biologically independent replicates for the various high-throughput sequencing experiments.*

R1.R8(5). We thank the reviewer for this important request. We have verified that the number of biologically independent replicates (N) is now explicitly indicated in each relevant figure panel and/or legend for all high-throughput sequencing experiments.
Specifically:

- **Fig. 1**: sRNA-seq (N = 3)
- **Fig. 3** and Expanded View Fig. EV2: miR-eCLIP (N = 2); sRNA-seq upon trigger overexpression (N = 2)
- **Fig. 4** and Expanded View Fig. EV3: RNA-seq upon NREP knockdown (N = 3)
- **Fig. 5**: TWIST1 ChIP-seq (N = 2)
- **Fig. 6** and **Expanded View Fig. EV4**: scRNA-seq (HMLE sorted cells N = 2; HMLE bulk N = 2; SUM159PT N = 2, one representative dataset shown as in Nadalin et al., 2024; BT-549 N = 1)
- **Fig. 7**: RNA-seq upon SERPINE1 MDE knockout (N = 5 independent clones)

We have ensured that this information is consistently reported in the figure legends to improve transparency and reproducibility.

Referee #2:

Rubolino and colleagues study the mechanisms of TDMD across several breast cancer cell lines. They identify miRNAs regulated by TDMD and identify their triggers. The study begins broadly and then narrows its focus to two specific examples in breast cancer: NREP:miR-29b-3p and SERPINE1:miR-30. The first interaction is shown to depend on ZSWIM8, whereas the second appears ZSWIM8-independent. Overall, the experiments are well designed and well carried out. Moreover, the manuscript is well written with good visualizations, which makes it easy to follow. In summary, the manuscript, in my opinion, definitely hits the significance threshold for this journal (and other journals of even higher impact factor). However, a few questions and suggestions should be considered/clarified before publication.

Major concerns

R2.Q1. *In **Figure 1C**, individual data points and error bars are missing for the control samples. Without them, it is difficult to interpret why T47D is reported as p < 0.05 while showing less variability than MCF-7 (p < 0.001).*

R2.R1. We thank the reviewer for this comment. In **Fig. 1C**, both ZSWIM8 and miR-7 levels are normalized to the corresponding control condition for each cell line; therefore, the control bar is displayed as a fixed reference value (set to 1) rather than as an independently plotted dataset. For this reason, individual data points and error bars are not shown for the control bar in this panel. The variability of the control samples is reported in **Fig. 1B**, where absolute expression levels are displayed with individual replicates in SUM159PT cells. The apparent difference in statistical significance between T47D (p < 0.05) and MCF-7 (p < 0.001) reflects the higher variability observed in the respective control measurements.

To avoid confusion, we have revised **Fig. 1C.** The legend now explicitly states that the control bar serves as a normalization reference. In addition, the control bar is now displayed in grey to clearly distinguish it from the experimental conditions.

R2.Q12. Figure 1E: Roughly 25 % (31 miRNAs) are detected in multiple cell lines. Why are the remaining miRNAs observed in only one line? Is their expression cell-line-specific?

R2.R1. We thank the reviewer for this question. Following **Reviewer#1**'s suggestion, **Fig. 1E** has been revised to include only miRNAs showing a L2FC > 0.3 (n = 63). Among these, 17 miRNAs are common, defined as upregulated in more than one cell line, of which 14 meet the FDR < 0.05 threshold (see updated **Fig. 1E)**. The remaining miRNAs are observed in only a single cell line for two main reasons: (i) lack of detectable expression in the other cell lines, or (ii) detectable expression without significant upregulation upon ZSWIM8 perturbation. This distinction is important for interpretation. In several cases, miRNAs are expressed across multiple models, but are regulated only in specific cell lines, suggesting that TDMD requires not only miRNA expression but also the corresponding trigger, which may be expressed in a cell-line-specific manner, as discussed in the manuscript.

To clarify this point, we performed a systematic expression analysis across all cell lines. The results are now reported in **Dataset EV1** (updated) and visualized in the **new Appendix Fig. S1B** (shown below). The main text has been revised accordingly (**page** 5, bottom) to explicitly indicate that only 17 of the 63 miRNAs are regulated in multiple cell lines, reflecting differences in miRNA expression and/or trigger availability across models.

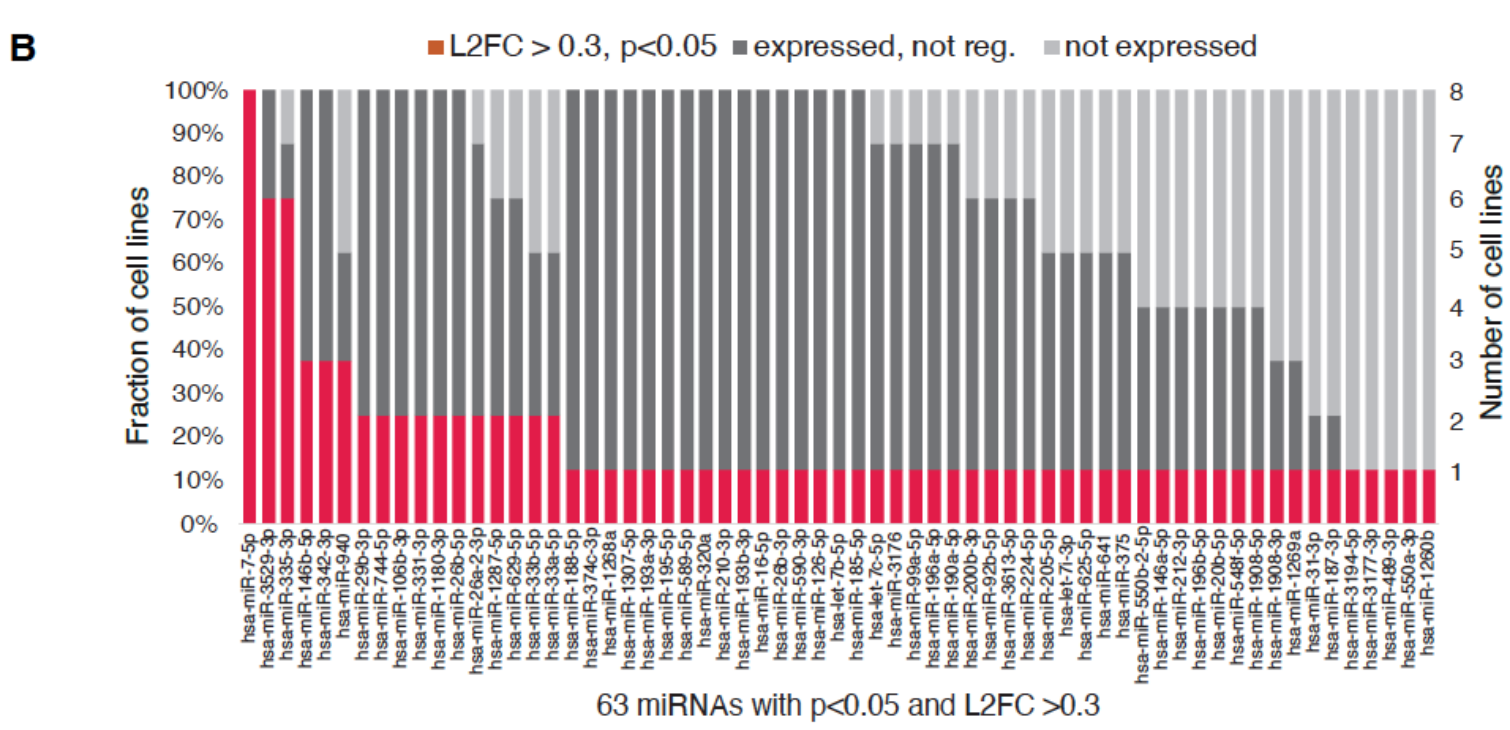

Appendix Figure S1: Analysis of miRNAs regulated upon ZSWIM8 KD. *B) The stacked bar chart reports the number of cell lines (and the proportion) in which each of the 63 TDMD miRNA substrates was found as upregulated (red, L2FC >0.3, pvalue <0.05), expressed but not upregulated (dark grey, >10 CPM) or not expressed (light grey, <10 CPM). MiRNAs are ordered according to the number of lines where they were found as upregulated (e.g. miR-7-5p 8/8, 100%).*

R2.Q3. Some experiments in Figure 2CD miss measurements for the 'matched miRNA' and/or 'hostgene' (the pink and blue dots). Can the authors clarify why these are missing?

R2.R1. We thank the reviewer for raising this point. The missing pink (matched miRNA) and/or blue (pri-miRNA/host gene) dots in **Fig. 2C, D** (and in the related **Appendix Fig. S2**) reflect cases in which the corresponding species were not detectable rather than omitted. Specifically, missing measurements arise under three conditions: (i) the matched miRNA strand was not detected above threshold (RPM >10) in the small RNA-seq dataset; (ii) the corresponding pri-miRNA could not be reliably quantified by RT–qPCR; or (iii) the miRNA is intergenic and therefore does not have an annotated host gene.

To clarify this issue and avoid potential misunderstanding, we have added an explicit note in the legend of **Fig. 2** (and **Appendix Fig. S2**) stating that missing dots correspond to species that were not detectable under the experimental conditions.

R2.Q4. The authors state that miR-7 interacts with LINC00632, but all peaks are located within the CDR1as exon. How can one distinguish interaction with LINC00632 from binding to the spliced CDR1as transcript? Isn't it more likely that miR-7 interacts with CDR1as directly?

R2.R4. We agree that the miR-7 interaction maps to the CDR1as exon, and that it is highly likely that miR-7 interacts directly with CDR1as, as previously reported. From a technical standpoint, miR-eCLIP does not allow discrimination between i) the linear LINC00632 transcript and ii) the circular CDR1as RNA derived from it, as both share the same genomic sequence. For this reason, we refer to the interaction using the composite annotation "LINC00632/CDR1as". However, as correctly noted, all miR-7 chimeric peaks localize to the CDR1as exon (**Expanded Fig. EV2F**). To avoid ambiguity, we have revised the wording in the **Results** section to more clearly state that the detected interaction signal corresponds to CDR1as-derived sequences (page **8**: "*this finding suggests that miR-7 target occupancy is highly restricted in breast cancer cells by different mechanisms involving two ncRNAs, Cyrano and CDR1as*").

R2.Q5. The authors state that NREP is a potential trigger for miR-29b-3p based on TDMDfinder, but in Figure 3F NREP appears only in the control binding plot, but not in ZSWIM8-KD. In addition, the text refers to Figure 3G when arguing that COL5A1 and SPARC lack TDMD hallmarks, but that figure does not seem to support the claim.

R2.R5. We thank the reviewer for this careful examination of the data.

NREP signal in ZSWIM8-depleted cells.
The reviewer is correct that NREP-derived miR-29b-3p chimeric reads are detected in control cells, but not in the ZSWIM8-KD samples (**Fig. 3F**). This likely reflects a slightly lower sensitivity of chimeric peak detection in the ZSWIM8-KD samples, as shown in **Expanded View Fig. EV2B–C**.

Importantly, miR-eCLIP data are not limited to chimeric reads but also include AGO2-bound non-chimeric peaks. Upon inspection of the non-chimeric AGO2 peaks in ZSWIM8-KD cells, we identified a significant NREP peak (chr5:111,730,329–111,730,478), which overlaps the annotated miR-29b-3p MDE (chr5:111,730,360–111,730,367). The coincidence of the AGO2 peak with the MDE strongly supports persistent binding of miR-29b-3p to NREP in ZSWIM8-depleted cells, even if chimeric reads were not recovered at sufficient depth for peak calling. Thus, the absence of a detectable chimeric peak in ZSWIM8-KD does not indicate loss of interaction, but rather reduced recovery of chimeric reads.

COL5A1 and SPARC as potential triggers.
The reviewer is also correct that **Fig. 3G** does not explicitly display the structural TDMD features of individual transcripts such as SPARC or COL5A1. **Fig. 3G** schematically summarizes canonical TDMD hallmarks and reports the number of predicted high-confidence triggers expressed and AGO2-bound in ZSWIM8-KD cells, but it does not depict individual alignment architectures.

SPARC and COL5A1 were not included among the high-confidence TDMD triggers because they do not meet the structural criteria required for TDMD (in particular extended 3′ pairing beyond the seed region), as defined in our previous work (Simeone et al., NAR 2018) and reported in **Dataset EV2** ("TDMD HC triggers" sheet). While these transcripts show increased binding upon ZSWIM8 depletion, their predicted interaction architecture is consistent with canonical target repression rather than TDMD.

To improve clarity, we have revised the text in the corresponding section (**page** 9) to explicitly state that SPARC and COL5A1 do not fulfill TDMD structural hallmarks and were therefore not considered candidate triggers.

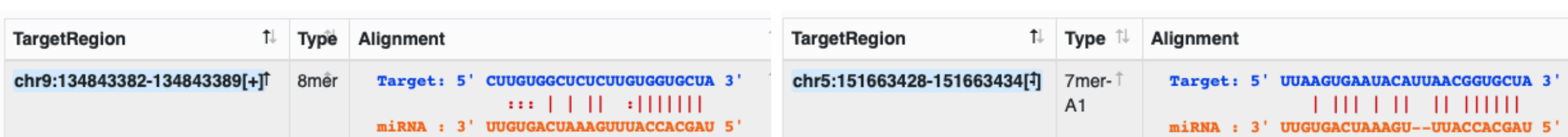
<table>
<tr><th>TargetRegion</th><th>Type</th><th>Alignment</th></tr>
<tr><td>chr9:134843382-134843389[+]</td><td>8mer</td><td>Target: 5' CUUGUGGCUCUCUUGUGGUGCUA 3'
::: | | || :|||||||
miRNA : 3' UUGUGACUAAAGUUUACCACGAU 5'</td></tr>
</table>

<table>
<tr><th>TargetRegion</th><th>Type</th><th>Alignment</th></tr>
<tr><td>chr5:151663428-151663434[-]</td><td>7mer-A1</td><td>Target: 5' UUAAGUGAAUACAUUAACGGUGCUA 3'
| ||| | || || ||||||
miRNA : 3' UUGUGACUAAAGU--UUACCACGAU 5'</td></tr>
</table>

SPARC and COL5A1 as potential triggers. *TargetScan-predicted miR-29b-3p binding sites within SPARC (left) and COL5A1 (right). The alignments show canonical seed pairing (7mer-1A and 8mer, respectively, shown at miRNA's 5' end), but lack extended 3' complementary pairing beyond the seed region, a structural requirement typically associated with TDMD triggers.*

R2.Q6. For the scRNA-seq experiment in the HMLE cells, the authors sequenced two FACS-sorted replicates and one 'bulk'. If I understand it correctly, the brown cluster in Figure S5A represents the sorted population. If so, why do the FACS-sorted replicates still contain so many other cells? Could this be explained by imperfect sorting, or did the authors use any integration approach in Seurat that forces cluster alignment?

R2.R6. We thank the reviewer for this careful evaluation of the scRNA-seq data. The reviewer is partially correct: the brown cluster in **Expanded View Fig. EV4A** represents a mesenchymal/stem-enriched transcriptional state that is specifically identifiable in the $CD44^{high}/CD24^{low}$ FACS-sorted population. However, the sorted replicates reproducibly contain cells in multiple transcriptional states. This does not primarily reflect imperfect sorting, but rather the intrinsic transcriptional heterogeneity of the $CD44^{high}/CD24^{low}$ subpopulation. Indeed, $CD44^{high}/CD24^{low}$ cells represent a rare fraction (~1% of bulk HMLE cells), and even after enrichment, this compartment remains transcriptionally heterogeneous at single-cell resolution. Importantly, the purpose of the sorting strategy in this study was not to obtain a transcriptionally homogeneous population, but to enrich for a rare state that becomes detectable and quantifiable at single-cell resolution following enrichment.

Regarding data processing, the scRNA-seq libraries were aggregated using the Cell Ranger "*aggr*" function and normalized for sequencing depth. No Seurat-based integration, anchor-based alignment, or batch-correction procedure was applied. Thus, the observed clustering structure reflects the underlying biological heterogeneity rather than computational alignment.

To avoid potential misunderstanding, we have clarified this point in the **Methods** section (**page** 29).

Minor concerns

R2.Q7. ZSWIM8 is misspelled once as "ZWSIM8" at the end of the introduction.

R2.R7. The misspelling of "ZSWIM8" as "ZWSIM8" at the end of the Introduction has been corrected in the revised manuscript.

R2.Q8. The legend for Figure 7C is missing and should be added**.**

R2.R8. We thank the reviewer for this comment. The legend corresponding to **panel 7C** was present in the original submission but was merged with panels D–E in the figure legend, which may have caused confusion.

In the revised manuscript (now **Fig. 8C–E**), the legend has been clearly reformatted to explicitly indicate panel C. The corresponding text reads: "(**C–D–E**) Expression levels of ZSWIM8 (**panel C**), miR-7-5p (**panel D**) and miR-30c-5p (**panel E**) were measured by RT-qPCR in HeLa cells with (+) or without (–) perturbation of ZSWIM8 by CRISPRi for 7 days. Data are shown separately for each individual sgRNA."

Referee #3:

In this manuscript, Nicassio and colleagues study the process of target-directed miRNA decay (TDMD) in breast cancer cells. TDMD has been described relatively recently, and it is arguably one of the most timely and active lines of research into RNA biology. While the main mechanism of TDMD via ZSWIM8-mediated AGO proteosomal degradation has been recently described, the propensity of TDMD in different physiological systems, the nature of TDMD triggers, and the biological functions of individual TDMD interactions between miRNAs and their targets are all only partially understood. The manuscript describes very rigorous work with important progress on all three of these, and as such, it is an excellent fit for The EMBO Journal. The manuscript is very well written and the data are presented mostly clearly. The TDMD targets identified in the various breast cancer cell lines will be an excellent resource for further study; also, the overall modest increases in miRNA levels observed with a few clear exceptions put important bounds on TDMD relevance. The characterization of miR-33 triggers will also open interesting research directions, as the potential within-pathway feedback loop is very interesting. The ZSWIM-independent SERPINE1-miR-30 axis will also be very interesting to study further out of scope of this study.

I have only minor suggestions before recommending to publish:

R3Q1. In Figure 2D, it is not clear enough what is going on, in particular in the 2nd box with the net effects. Adding more boundaries (e.g., a y axis) or making the figure larger will make it easier to understand**.**

R3.R1. We thank the reviewer for this helpful suggestion. To improve clarity of the Net Effect representation in **Fig. 2C–D**, we have added an explicit Y-axis and introduced a grey-shaded box indicating the threshold used to define miRNAs passing the Net Effect cutoff. In addition, the figure panels have been enlarged to enhance readability.

These modifications have been applied to the updated **Fig. 2C–D** and the related figures (**Expanded View Fig. EV1A–B** and **Appendix Fig. S2A–D**). The revised layout facilitates the visual interpretation of the Net Effect metric and its cutoff.

2. In Figure 4O - The other genes in the region beside NREP should be shown.

R3.R2. We thank the reviewer for this helpful suggestion. We have included in the genomic view of **Fig. 5I** (previous **Fig. 4O**) the annotated genes (Gencode v47) within the displayed locus. In the 110.9–111.4 Mb (0.5 Mb) interval, NREP is the only gene detectably expressed in our dataset. The antisense transcript NREP-AS1 and STARD4-AS1 are also present in the locus, but were not expressed in our samples. Further downstream genes, including EPB41L4A and EPB41L4A-AS1, lie outside the displayed genomic window and are therefore not shown.

3. Figure 6- Can the fold changes in miR-30 be compared to the fold changes induced by ZSWIM8-mediated decay? In particular how it compares to the net effect threshold of 0.3 used in the previous parts of the manuscript?

R3.R3. We thank the reviewer for this important point. In the SERPINE1 MDE-deletion experiment (now **Fig. 7E**), we explicitly measured not only the upregulation of mature miR-30c-5p (and miR-30b-5p), but also the corresponding primary transcripts (pri-miR-30c1 and pri-miR-30c2) and the miR-30c passenger strands across five independent MDE KO clones and WT controls.

During re-analysis of the data and verification of the source data , we identified an error in the original **Fig. 6E** (now **Fig. 7E**), where pri-miRNA qPCR results were erroneously duplicated in the passenger miRNA panel. We have now fixed this error and provide the updated panel in the revised manuscript. The previous and corrected versions are shown below for transparency.

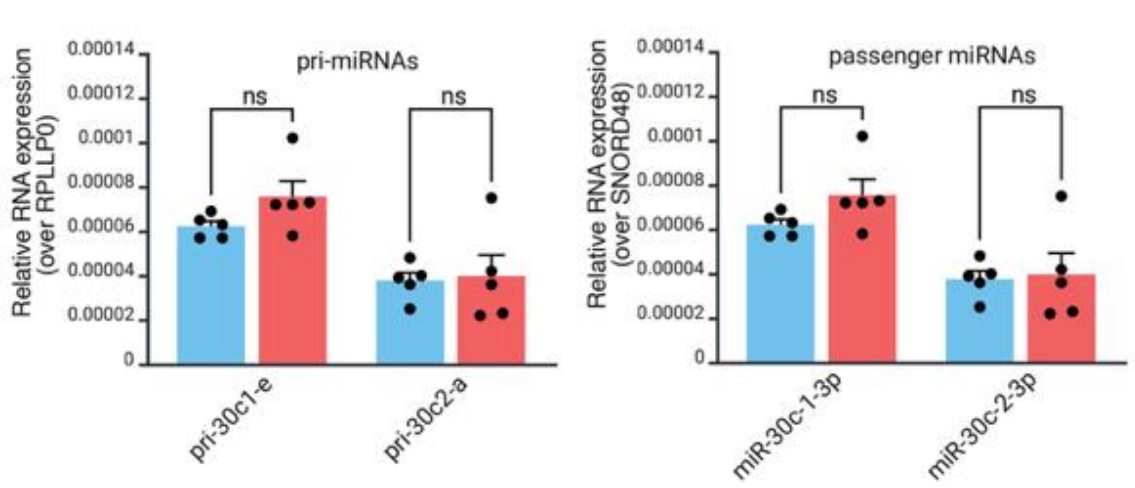

Fig. 6E *old panel with duplicated data*

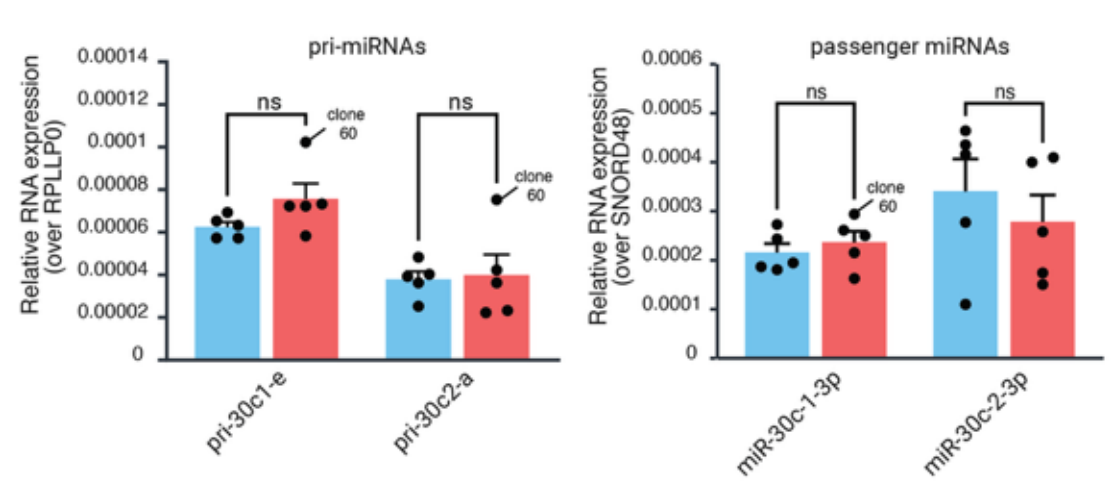

Fig. 7E *new panel with correct data for passenger miRNAs*

Quantitively, the pri-miRNAs and passenger miRNAs are not significantly regulated and show small average fold changes (pri-miR-30c1-e = 0.27; pri-miR-30c2-a = 0.07; miR-30c-1-3p = 0.13; miR-30c-2-3p = -0.29 L2FC), whereas mature miR-30c-5p is significantly regulated (**L2FC = 0.48**). Thus, applying the same logic used for the ZSWIM8-dependent contexts, the post-transcriptional stabilization account for the observed increase in mature miR-30c-5p, consistent with relief of TDMD upon MDE removal.

We noticed some inter-clonal variability at transcriptional level and at phenotypic level (e.g. clone 60, see **Fig. 7E, G**) which is consistent with reported molecular variability and transcriptional noise following CRISPR-mediated editing (Prajapat et al., Nucleic Acids Research 2025).

We amended the text (**Results,** pages **14-15)** to comment about this point: "*Primary precursors of miR-30c-5p were not regulated, although some variability was observed between clones (e.g. KO-60,* ***Fig. 7E****), in line with recent findings that CRISPR editing introduces some molecular and transcriptional variability" (Prajapat et al., Nucleic Acids Research 2025)*.

Dear Dr Nicassio, dear Dr Marzi,

Thank you again for submitting your amended manuscript (EMBOJ-2025-122495R) to The EMBO Journal. Please accept my apologies for the protraction with the reassessment due to delayed expert input at this time of the year and detailed discussion in the editorial team. Your revised study was sent back to the referees for their scientific reassessment, and we have received detailed re-reports from both, which I attach below. As you will see, the referees state that the work has been substantially enhanced by the revisions and they are now in favour of publication, pending minor revision.

Thus, we are pleased to inform you that your manuscript has been accepted in principle for publication in The EMBO Journal.

Please carefully consider the remaining point still raised by referee #1 by adjusting the data statistics applied to FDR <0.05 throughout and emphasizing orthogonal validation measures in the manuscript text where appropriate.

Also, we now need you to take care of a number of minor issues related to formatting and data annotation as detailed in a separate information during the next days. There will also be requests by our production team for Source Data provision sent shortly.

As you might have seen on our web page, every paper at the EMBO Journal now includes a 'Synopsis', displayed on the html and freely accessible to all readers. The synopsis includes a 'model' figure as well as 2-5 one-short-sentence bullet points that summarize the article. I would appreciate if you could provide this figure and the bullet points.

Please submit a revised version of the manuscript using the link enclosed below.

Thank you again for giving us the chance to consider your manuscript for The EMBO Journal. I look forward to receiving your final revised version of the manuscript fro swift acceptance and online publication.

Kind regards,

Daniel Klimmeck

Daniel Klimmeck PhD
Senior Editor
The EMBO Journal

Please remember: Digital image enhancement is acceptable practice, as long as it accurately represents the original data and conforms to community standards. If a figure has been subjected to significant electronic manipulation, this must be noted in the figure legend or in the 'Methods' section. The editors reserve the right to request original versions of figures and the original images that were used to assemble the figure.

Referee #1:

Reviewer: Phillip D. Zamore

I was surprised by the authors' responses to my original critique, and I continue to find the paper lacking in statistical rigor and appropriate depth of analysis for its claims.
Here are the things that still concern me.

(1) The authors argue that a t-test uncorrected for multiple hypothesis testing could be valid for small numbers of experimental conditions by citing a single paper from 2022. Multiple hypothesis testing is always required when the number of hypotheses is >1. That is why one often sees the Bonferroni correction applied to ANOVA analyses. Moreover, my concerns about statistical rigor have only grown based the number of replicates for each experiments (information provided in the response to reviewers).
(2) Reporting FDR values that are >0.05 but <0.2 is not appropriate. I don't recall ever seeing <0.2 used as a statistical cutoff for high-throughput sequencing data. For comparison, both Shi et al. (Science 2020) and Akulenko et al. (Gene 2025) used an adjusted p-value cutoff <0.0001 for changes in miRNA abundance; Jones et al. (Genes Dev 2022) used FDR < 0.05. In using FDR <0.2, the authors are suggesting that they are satisfied reporting data in which one in every five observations is a false discovery. The more important implication is that the study is underpowered and far more replicates are needed or that the number of meaningful changes in miRNA abundance is quite small.
The question of which data is statistically meaningful can be easily decide by using the method described by Wang & Bartel (BMC Bioinformatics 2023), who have implemented a statistical framework specifically for detecting TDMD-regulated miRNAs. In retrospect, I should have asked the authors to filter their data using this approach, which was designed to separate primary and secondary effects from ZSWIM8 knockdown.
(3) I don't understand the rationale for reporting log2 fold-changes in miRNA abundance using an 0.3 cutoff. This is almost certainly less than the interanimal variation in vivo and unlikely to be biologically meaningful. Many TDMD targets have fold-changes much greater than 1.23 (log2 fold-change = 0.3); confining the analysis to reasonable effect sizes would raise my confidence in the interpretation of the data.
(4) If the authors state that "The manuscript does not propose that ZSWIM8 depletion per se alters oncogenic progression. Rather, we identify TDMD substrates in breast cancer cells and provide mechanistically supported examples of specific miRNA:trigger axes that modulate transcriptional plasticity and confer selective advantages in defined contexts." If this is the goal, then it should be made clear in the title and abstract. The current title gives the impression that TDMD is important for oncogenic progression.

Minor Concerns
(1) What is an "NREP:miR-29b-3p axis"? I honestly don't understand this phrase.
(2) Given that my lab co-discovered TDMD (Ameres et al., Science 2010), I have tried to provide advice to the authors that reflects my expertise. I don't doubt that TDMD can cause small changes in miRNA abundance. Whether such changes have biological meaning is a completely different question. In zebrafish, deletion of Cyrano, one of the most potent TDMD triggers known, has no detectable phenotype in vivo (Goudarzi et al., eLife 2019). I urge the authors to be conservative in making claims about the importance of minor differences in miRNA abundance.
(3) The authors write that "TDMD requires a specific pairing of the miRNA:trigger duplex, involving seed pairing, a central unpaired region (bulge), and extended complementarity at the 3' end of the miRNA." I agree and refer the authors to Ameres et al. (Science 2010) and Xie et al. (Nat Methods 2012).

Referee #2:

Thanks for the thorough revision and for addressing all my comments so carefully. I'm satisfied with the changes and have no further concerns. I now recommend publication.

Referee #3:

The authors have addressed my comments in a satisfactory manner and I can now recommend publication in EMBO J.

Dear Dr Nicassio, dear Dr Marzi,

Further to below, I attach the mentioned list of formatting and data presentation changes required for your final resubmission.

Please let us know any time should you have additional questions related.

Best regards,

Daniel Klimmeck

Daniel Klimmeck PhD
Senior Editor
The EMBO Journal

******

Formatting changes required for the revised version of the manuscript:

>> Author information: Please spell out the full names of the authors in the manuscript text as they should be published.

>> Author Contributions: Remove the author contributions information from the manuscript text. Note that CRediT has replaced the traditional author contributions section as of now because it offers a systematic machine-readable author contributions format that allows for more effective research assessment. and use the free text boxes beneath each contributing author's name to add specific details on the author's contribution.

More information is available in our guide to authors.
https://www.embopress.org/page/journal/14602075/authorguide

>> Please correct the order and headings of the sections in the manuscript text to: Abstract / Introduction / Results / Discussion / Methods / Data Availability / Acknowledgements / Disclosure and Competing Interests Statement / References / Figure Legends / Expanded View Figure Legends

>> 'Statistical Analysis' and related Methods-data filtering: Please carefully consider the remaining point still raised by referee #1 by adjusting the data statistics applied to FDR <0.05 and emphasizing orthogonal validation measures in the manuscript text where appropriate. Indicate whether independent replicates were used for the complementary RT-qPCR analysis.

>> References: please adjust reference format to EMBO Journal format, 10 authors et al.

>> BioRender: Please remove the information about BioRender from the legends and compile it in a dedicated section in the Methods using this format:
Graphics:
(some of the... OR Figure #... OR synopsis) Graphics were created with BioRender.com.

>> Funding: merge with Acknowledgments; please ensure that the information in our system and in the manuscript text match. Please move the funders entered into the comments section underneath the list of funders to the line-by-line list in our system, as the information in the comments is not transferred to our publisher and cannot be linked upon publication in PubMed.

>> Dataset EV legends: Please remove the word doc with the EV dataset legends and add each legend to the corresponding dataset file instead, e.g. add it to the README tabs.

>> Please provide source data for the study as to the separate request e-mail. Source data should be uploaded as one (zipped) file per figure.

>> Figure callouts: There are callouts for Appendix Figures S1-4 but no appendix file has been uploaded. Please correct.

Further information is available in our Guide For Authors: https://link.springer.com/journal/44318/submission-guidelines

al

Referee #1:

I was surprised by the authors' responses to my original critique, and I continue to find the paper lacking in statistical rigor and appropriate depth of analysis for its claims.
Here are the things that still concern me.

Major Concerns

(1) *The authors argue that a t-test uncorrected for multiple hypothesis testing could be valid for small numbers of experimental conditions by citing a single paper from 2022. Multiple hypothesis testing is always required when the number of hypotheses is >1. That is why one often sees the Bonferroni correction applied to ANOVA analyses. Moreover, my concerns about statistical rigor have only grown based the number of replicates for each experiments (information provided in the response to reviewers).*

(2) *Reporting FDR values that are >0.05 but <0.2 is not appropriate. I don't recall ever seeing <0.2 used as a statistical cutoff for high-throughput sequencing data. For comparison, both Shi et al. (Science 2020) and Akulenko et al. (Gene 2025) used an adjusted p-value cutoff <0.0001 for changes in miRNA abundance; Jones et al. (Genes Dev 2022) used FDR < 0.05. In using FDR <0.2, the authors are suggesting that they are satisfied reporting data in which one in every five observations is a false discovery. The more important implication is that the study is underpowered and far more replicates are needed or that the number of meaningful changes in miRNA abundance is quite small. The question of which data is statistically meaningful can be easily decide by using the method described by Wang & Bartel (BMC Bioinformatics 2023), who have implemented a statistical framework specifically for detecting TDMD-regulated miRNAs. In retrospect, I should have asked the authors to filter their data using this approach, which was designed to separate primary and secondary effects from ZSWIM8 knockdown.*

We thank the reviewer for raising this important point. In response to this comment, and in agreement with the editor's recommendation, we have now revised the manuscript by applying FDR < 0.05 throughout the main analysis. Specifically, we removed FDR < 0.2 as a criterion from the main figures and text, and revised **Figures 1** and **2** so that the main analysis now focuses on miRNAs supported by FDR < 0.05 and on a small subset of recurrent candidates supported across multiple cell lines and by independent orthogonal evidence.

The broader discovery dataset remains fully reported in **Dataset EV1** for transparency, including all statistical values, but the revised manuscript now clearly distinguishes between the initial discovery phase and the high-confidence TDMD substrate set carried forward into downstream analyses.

Concerning the distinction between primary and secondary effects, we agree that this is essential for interpreting ZSWIM8 perturbation experiments. Our analytical strategy addresses this point through a sequential validation framework. Candidate miRNAs identified by small RNA-seq are subjected to a second-tier analysis in which precursor levels, host-gene expression and matched-strand abundance are measured to exclude indirect transcriptional or processing effects. This step was systematically applied to the candidates reported in **Figure 2** and related **Expanded View** and **Appendix** figures, and was specifically designed to separate post-transcriptional regulation from secondary effects.

The small RNA-seq analysis provides the discovery layer, whereas the high-confidence set is defined only after statistical filtering and independent validation of post-transcriptional regulation. In addition, the key miRNA:trigger interactions explored in depth are supported by orthogonal approaches, including miR-eCLIP to assess AGO target occupancy and targeted perturbation experiments in independent cellular contexts.

Thus, the revised manuscript no longer relies on FDR < 0.2 to define the main TDMD substrate set, and the central conclusions remain based on FDR < 0.05 supported and/or independently validated regulatory events.

(3) *I don't understand the rationale for reporting log2 fold-changes in miRNA abundance using an 0.3 cutoff. This is almost certainly less than the interanimal variation in vivo and unlikely to be biologically meaningful. Many TDMD targets have fold-changes much greater than 1.23 (log2 fold-change = 0.3); confining the analysis to reasonable effect sizes would raise my confidence in the interpretation of the data.*

We agree that fold-change alone is not sufficient to establish biological relevance. For this reason, the L2FC > 0.3 threshold was never used as a standalone criterion, but rather as an initial discovery filter combined with statistical significance and subsequent orthogonal validation. In agreement with the reviewer's recommendation, we have further increased the stringency of the analysis by applying FDR < 0.05 throughout the main dataset. Candidate TDMD substrates are therefore prioritized on the combination of effect size, statistical support and independent validation, rather than on fold-change magnitude alone.

The final set of 19 HD TDMD miRNAs is characterized by an average L2FC of 0.97 and a normalized net-effect of 0.79; these values, much higher than the threshold of 0.3, should be more in line with the expectations of the reviewer on "reasonable effect sizes".

Importantly, the biological conclusions of the study are not derived from the magnitude of miRNA stabilization per se. Instead, they are based on orthogonally validated TDMD interactions for which post-transcriptional regulation, AGO target occupancy and trigger dependency were independently demonstrated. The revised manuscript emphasizes this distinction clearly throughout the **Results** and **Discussion** sections.

Thus, while we agree that modest fold changes should be interpreted cautiously, we note that the central conclusions of the study rely on independently validated TDMD events rather than on the L2FC threshold itself.

(4) *If the authors state that "The manuscript does not propose that ZSWIM8 depletion per se alters oncogenic progression. Rather, we identify TDMD substrates in breast cancer cells and provide mechanistically supported examples of specific miRNA:trigger axes that modulate transcriptional plasticity and confer selective advantages in defined contexts." If this is the goal, then it should be made clear in the title and abstract. The current title gives the impression that TDMD is important for oncogenic progression.*

We thank the reviewer for this comment. In the revised version, we have further revised the text, including the Abstract and Discussion sections to more explicitly distinguish between the identification of TDMD substrates and the functional characterization of selected miRNA:trigger interactions. The manuscript now more clearly states that ZSWIM8 depletion is primarily used as an experimental tool to identify TDMD substrates, and that the biological conclusions are derived from the mechanistically validated TDMD interactions investigated in detail.

We have also revised the text to avoid implying that ZSWIM8 depletion per se drives oncogenic progression. Instead, the revised manuscript emphasizes that specific TDMD interactions intersect with transcriptional plasticity and tumor-associated phenotypes in breast cancer models, while acknowledging that direct demonstration of altered tumor progression in vivo is beyond the scope of the present study.

We believe that these revisions provide a more conservative and accurate representation of the study’s conclusions and better align the framing of the manuscript with the evidence presented.

Minor Concerns

1. *What is an "NREP:miR-29b-3p axis"? I honestly don't understand this phrase.*

The expression “NREP:miR-29b-3p axis” refers to the regulatory interaction in which NREP acts as a TDMD trigger that promotes the degradation of miR-29b-3p, thereby influencing the expression of miR-29b-3p target genes. To improve clarity for readers unfamiliar with the terminology, we now explicitly define this interaction when first introduced (see page 12).

2. *Given that my lab co-discovered TDMD (Ameres et al., Science 2010), I have tried to provide advice to the authors that reflects my expertise. I don't doubt that TDMD can cause small changes in miRNA abundance. Whether such changes have biological meaning is a completely different question. In zebrafish, deletion of Cyrano, one of the most potent TDMD triggers known, has no detectable phenotype in vivo (Goudarzi et al., eLife 2019). I urge the authors to be conservative in making claims about the importance of minor differences in miRNA abundance.*

We agree that modest changes in miRNA abundance should not automatically be equated with biological significance. In the revised manuscript, we have further adopted a conservative interpretation of the data by restricting the main analysis to FDR < 0.05-supported substrates and by more clearly distinguishing discovery-stage observations from experimentally validated TDMD interactions.

In conclusion, while in vivo validation remains an important future direction, our data provide mechanistic, functional, and cancer-dataset evidence that TDMD operates in human breast cancer and contributes to cancer-associated transcriptional programs and cellular phenotypes, without claiming direct demonstration of altered oncogenic progression.

3. *The authors write that "TDMD requires a specific pairing of the miRNA:trigger duplex, involving seed pairing, a central unpaired region (bulge), and extended complementarity at the 3' end of the miRNA." I agree and refer the authors to Ameres et al. (Science 2010) and Xie et al. (Nat Methods 2012)*

We thank the reviewer for this suggestion. Ameres et al. (Science 2010), which established the phenomenon of target-directed miRNA tailing and trimming and is directly relevant to the structural requirements discussed in the manuscript, is already cited in the Introduction and related sections.

We carefully considered the inclusion of Xie et al. (Nat Methods 2012). While that study provides important evidence that artificial Tough Decoy (TuD) RNAs can induce miRNA degradation in vivo, the references currently cited in this section, including Ameres et al. (Science 2010), Sheu-Gruttadauria et al. (Mol Cell 2019), Cazalla et al. (Cell 2010), de la Mata et al. (EMBO Reports 2015), and Bitetti et al. (EMBO Journal 2018), more directly address endogenous or viral TDMD mechanisms and the miRNA:target pairing features that define canonical TDMD. We have therefore retained the current citation set, which we believe more accurately reflects the specific point being discussed.

Referee #2:

Thanks for the thorough revision and for addressing all my comments so carefully. I'm satisfied with the changes and have no further concerns. I now recommend publication.

Referee #3:

The authors have addressed my comments in a satisfactory manner and I can now recommend publication in EMBO J.

We thank Referees #2 and #3 for their positive evaluation of the revised manuscript and for recommending publication. We appreciate their constructive comments, which have helped improve the quality and clarity of the study.

2nd Revision - Editorial Decision 22nd Jul 2026

Dear Dr Nicassio,

Thank you for submitting the revised version of your manuscript. I have now evaluated your amended manuscript and concluded that the remaining minor concerns have been sufficiently addressed.

I am thus pleased to inform you that your manuscript has been accepted for publication in the EMBO Journal.

Your manuscript will be processed for publication by EMBO Press. It will be copy edited and you will receive page proofs prior to publication. Please note that you will be contacted by Springer Nature Author Services to complete licensing and payment information.

Please note that it is The EMBO Journal policy for the transcript of the editorial process (containing referee reports and your response letter) to be published as an online supplement to each paper. If you do NOT want this, you will need to inform the Editorial Office via email immediately. More information is available here: https://www.embopress.org/transparent-process#Review_Process

You may qualify for financial assistance for your publication charges - either via a Springer Nature fully open access agreement or an EMBO initiative. Check your eligibility: https://www.embopress.org/page/journal/14602075/authorguide#chargesguide

Should you be planning a Press Release on your article, please notify the production team via this form in order to coordinate publication and release dates: https://forms.cloud.microsoft/e/eH4A8JVP6g

If you have any questions, please do not hesitate to contact the Editorial Office. Thank you for your contribution to The EMBO Journal.

Thank you again for this contribution to The EMBO Journal and congratulations on a successful publication! Please consider us again in the future for your most exciting work.

Best regards,

Daniel Klimmeck

Daniel Klimmeck, PhD
Senior Editor
The EMBO Journal
EMBO
Postfach 1022-40
Meyerhofstrasse 1
D-69117 Heidelberg
contact@embojournal.org

*****
Please note that it is The EMBO Journal policy for the transcript of the editorial process (containing referee reports and your response letters) to be published as an online supplement to each paper. If you should prefer removal of any referee-only figures included in the point-by-point response(s), e.g. because they may still be used for future publication or because they have been reproduced from published work by others, please do let us know immediately via response email.

More information is available here: https://link.springer.com/partners/embo-press/editorial-policies#Peer%20review

EMBO Press Author Checklist

Corresponding Author Name: FRANCESCO NICASSIO	
Journal Submitted to: The EMBO Journal	
Manuscript Number: EMBOJ-2025-122495	

USEFUL LINKS FOR COMPLETING THIS FORM
The EMBO Journal - Author Guidelines
EMBO Reports - Author Guidelines
Molecular Systems Biology - Author Guidelines
EMBO Molecular Medicine - Author Guidelines

Reporting Checklist for Life Science Articles (updated January
This checklist is adapted from Materials Design Analysis Reporting (MDAR) Checklist for Authors. MDAR establishes a minimum set of requirements in transparent reporting in the life sciences (see Statement of Task: 10.31222/osf.io/9sm4x). Please follow the journal's guidelines in preparing your
Please note that a copy of this checklist will be published alongside your article.

Abridged guidelines for figures
1. Data
The data shown in figures should satisfy the following conditions:
- the data were obtained and processed according to the field's best practice and are presented to reflect the results of the experiments in an accurate and unbiased manner.
- ideally, figure panels should include only measurements that are directly comparable to each other and obtained with the same assay.
- plots include clearly labeled error bars for independent experiments and sample sizes. Unless justified, error bars should not be shown for technical
- if n<5, the individual data points from each experiment should be plotted. Any statistical test employed should be justified.
- Source Data should be included to report the data underlying figures according to the guidelines set out in the authorship guidelines on Data

2. Captions
Each figure caption should contain the following information, for each panel where they are relevant:
- a specification of the experimental system investigated (eg cell line, species name).
- the assay(s) and method(s) used to carry out the reported observations and measurements.
- an explicit mention of the biological and chemical entity(ies) that are being measured.
- an explicit mention of the biological and chemical entity(ies) that are altered/varied/perturbed in a controlled manner.
- the exact sample size (n) for each experimental group/condition, given as a number, not a range;
- a description of the sample collection allowing the reader to understand whether the samples represent technical or biological replicates (including how many animals, litters, cultures, etc.).
- a statement of how many times the experiment shown was independently replicated in the laboratory.
- definitions of statistical methods and measures:
  - common tests, such as t-test (please specify whether paired vs. unpaired), simple χ^2 tests, Wilcoxon and Mann-Whitney tests, can be unambiguously identified by name only, but more complex techniques should be described in the methods section;
  - are tests one-sided or two-sided?
  - are there adjustments for multiple comparisons?
  - exact statistical test results, e.g., P values = x but not P values < x;
  - definition of 'center values' as median or average;
  - definition of error bars as s.d. or s.e.m.

Please complete ALL of the questions below.
Select "Not Applicable" only when the requested information is not relevant for your study.

Materials

Newly Created Materials	Information included in the manuscript?	In which section is the information available? (Reagents and Tools Table, Materials and Methods, Figures, Data Availability Section)
New materials and reagents need to be available; do any restrictions apply?	Yes	Reagents and Tools Table, Materials and Methods

Antibodies	Information included in the manuscript?	In which section is the information available? (Reagents and Tools Table, Materials and Methods, Figures, Data Availability Section)
For **antibodies** provide the following information:<br>- Commercial antibodies: RRID (if possible) or supplier name, catalogue number and or/clone number<br>- Non-commercial: RRID or citation	Yes	Reagents and Tools Table, Materials and Methods

DNA and RNA sequences	Information included in the manuscript?	In which section is the information available? (Reagents and Tools Table, Materials and Methods, Figures, Data Availability Section)
**Short novel DNA or RNA including primers, probes**: provide the sequences.	Yes	Reagents and Tools Table, Materials and Methods, Dataset EV6

Cell materials	Information included in the manuscript?	In which section is the information available? (Reagents and Tools Table, Materials and Methods, Figures, Data Availability Section)
**Cell lines:** Provide species information, strain. Provide accession number in repository **OR** supplier name, catalog number, clone number, and/**OR** RRID.	Yes	Reagents and Tools Table, Materials and Methods
**Primary cultures:** Provide species, strain, sex of origin, genetic modification status.	Not Applicable	
Report if the cell lines were recently **authenticated** (e.g., by STR profiling) and tested for mycoplasma contamination.	Yes	Materials and Methods

Experimental animals	Information included in the manuscript?	In which section is the information available? (Reagents and Tools Table, Materials and Methods, Figures, Data Availability Section)
**Laboratory animals or Model organisms:** Provide species, strain, sex, age, genetic modification status. Provide accession number in repository **OR** supplier name, catalog number, clone number, **OR** RRID.	Not Applicable	
**Animal observed in or captured from the field:** Provide species, sex, and age where possible.	Not Applicable	
Please detail **housing and husbandry conditions**.	Not Applicable	

Plants and microbes	Information included in the manuscript?	In which section is the information available? (Reagents and Tools Table, Materials and Methods, Figures, Data Availability Section)
**Plants:** provide species and strain, ecotype and cultivar where relevant, unique accession number if available, and source (including location for collected wild specimens).	Not Applicable	
**Microbes:** provide species and strain, unique accession number if available, and source.	Not Applicable	

Human research participants	Information included in the manuscript?	In which section is the information available? (Reagents and Tools Table, Materials and Methods, Figures, Data Availability Section)
If collected and within the bounds of privacy constraints report on age, sex and gender or ethnicity for all study participants.	Not Applicable	

Core facilities	Information included in the manuscript?	In which section is the information available? (Reagents and Tools Table, Materials and Methods, Figures, Data Availability Section)
If your work benefited from core facilities, was their service mentioned in the acknowledgments section?	Yes	Acknowledgments

Design

Study protocol	Information included in the manuscript?	In which section is the information available? (Reagents and Tools Table, Materials and Methods, Figures, Data Availability Section)
If study protocol has been **pre-registered, provide DOI in the manuscript**. For clinical trials, provide the trial registration number **OR** cite DOI.	Not Applicable	
Report the **clinical trial registration number** (at ClinicalTrials.gov or equivalent), where applicable.	Not Applicable	

Laboratory protocol	Information included in the manuscript?	In which section is the information available? (Reagents and Tools Table, Materials and Methods, Figures, Data Availability Section)
Provide DOI OR other citation details if **external detailed step-by-step protocols** are available.	Not Applicable	

Experimental study design and statistics	Information included in the manuscript?	In which section is the information available? (Reagents and Tools Table, Materials and Methods, Figures, Data Availability Section)
Include a statement about **sample size** estimate even if no statistical methods were used.	Yes	Materials and Methods
Were any steps taken to minimize the effects of subjective bias when allocating animals/samples to treatment (e.g. **randomization procedure**)? If yes, have they been described?	Not Applicable	
Include a statement about **blinding** even if no blinding was done.	Yes	Materials and Methods
Describe **inclusion/exclusion criteria** if samples or animals were excluded from the analysis. Were the criteria pre-established?<br><br>If sample or data points were omitted from analysis, report if this was due to attrition or intentional exclusion and provide justification.	Not Applicable	Materials and Methods
For every figure, are **statistical tests** justified as appropriate? Do the data meet the assumptions of the tests (e.g., normal distribution)? Describe any methods used to assess it. Is there an estimate of variation within each group of data? Is the variance similar between the groups that are being statistically compared?	Yes	Materials and Methods

Sample definition and in-laboratory replication	Information included in the manuscript?	In which section is the information available? (Reagents and Tools Table, Materials and Methods, Figures, Data Availability Section)
In the figure legends: state number of times the experiment was **replicated** in laboratory.	Yes	Figures, Materials and Methods
In the figure legends: define whether data describe **technical or biological replicates**.	Yes	Figures, Materials and Methods

Ethics

Ethics	Information included in the manuscript?	In which section is the information available? (Reagents and Tools Table, Materials and Methods, Figures, Data Availability Section)
Studies involving **human participants**: State details of **authority granting ethics approval** (IRB or equivalent committee(s), provide reference number for approval.	Not Applicable	
Studies involving **human participants**: Include a statement confirming that **informed consent** was obtained from all subjects and that the experiments conformed to the principles set out in the WMA Declaration of Helsinki and the Department of Health and Human Services Belmont Report.	Not Applicable	
Studies involving **human participants:** For publication of **patient photos**, include a statement confirming that consent to publish was obtained.	Not Applicable	
Studies involving experimental **animals**: State details of **authority granting ethics approval** (IRB or equivalent committee(s), provide reference number for approval. Include a statement of compliance with ethical regulations.	Not Applicable	
Studies involving **specimen and field samples:** State if relevant **permits** obtained, provide details of authority approving study; if none were required, explain why.	Not Applicable	

Dual Use Research of Concern (DURC)	Information included in the manuscript?	In which section is the information available? (Reagents and Tools Table, Materials and Methods, Figures, Data Availability Section)
Could your study fall under dual use research restrictions? Please check biosecurity documents and list of **select agents and toxins** (CDC): https://www.selectagents.gov/sat/list.htm	Not Applicable	
If you used a select agent, is the security level of the lab appropriate and reported in the manuscript?	Not Applicable	
If a study is subject to dual use research of concern regulations, is the name of the **authority granting approval and reference number** for the regulatory approval provided in the manuscript?	Not Applicable	

Reporting

The MDAR framework recommends adoption of discipline-specific guidelines, established and endorsed through community initiatives. Journals have their own policy about requiring specific guidelines and recommendations to complement MDAR.

Adherence to community standards	Information included in the manuscript?	In which section is the information available? (Reagents and Tools Table, Materials and Methods, Figures, Data Availability Section)
State if relevant guidelines or checklists (e.g., **ICMJE, MIBBI, ARRIVE, PRISMA**) have been followed or provided.	Not Applicable	
For **tumor marker prognostic studies,** we recommend that you follow the **REMARK** reporting guidelines (see link list at top right). See author guidelines, under 'Reporting Guidelines'. Please confirm you have followed these guidelines.	Not Applicable	
For **phase II and III randomized controlled trials**, please refer to the **CONSORT** flow diagram (see link list at top right) and submit the CONSORT checklist (see link list at top right) with your submission. See author guidelines, under 'Reporting Guidelines'. Please confirm you have submitted this list.	Not Applicable	

Data Availability

Data availability	Information included in the manuscript?	In which section is the information available? (Reagents and Tools Table, Materials and Methods, Figures, Data Availability Section)
Have **primary datasets** been deposited according to the journal's guidelines (see 'Data Deposition' section) and the respective accession numbers provided in the Data Availability Section?	Yes	Data Availability Section
Were **human clinical and genomic datasets** deposited in a public access-controlled repository in accordance to ethical obligations to the patients and to the applicable consent agreement?	Not Applicable	
Are **computational models** that are central and integral to a study available without restrictions in a machine-readable form? Were the relevant accession numbers or links provided?	Not Applicable	
If publicly available data were reused, provide the respective **data citations in the reference list**.	Yes	Data Availability Section
